# Integrative Taxonomy of *Agaricus* subgen. *Pseudochitonia* in Arid Northwestern China: Species Diversity and Habitat-Associated Morphological Differentiation

**DOI:** 10.3390/jof12070512

**Published:** 2026-07-13

**Authors:** Zhengxiang Qi, Keqing Qian, Peisong Jia, Lili Shi, Dongmei Wu, Libo Wang, Xiao Li, Yu Li, Bo Zhang

**Affiliations:** 1 Sanjiang Fungal Industry Collaborative Innovation Center, Jilin Agricultural University, Changchun 130118, China; qzx7007@126.com (Z.Q.); 15670567112@163.com (K.Q.); sll031006@126.com (L.S.); wlblucky@126.com (L.W.); lxmogu@163.com (X.L.); 2 Engineering Research Center of Edible and Medicinal Fungi, Ministry of Education, Jilin Agricultural University, Changchun 130118, China; 3 College of Mycology, Jilin Agricultural University, Changchun 130118, China; 4 Industrial Development Institute for Plants, Animals and Fungi Integration of Biyang County, Biyang 463799, China; 5 Xinjiang Academy of Agricultural Sciences, Urumqi 830091, China; jps-fly@163.com; 6 Biotechnology Research Institute, Xinjiang Academy of Agricultural and Reclamation Sciences, Shihezi 832000, China; wdm0999123@sina.com

**Keywords:** *Agaricus*, Agaricaceae, biodiversity, integrative taxonomy, multilocus phylogeny, new species

## Abstract

Xinjiang, northwestern China, encompasses sharply contrasting desert–wetland and montane forest ecosystems, providing a natural setting to study species limits and habitat-associated morphological variation in *Agaricus* subgen. *Pseudochitonia*. We examined 303 specimens collected from 2021 to 2025 and generated 905 sequences across three loci (ITS, LSU, *tef1*). Species delimitation followed an integrative framework incorporating morphology, multilocus phylogeny, pairwise genetic distances, chemical reactions, and habitat data. We recognized ten species from Xinjiang. Among them, *Agaricus acanthosquamosus* is described as a new species in sect. *Bohusia*. It is recovered as a sister to *A. bohusii* and is distinguished by conspicuous squarrose spinose pileus scales, subglobose to broadly ellipsoid basidiospores, a solitary to scattered fruiting habit, and reddening context. We also document newly observed morphological variation in several previously described species. These include irregularly clavate cheilocystidia in *A. desjardinii*, clavate pleurocystidia in *A. subperonatus*, non-digitate pleurocystidial apices in *A. sinodeliciosus*, and habitat-associated macromorphological variation in *A. xanthodermus*. These observations extend the known morphological range of each taxon. Exploratory PCA, PERMANOVA, and trait comparisons revealed significant assemblage-level morphological structuring between the Ebinur Lake desert–wetland assemblage and the Qiongkushitai montane forest assemblage, notably in basidioma size, robustness, and basidiospore dimensions. In addition, we compiled a comprehensive identification key and species checklist for *Agaricus* subgen. *Pseudochitonia* in Xinjiang. These integrative results highlight both the diversity of subgen. *Pseudochitonia* in arid northwestern China and the value of combining morphological, ecological, and molecular evidence for accurate species delimitation.

## 1. Introduction

Morphology alone is often insufficient for species delimitation in macrofungi, particularly in groups where phenotypic traits vary with the environment or converge among unrelated lineages. In *Agaricus*, expanded sampling from both tropical and temperate regions has substantially reshaped infrageneric classification and revealed far greater phylogenetic diversity than was previously recognized from morphology alone [1,2,3]. Among the major lineages of *Agaricus*, subgen. *Pseudochitonia* is particularly species-rich and taxonomically challenging, comprising numerous sections and exhibiting high morphological variability across species distributed in a wide range of habitats [4,5]. In addition to its taxonomic complexity, subgen. *Pseudochitonia* includes economically important edible taxa, such as the cultivated button mushroom *A. bisporus* [6], as well as toxic *A. xanthodermus*, making accurate species delimitation relevant to both fungal biodiversity assessment and applied mycology [7,8,9].

The taxonomic difficulty of subgen. *Pseudochitonia* is primarily caused by pronounced morphological overlap and phenotypic convergence among closely related or superficially similar taxa. Diagnostic macro- and micromorphological characters, including pileus squamulation, annulus structure, context discoloration, odor, and chemical reactions, are informative only when interpreted in combination and within a phylogenetic framework. For example, species historically placed in or near sect. *Xanthodermatei* are characterized by combinations of yellowing reactions, phenolic odor, negative Schäffer’s reaction, and KOH response, yet the intensity of yellowing may vary among collections, and odor may change with basidioma development, maturation, and post-collection handling [10,11]. Similarly, studies of sect. *Bohusia*, sect. *Sanguinolenti*, and allied sections have shown that traditional morphological characters remain informative, but only when evaluated together with multilocus phylogeny and carefully circumscribed diagnostic traits [4,5]. Collectively, these issues highlight two major challenges: (i) unstable species delimitation caused by morphological overlap among closely related taxa, and (ii) insufficient regional sampling and incomplete documentation of species diversity in underexplored areas, particularly Central and Northwestern Asia.

Prior to the present study, documented records of *Agaricus* from Xinjiang were limited to fewer than ten species [12,13,14], leaving the diversity of this region substantially undercharacterized relative to its ecological complexity. Xinjiang provides an especially informative system in which to examine species boundaries due to its exceptional ecological heterogeneity. Across relatively short geographic distances, it contains sharply contrasting habitats, including montane conifer forests dominated by *Picea schrenkiana* Fisch. & C.A.Mey., riparian woodlands, desert steppes, sandy habitats, and inland saline wetlands. The western Tianshan *Picea* forests and the Ebinur Lake basin are sharply contrasting in vegetation structure, soil conditions, hydrology, and exposure, despite both occurring within arid northwestern China. This environmental contrast makes Xinjiang a useful region for studying *Pseudochitonia*. It allows us to examine species boundaries and to assess whether morphological variation is associated with forest and desert–wetland habitats.

In this study, we applied an integrative taxonomic framework to collections of *Agaricus* subgen. *Pseudochitonia* from Xinjiang, combining morphology, multilocus phylogeny, pairwise genetic distances, chemical reactions, fruiting habit, and habitat data. Our aims were to: (1) delimit the species represented in the Xinjiang material and determine their sectional placement within subgen. *Pseudochitonia*; (2) describe a new species and refine the morphological criteria for known species; and (3) compare morphological trait distributions between the Ebinur Lake and Qiongkushitai assemblages and evaluate whether these patterns provide ecological context for interpreting variation.

## 2. Materials and Methods

### 2.1. Specimen Sampling, Morphology, and Ecological Characterization

Fresh basidiomata of *Agaricus* subgen. *Pseudochitonia* were collected in the Xinjiang Uygur Autonomous Region, China, during field investigations conducted between 2021 and 2025. Fresh basidiomata were photographed in situ, and field notes were made for each collection, including habitat type, substrate, associated vegetation, growth habit, and geographic locality. After collection, specimens were dried and deposited in the Fungarium of Jilin Agricultural University (FJAU).

Macromorphological descriptions were based on fresh basidiomata. Color terms and codes followed Kornerup and Wanscher [15]. Chemical reactions were tested on fresh material whenever possible. For the KOH reaction, a 3% KOH solution was applied to the pileus surface. Schäffer’s reaction was tested by drawing intersecting lines on the pileus surface using two glass rods dipped separately in aniline and concentrated nitric acid [8,10,16].

Micromorphological observations focused on basidiospores, basidia, cheilocystidia, pleurocystidia (when present), pileipellis hyphae, and annular hyphae. Tissues from dried specimens were rehydrated in distilled water, mounted in 5% KOH, and stained with 1% Congo Red when necessary. Microscopic observations and measurements were made using a light microscope. At least 20 basidiospores, basidia, and cheilocystidia were measured whenever possible. Basidiospore measurements are presented as (a)b–c(d), where b–c includes at least 90% of the measured values and a and d represent the extreme values. For basidiospores, Q refers to the length/width ratio in lateral view, avL to average length, avW to average width, and avQ to mean Q. The notation [n/m/p] indicates that n spores were measured from m basidiomata of p collections [17,18,19]. Spore shape terminology follows the Q-value classification system of Bas (1969), as adopted in subsequent mycological standards, where Q represents the length/width ratio [20].

Scanning electron microscopy (SEM) was used to document basidiospore shape and surface features, following the general application of electron microscopy to *Agaricus* basidiospore and basidial morphology [21]. Mature basidiospores were removed from dried lamellae with a fine needle and mounted directly onto aluminum stubs using double-sided conductive carbon tape. The samples were sputter-coated with gold or gold–palladium for approximately 60–90 s. Spore preparations were examined using a ZEISS Sigma 300 field emission scanning electron microscope at an accelerating voltage of 5–10 kV. Images were acquired at magnifications of ×9000. Images were taken from mature, well-separated spores to confirm spore outline and surface smoothness.

To facilitate ecological comparison among species, each collection was additionally coded according to habitat type and fruiting habit. Habitats were classified into three broad categories: montane forest, desert–wetland/sandy habitat, and transitional riparian or disturbed habitat. Fruiting habit was coded as epigeous, shallowly buried, semi-hypogeous, or hypogeous.

### 2.2. DNA Extraction, PCR Amplification, and Sequencing

Genomic DNA was extracted from 10–20 mg of dried basidiomata tissue using a fungal genomic DNA extraction kit produced by Jiangsu Kangwei Century Biotechnology Co., Ltd., Taizhou, China, following the manufacturer’s instructions. Three loci were amplified for molecular analyses: the internal transcribed spacer (ITS) region, the nuclear large subunit ribosomal DNA (LSU), and the translation elongation factor 1-alpha (*tef1*) gene fragment. The primer pairs ITS1/ITS4, LR0R/LR5, and EF1-983F/EF1-1567R were used for ITS, LSU, and *tef1*, respectively (Table 1) [9,22,23,24].

PCR amplifications were performed in a total volume of 25 μL containing 13.5 μL ddH_2_O, 8 μL 2× Taq Master Mix, 1 μL of each primer, and 1.5 μL DNA template. The PCR program consisted of an initial denaturation at 95 °C for 5 min; 35 cycles of 95 °C for 40 s, 54 °C for 1 min, and 72 °C for 1 min; followed by a final extension at 72 °C for 10 min and storage at 4 °C. Amplification conditions generally followed Zhao [9], with minor adjustments when necessary for different loci or specimens.

PCR products were checked on 1% agarose gels stained with an appropriate nucleic acid dye. Successful amplicons were purified and sequenced by a commercial sequencing provider. Forward and reverse sequences were assembled and edited in BioEdit v.7.0.9 [25]. Newly generated sequences were deposited in GenBank.

### 2.3. Sequence Alignment and Phylogenetic Analyses

Newly generated sequences were compared with reference sequences retrieved from GenBank, with emphasis on taxa of *Agaricus* subgen. *Pseudochitonia* that were phylogenetically close to, or morphologically similar to, the Xinjiang collections [4,5,9,14,26,27,28,29,30,31,32,33]. Separate datasets were first assembled for ITS, LSU, and *tef1*, and these were subsequently concatenated into a combined ITS + LSU + *tef1* matrix for multilocus phylogenetic analyses.

Sequences for each locus were aligned using MAFFT v.7 [34] with default settings and manually adjusted where necessary in MEGA 11 [35]. Ambiguously aligned regions at the ends of the alignments were trimmed prior to concatenation. The final concatenated dataset comprised 2196 nucleotide positions and was partitioned by locus, including LSU, ITS, and *tef1*.

The optimal partitioning scheme and best-fit nucleotide substitution models were selected using PartitionFinder2 [36] under the Bayesian Information Criterion (BIC), with branch lengths linked and a greedy search algorithm [37]. The final partitioning scheme retained three locus-based subsets: LSU, ITS, and *tef1*. The best-fit models were GTR+I+G for LSU, HKY+I+G for ITS, and K80+G for *tef1*.

Phylogenetic analyses were conducted using Maximum Likelihood (ML) and Bayesian Inference (BI). ML analyses were performed in IQ-TREE 2 [38] using the partitioning scheme and substitution models selected by PartitionFinder2. Branch support was assessed with 1000 ultrafast bootstrap replicates [39]. BI analyses were performed in MrBayes v.3.2 [40] under the same partitioned framework. Two independent runs, each with four Markov chains, were carried out for 2,000,000 generations, with trees sampled every 100 generations. The first 25% of the sampled trees were discarded as burn-in. Model parameters were unlinked across partitions. Convergence between the two independent runs was confirmed by an average standard deviation of split frequencies (ASDSFs) < 0.01, as assessed in MrBayes, and by effective sample sizes (ESSs) > 200 for all model parameters after burn-in removal, as examined in Tracer v.1.7 [41]. A 50% majority-rule consensus tree was generated from the remaining trees.

Nodes with ML bootstrap support (MLBS) ≥ 70% and Bayesian posterior probability (BPP) ≥ 0.95 were considered well supported. Because the topologies recovered by ML and BI analyses were largely congruent, only the BI tree annotated with both MLBS and BPP values is presented. The final partitioning scheme and substitution models used for phylogenetic analyses are summarized in Table 2.

### 2.4. Species Delimitation and Pairwise Genetic Distance Analyses

#### 2.4.1. Species Delimitation Criteria

Species boundaries were evaluated using an integrative framework that combined multilocus phylogenetic placement, morphology, chemical reactions, pairwise sequence divergence, ecology, and habitat information [42,43]. For previously described taxa, identifications were based on congruence with reference or type-derived sequences when available, together with agreement in diagnostic morphological characters. For putatively novel species recognition, a stable combination of diagnostic morphological traits and an independent lineage recovered in the multilocus phylogeny in the multilocus phylogeny was required, preferably with strong support from ML bootstrap values and/or Bayesian posterior probabilities. Pairwise p-distances were used only as complementary evidence to evaluate the degree of molecular separation from the closest related or morphologically similar taxa. Because sequence divergence varied among loci and several species pairs lacked a strict barcode gap, no universal distance threshold was applied.

#### 2.4.2. Pairwise Genetic Distance Analyses

Pairwise genetic distances were calculated separately for ITS, LSU, and *tef1* using uncorrected p-distances in MEGA 11, with pairwise deletion of gaps and missing data.

For each focal species, mean within-species pairwise distances were calculated. Mean interspecific pairwise distances were then calculated between each focal species and a single closest relative, selected according to the multilocus phylogeny and relevant taxonomic literature.

Because ITS is the formal fungal barcode whereas LSU often provides lower resolution at the species level, distance values were interpreted comparatively across loci and used only as complementary evidence together with multilocus phylogeny, morphology, and ecology; no universal distance threshold was applied.

### 2.5. Morphological Morphospace and Trait Comparisons

To evaluate morphological differentiation between the two major habitat assemblages, we analyzed species from Ebinur Lake and Qiongkushitai separately. The Ebinur Lake assemblage included *A. sinodeliciosus*, *A. desjardinii*, and *A. padanus*, whereas the Qiongkushitai montane forest assemblage included *A. xanthodermus*, *A. cordillerensis*, *A. sylvaticus*, and *A. acanthosquamosus*. Nine traits were included in the analysis: eight primary measurements (pileus diameter, pileus context thickness, lamella width, stipe length, stipe width, annulus width, basidiospore length, and basidiospore width) and one derived trait (stipe length/width ratio). For each species, trait values were averaged at the collection level prior to analysis, and collection-level means were used as the unit of observation throughout (PCA and Kruskal–Wallis tests). All traits were standardized to zero mean and unit variance prior to PCA to remove the influence of measurement scale. PCA was performed in R v.4.4.0 using the prcomp function with scale = TRUE. Differences in trait distributions between the two habitat assemblages were tested using Kruskal–Wallis tests implemented in R. Mean values are provided as descriptive summaries. Results are reported as *H* statistics, *p* values, significance levels, and mean trait values for each habitat assemblage.

Permutational multivariate analysis of variance (PERMANOVA) was used to test whether overall morphological trait composition differed between the Ebinur Lake desert–wetland and the Qiongkushitai montane forest [44]. PERMANOVA was performed in R v.4.4.0 using the adonis2 function in the vegan package [45], based on Euclidean distances calculated from the standardized collection-level trait matrix, with 9999 permutations. The proportion of multivariate morphological variation explained by assemblage membership was estimated as *R*^2^.

## 3. Results

### 3.1. Sequence Dataset and Phylogenetic Overview

Phylogenetic analyses were based on three loci: ITS, LSU, and *tef1*. The datasets comprised 390 ITS, 328 LSU, and 334 *tef1* sequences in total (Table S1). Among these, 905 sequences were newly generated in this study (303 ITS, 301 LSU, and 301 *tef1*); the remaining sequences were retrieved from GenBank and represented morphologically similar or phylogenetically close taxa of *Agaricus* subgen. *Pseudochitonia*. The combined ITS + LSU + *tef1* alignment included representatives of 68 species, two unidentified lineages, and one outgroup taxon, *A. moellerianus* Bon. The final concatenated matrix was partitioned by locus, and the selected substitution models are summarized in Table 2.

**Figure 1:**
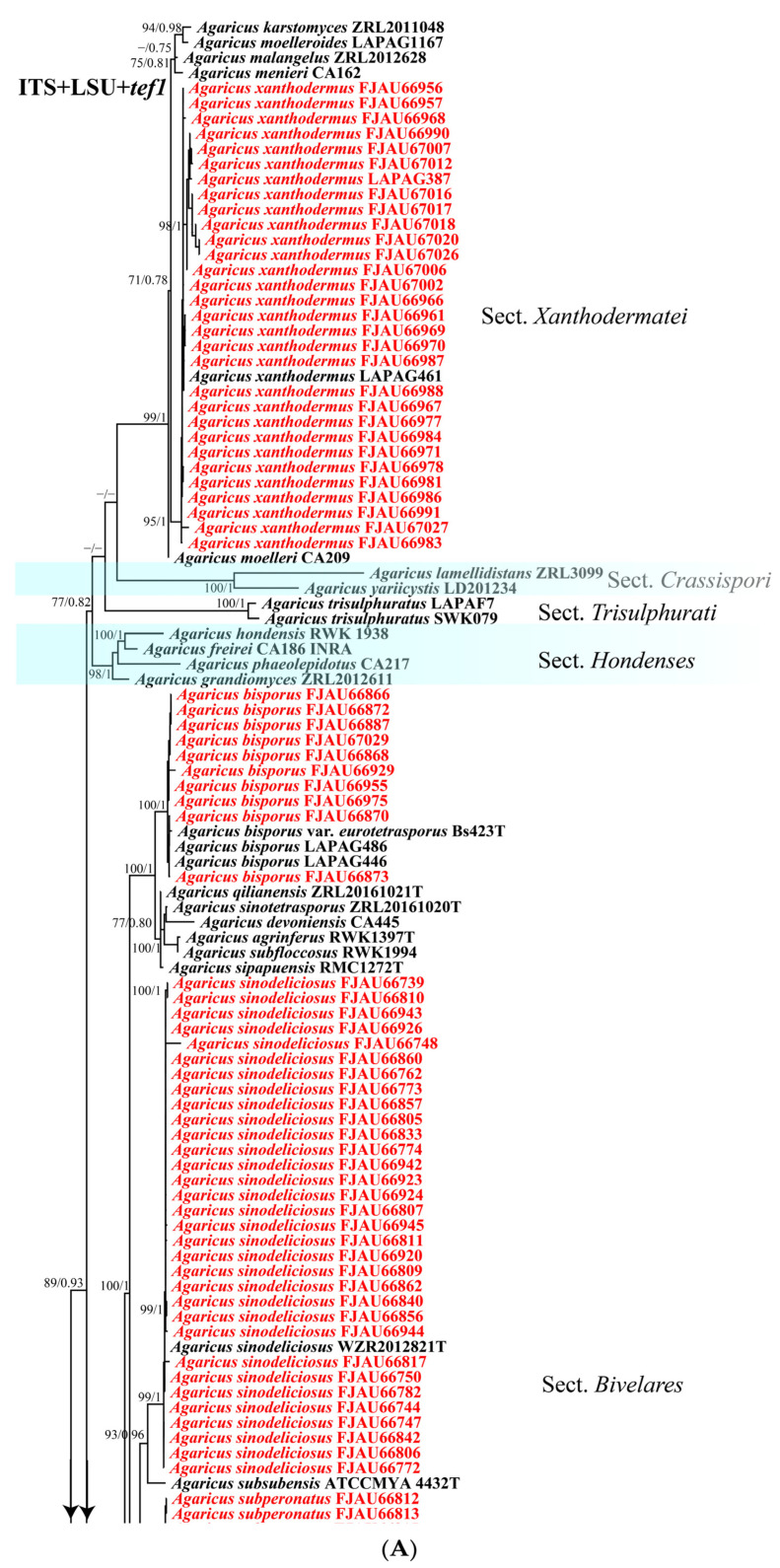

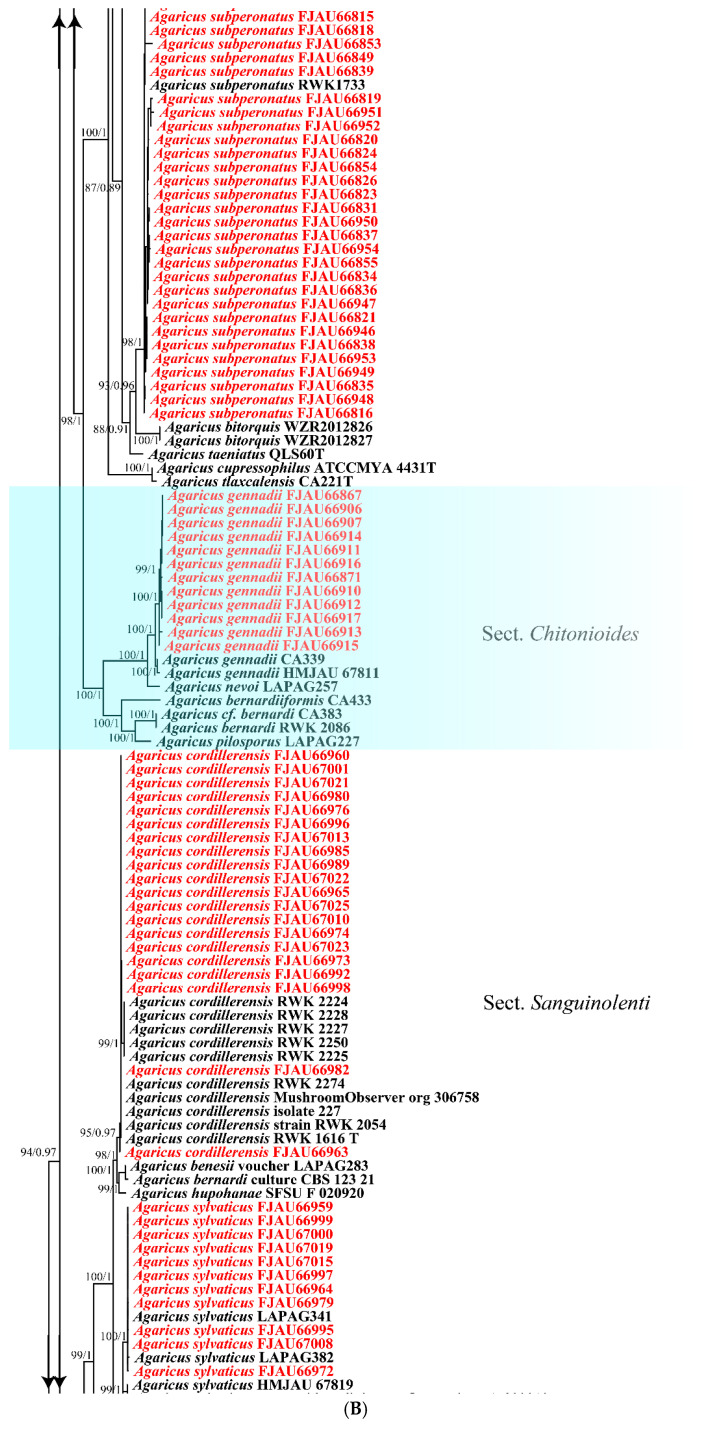

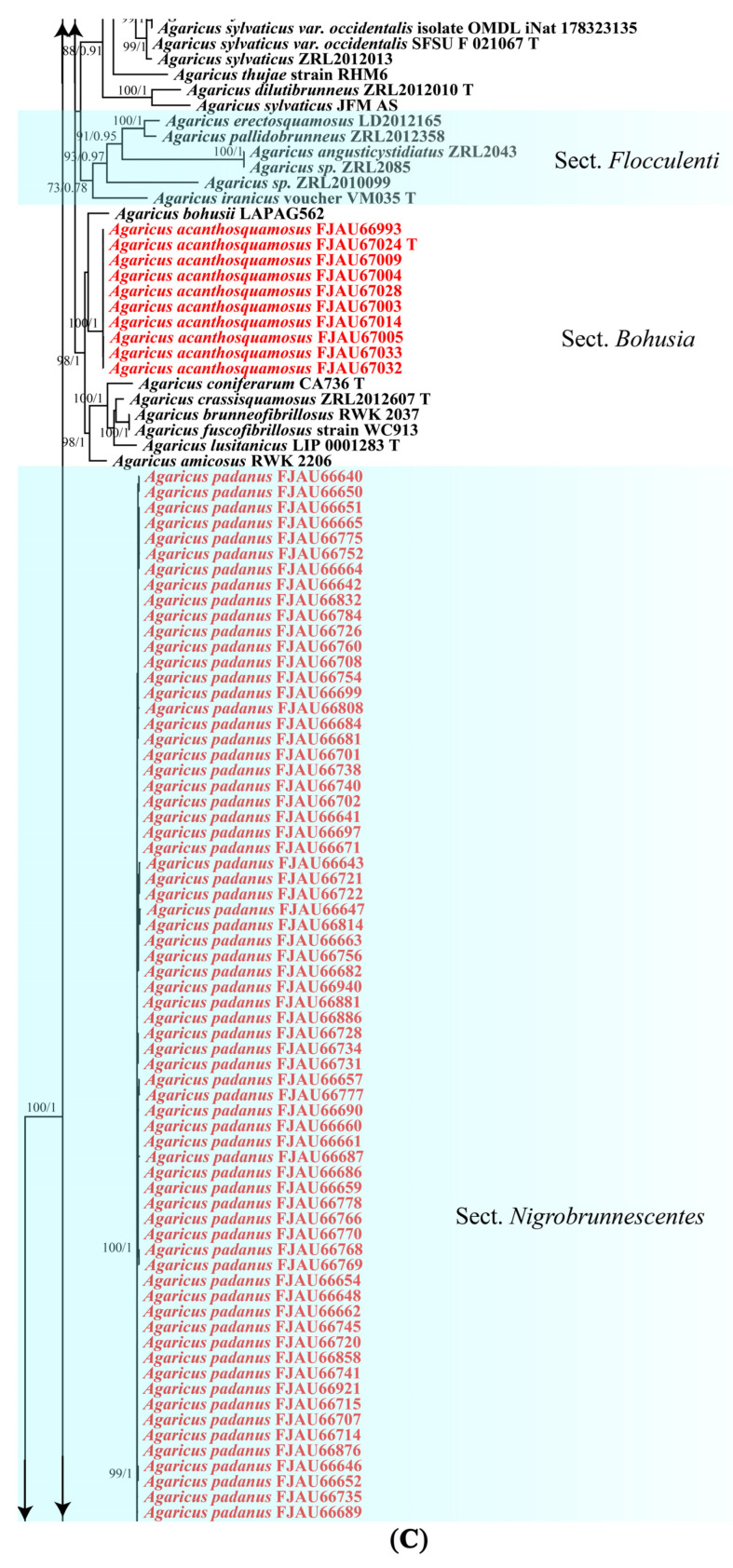

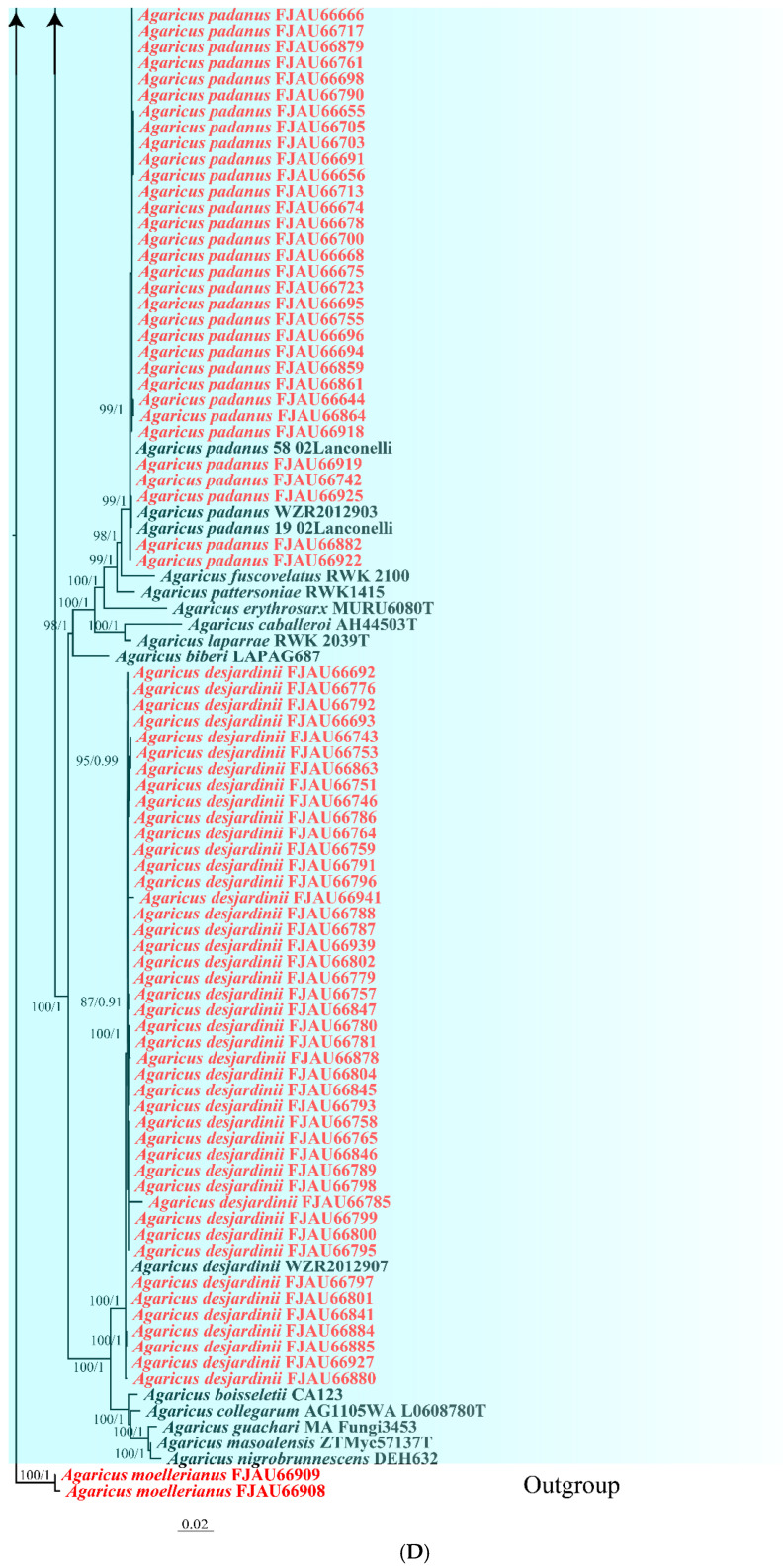
(A) Phylogenetic relationships within *Agaricus* subgen. *Pseudochitonia* inferred from the combined ITS + LSU + *tef1* dataset. The topology shown is the Bayesian consensus tree; Maximum Likelihood bootstrap support (MLBS) and Bayesian posterior probabilities (BPPs) are indicated at nodes as MLBS/BPP. Values of MLBS ≥ 70 and BPP ≥ 0.95 are shown. Taxa newly sequenced in this study are indicated in bold red. T in collection data denotes the type specimen. Owing to its size, the tree is presented in four panels: (**A**–**D**).

The topologies recovered by Maximum Likelihood and Bayesian Inference were largely congruent; the Bayesian consensus tree annotated with ML bootstrap support values (MLBS) and Bayesian posterior probabilities (BPPs) is presented in Figure 1. The Xinjiang collections were assigned to six sections of subgen. *Pseudochitonia*: sect. *Bohusia*, sect. *Bivelares*, sect. *Chitonioides*, sect. *Nigrobrunnescentes*, sect. *Sanguinolenti*, and sect. *Xanthodermatei*. Specifically, sect. *Xanthodermatei* was represented by *A. xanthodermus*; sect. *Bivelares* by *A. bisporus*, *A. sinodeliciosus*, and *A. subperonatus*; sect. *Chitonioides* by *A. gennadii*; sect. *Sanguinolenti* by *A. cordillerensis* and *A. sylvaticus*; sect. *Bohusia* by *A. acanthosquamosus*; and sect. *Nigrobrunnescentes* by *A. padanus* and *A. desjardinii*. The newly described *A. acanthosquamosus* formed a strongly supported monophyletic lineage sister to *A. bohusii* (MLBS/BPP = 100/1; Figure 1C).

### 3.2. Pairwise Genetic Distances

Pairwise p-distances varied among loci and species pairs (Table 3). *A. acanthosquamosus* showed no ITS variation among sampled collections (mean intraspecific distance = 0.0000), whereas mean intraspecific divergences were 0.0013 in LSU and 0.0010 in *tef1*. Mean interspecific distances between *A. acanthosquamosus* and its sister taxon *A. bohusii* were 0.0209 in ITS, 0.0046 in LSU, and 0.0282 in *tef1*.

For *A. cordillerensis* and *A. sylvaticus*, mean interspecific distances were 0.0144 (ITS), 0.0035 (LSU), and 0.0270 (*tef1*). For *A. gennadii* and *A. nevoi*, the corresponding values were 0.0184 (ITS), 0.0048 (LSU), and 0.0257 (*tef1*). The comparison between *A. subperonatus* and *A. sinodeliciosus* yielded a lower mean divergence in LSU (0.0057) than in *tef1* (0.0691). Within-species divergence varied among species and loci; the highest maximum values were recorded in *tef1* for A. gennadii, *A. sylvaticus*, *A. padanus*, and *A. desjardinii*, and in LSU for *A. sinodeliciosus*, *A. subperonatus*, and *A. bisporus* (Table 3).

### 3.3. Habitat-Associated Distribution and Morphological Patterning

The ten Xinjiang species were assigned to three broad habitat categories: montane forest, desert–wetland/sandy habitat, and transitional riparian or disturbed habitat (Table 4). The montane forest assemblage comprised *A. acanthosquamosus*, *A. xanthodermus*, *A. cordillerensis*, and *A. sylvaticus*, all collected predominantly in *P. schrenkiana* forests or adjacent litter-rich soils and all fruiting epigously. The desert–wetland assemblage comprised *A. sinodeliciosus*, *A. padanus*, and *A. desjardinii*, recorded from the Ebinur Lake region and associated sandy or wetland-margin habitats, with fruiting habit ranging from semi-hypogeous to hypogeous. *A. bisporus*, *A. subperonatus*, and *A. gennadii* were collected from riparian woodland, wetland margins, enriched soils, or disturbed ground and were assigned to the transitional category (Table 4).

**Figure 2:**
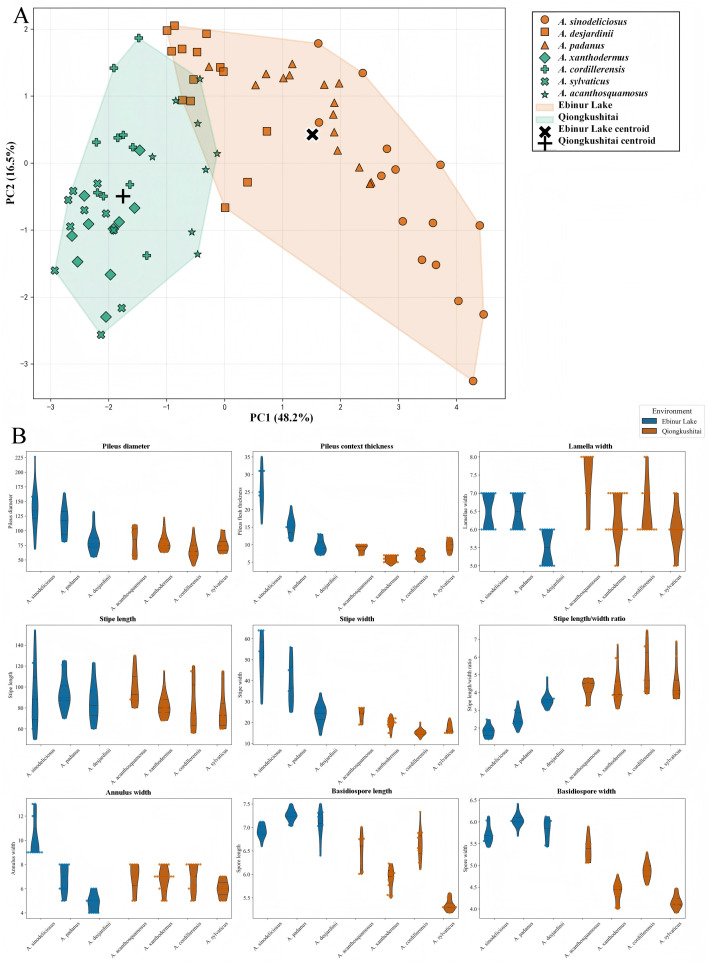
Morphological differentiation between the Ebinur Lake desert–wetland assemblage and the Qiongkushitai montane forest assemblage. (**A**) Principal component analysis based on nine traits (eight primary measurements and stipe length/width ratio), with collection-level means as the unit of observation. Convex hulls enclose the two assemblages. (**B**) Collection-level distributions of the same nine traits presented as violin plots; points represent individual collection means. Blue: Ebinur Lake assemblage (*A. sinodeliciosus*, *A. padanus*, *A. desjardinii*). Orange: Qiongkushitai assemblage (*A. acanthosquamosus*, *A. xanthodermus*, *A. cordillerensis*, *A. sylvaticus*).

Principal component analysis based on nine traits (eight primary measurements plus stipe length/width ratio) showed partial separation between the Ebinur Lake and Qiongkushitai assemblages in morphological space (Figure 2A). The first two principal components together accounted for 64.7% of the total variation (PC1: 48.2%; PC2: 16.5%). The Ebinur Lake assemblage was distributed predominantly toward positive PC1 values, whereas the Qiongkushitai assemblage was concentrated toward negative PC1 values. The convex hulls of the two assemblages partly overlapped, but their centroids were separated.

PERMANOVA based on Euclidean distances calculated from the standardized collection-level morphological trait matrix revealed significant multivariate differentiation between the Ebinur Lake and Qiongkushitai assemblages (pseudo-F = 51.83, *p* = 0.0001; Table 5). Assemblage membership explained 34.6% of the total morphological variation (*R*^2^ = 0.346), indicating moderate but significant assemblage-level structuring of morphological traits.

Kruskal–Wallis tests detected significant differences between the two assemblages in six of the nine traits (Table 6). The Ebinur Lake assemblage had a larger mean pileus diameter (112.39 mm vs. 76.63 mm; *H* = 22.42, *p* = 2.20 × 10^−6^), thicker pileus context (16.95 mm vs. 7.66 mm; *H* = 41.74, *p* = 1.04 × 10^−10^), wider stipes (37.41 mm vs. 18.66 mm; *H* = 39.85, *p* = 2.74 × 10^−10^), longer basidiospores (7.07 μm vs. 6.10 μm; *H* = 42.77, *p* = 6.17 × 10^−11^), and wider basidiospores (5.86 μm vs. 4.65 μm; *H* = 54.60, *p* = 1.47 × 10^−13^) than the Qiongkushitai assemblage. The stipe length/width ratio was higher in the Qiongkushitai assemblage than in the Ebinur Lake assemblage (4.89 vs. 2.75; *H* = 43.65, *p* = 3.92 × 10^−11^). Lamella width (*H* = 0.82, *p* = 0.366), stipe length (*H* = 0.13, *p* = 0.720), and annulus width (*H* = 0.71, *p* = 0.401) did not differ significantly between the two assemblages.

Species-level violin plots confirmed these assembly-level patterns (Figure 2B). The three Ebinur Lake species—*A. sinodeliciosus, A. padanus*, and *A. desjardinii*—showed trait distributions shifted toward larger values in pileus diameter, pileus context thickness, stipe width, and basidiospore dimensions relative to the four Qiongkushitai species—*A. acanthosquamosus*, *A. xanthodermus*, *A. cordillerensis*, and *A. sylvaticus*. The stipe length/width ratio showed the inverse pattern, with lower mean values in the Ebinur Lake assemblage and higher mean values in the Qiongkushitai assemblage. Stipe length showed broad overlap between the two assemblages.

**Table 6:** Kruskal–Wallis comparisons of nine morphological and derived traits between the Ebinur Lake desert–wetland assemblage and the Qiongkushitai montane forest assemblage.

Trait	*H* Value	*p* Value	Significance	Mean in Ebinur Lake Assemblage	Mean in Qiongkushitai Assemblage
Pileus diameter (mm)	22.42	2.20 × 10^−6^	***	112.39	76.63
Pileus context thickness (mm)	41.74	1.04 × 10^−10^	***	16.95	7.66
Lamella width (mm)	0.82	0.366	ns	5.91	5.63
Stipe length (mm)	0.13	0.720	ns	90.89	88.82
Stipe width (mm)	39.85	2.74 × 10^−10^	***	37.41	18.66
Stipe length/width ratio	43.65	3.92 × 10^−11^	***	2.75	4.89
Annulus width (mm)	0.71	0.401	ns	6.77	5.71
Basidiospore length (μm)	42.77	6.17 × 10^−11^	***	7.07	6.10
Basidiospore width (μm)	54.60	1.47 × 10^−13^	***	5.86	4.65

H values and *p* values are based on Kruskal–Wallis tests. Mean values are given for each habitat assemblage. Significance levels: ns, not significant; ***, *p* < 0.001.

### 3.4. Key to Species of Agaricus subgen. Pseudochitonia from Xinjiang

1. Context yellowing when bruised or cut; KOH reaction positive, yellow .................................................................................................. *A. xanthodermus*

1. Context not yellowing when bruised or cut; KOH reaction negative ........................................................................................................................ 2

2. Schäffer’s reaction positive....................................................................................... 3

2. Schäffer’s reaction negative ..................................................................................... 4

3. Pileus with squarrose spinose scales; annulus single-layered ............................................................................. *A. acanthosquamosus*

3. Pileus without squarrose spinose scales; annulus double- to pseudo-triple-layered ................................................................................... *A. gennadii*

4. Basidia predominantly 2-sterigmata; basidiospores 6–7.5 × 5–6 μm .......*A. bisporus*

4. Basidia predominantly 4-sterigmata..........................................................................5

5. Pleurocystidia present.............................................................................................. 6

5. Pleurocystidia absent ............................................................................................... 7

6. Pileus surface ochraceous, with dark brown scales; annulus ascending.................. *A. sinodeliciosus*

6. Pileus surface white to dirty white, with brown scales; annulus descending............................................................................................ *A. subperonatus*

7. Annulus inferior, double-layered, ascending.......................................... *A. bitorquis*

7. Annulus not inferior.................................................................................................. 8

8. Basidiomata semi-hypogeous to hypogeous at maturity .......................................... 9

8. Basidiomata epigeous ............................................................................................ 10

9. Pileus and stipe with large, recurved squamules ..................................*A. desjardinii*

9. Pileus with appressed, angular scales ........................................................*A. padanus*

10. Pileus scales fibrillose; basidiospores mostly ellipsoid to elongate–ellipsoid, avQ = 1.58.........................................................................................................*A. cordillerensis*

10. Pileus scales angular to fibrillose; basidiospores mostly subglobose to broadly ellipsoid, avQ = 1.31 ................................................................................. *A. sylvaticus*

### 3.5. Taxonomy

***Agaricus*** **subgen.** ***Pseudochitonia*** **sect.** ***Bohusia.***

***Agaricus acanthosquamosus*** **Z.X. Qi, B. Zhang & Y. Li, sp. nov.**

**MycoBank No:** 863651.

**Figure 3:**
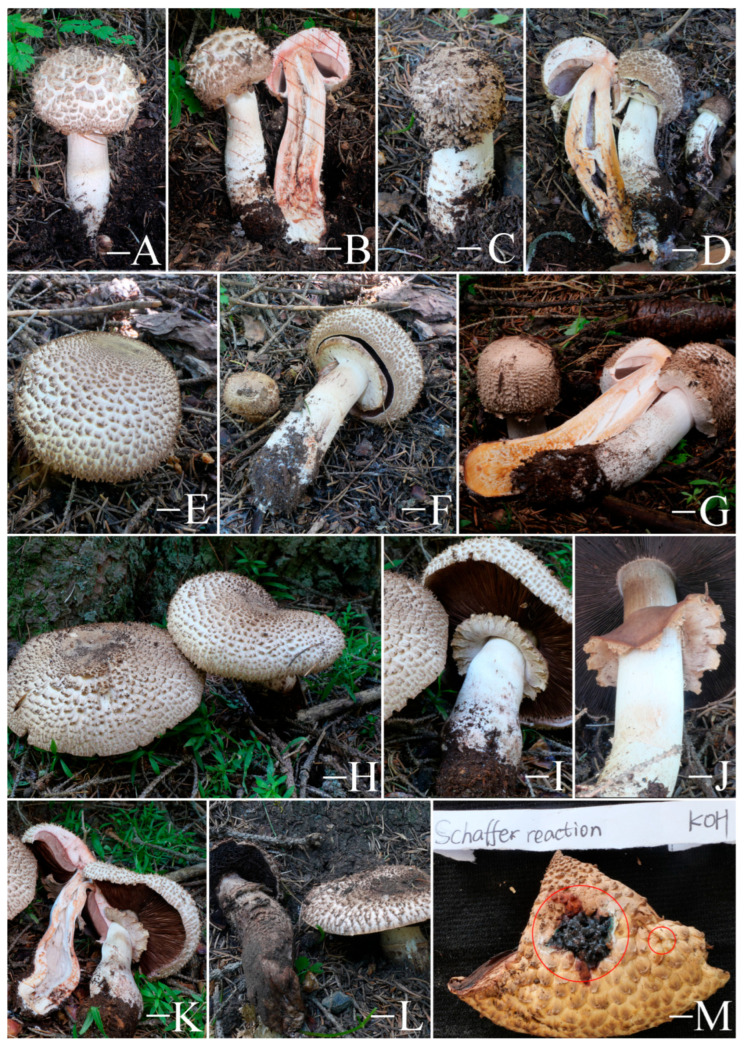
*Agaricus acanthosquamosus* in situ. (**A**–**G**): Young basidiomata. (**H**–**K**): Mature basidiomata. (**L**): Overmature basidiomata. (**M**): Chemical reactions (left: Schäffer’s reaction; right: KOH reaction). (**A**–**M**): Photographs by Qi ZX. Scale bars: (**A**–**M**) = 10 mm.

**Figure 4:**
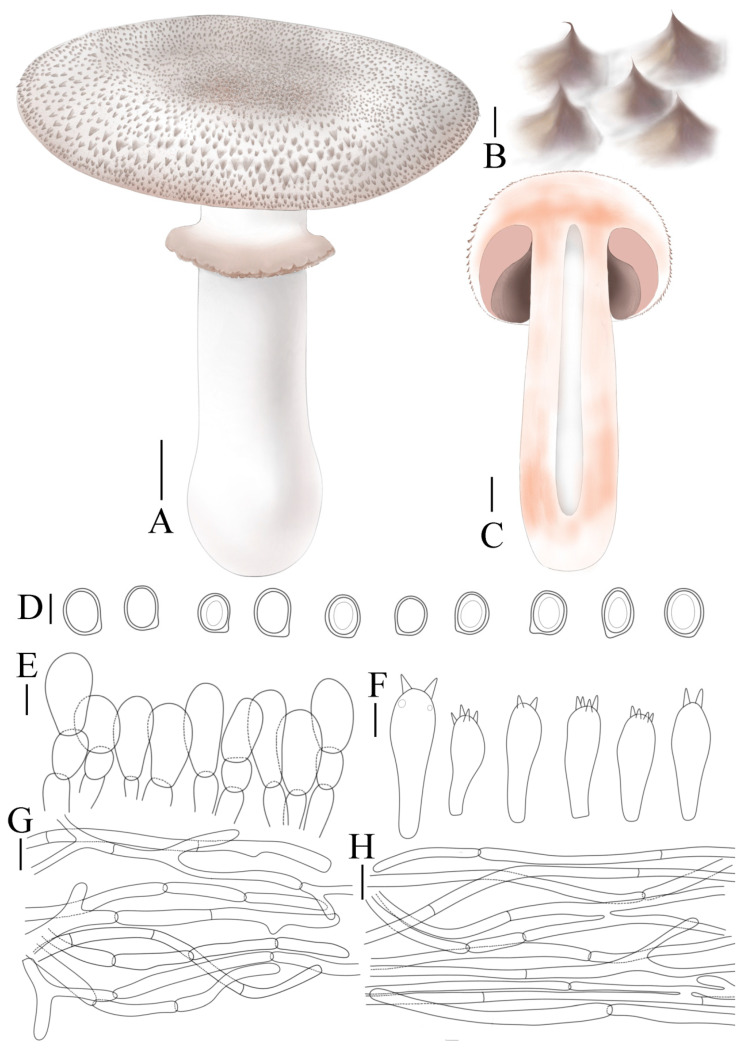
Macro- and micromorphology of *Agaricus acanthosquamosus*. (**A**): Basidiomata. (**B**): Close-up of scales. (**C**): Longitudinal section. (**D**): Basidiospores. (**E**): Cheilocystidia. (**F**): Basidia. (**G**): Pileipellis hyphae. (**H**): Annulus hyphae. Scale bars: (**A**–**C**) = 10 mm, (**D**) = 5 μm, (**E**–**H**) = 10 μm.

**Figure 23:**
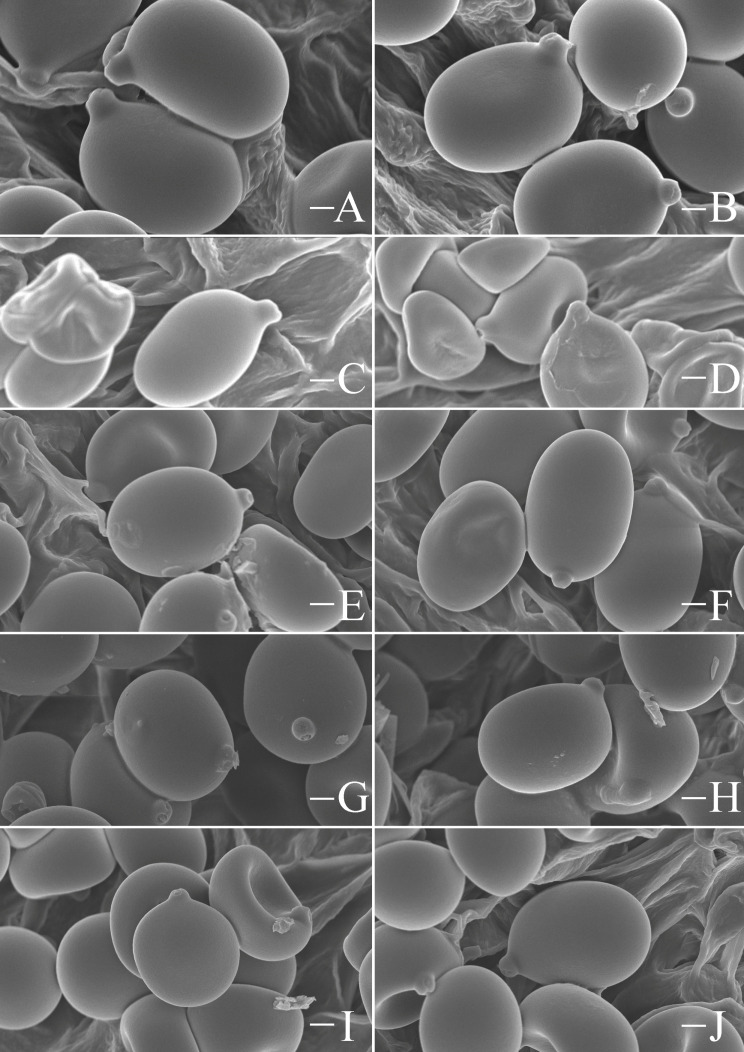
Scanning electron micrographs of basidiospores. (**A**,**B**): *Agaricus acanthosquamosus*; (**C**,**D**): *A. xanthodermus*; (**E**,**F**): *A. bisporus*; (**G**,**H**): *Agaricus sinodeliciosus*; (**I**,**J**): *A. subperonatus*. Scale bars = 1 μm.

Figure 3 and Figure 4, Figure 23A,B.

**Etymology.** The specific epithet acanthosquamosus is derived from the Greek acantha, spine or thorn, and the Latin squamosus, scaly, referring to the conspicuous squarrose spinose scales on the pileus surface.

**Diagnosis.** *Agaricus acanthosquamosus* is morphologically close to *A. bohusii*, but differs in its spinose squarrose pileus scales, subglobose to broadly ellipsoid basidiospores, a solitary to scattered fruiting habit, and context initially orange-yellow, then bright red to blood red, finally reddish brown when cut or bruised. By contrast, *A. bohusii* has appressed pileus scales, ellipsoid basidiospores, fasciculate basidiomata, and context lacking a distinct color change.

**Holotype.** CHINA. Xinjiang Uygur Autonomous Region, Tekes County, Qiongkushitai, in *P. schrenkiana* forest, on humus-rich soil, 43.9315654° N, 82.2380412° E, 2555 m elev., 31 August 2024, Z.X. Qi, FJAU67024. GenBank accessions: ITS PZ101656, LSU PZ106830, *tef1* PZ137692.

Description. Basidiomata medium to large. Pileus 47–156 mm diam., young basidioma hemispherical to campanulate, white (1A1), bearing large, flaky, brownish (7F5–7), recurved scales; margin distinctly incurved; at maturity, plano-convex to centrally depressed, surface with rigid, dark brown (8F7–8), spinose squarrose scales 5–15 mm long with acute apices, margin slightly incurved. Pileus surface staining blood red to deep red (9A6–9E6) when bruised. Lamellae free, crowded, with lamellulae, 3–6 mm wide; edge entire; initially white to pale pink (1A1–11A2), becoming dark brown (8F8) at maturity. Stipe 36–95 × 11–28 mm, cylindrical, sometimes unequal, distinctly hollow; base obtuse; above annulus smooth, white (1A1); below annulus smooth when young, later covered with thick, annular, brown (8F5–8), recurved scales, bruising reddish brown (9A8). Annulus in the upper-middle part of stipe, descending, single-layered annulus with differentiated upper and lower surfaces, membranous, with appressed layers; upper layer circular, brown (8E5), upper surface smooth, 3–6 mm wide, margin smooth; lower surface covered with dirty white to pale brown (1A1–6D5), coarse, angular squamules. Context 5–9 mm thick, firm, white, first turning orange-yellow (5B5–8) when cut, then bright red to blood red (9A6–8), finally reddish brown (8E8). Odor indistinct. Spore print brown. KOH reaction negative, Schäffer’s reaction positive, violaceous (15C6).

Basidiospores [500/15/10] 4.5–7.0 × 4.5–6.0 μm, avL × avW = 6.01 × 5.06 μm, Q = 1.11–1.41, avQ = 1.18, mostly subglobose to broadly ellipsoid, rarely ellipsoid, brownish, wall 0.5–1 μm thick, apiculus minute. SEM: basidiospores smooth, unornamented, subglobose to ellipsoid in outline, with a small but distinct apiculus. Basidia 18–35 × 8–11 μm, clavate, smooth, hyaline, with irregular contents; 4(2)-sterigmata, 2–3 μm long. Cheilocystidia abundant, clustered, 13–35 × 8–13 μm, short clavate, vesiculose to pyriform, thin-walled, smooth, hyaline to pale brown, base with 1–2 septa. Pleurocystidia not observed. Pileipellis a cutis composed of cylindrical hyphae 4–10 μm wide, smooth, hyaline or with yellowish-brown intracellular pigment, branched, slightly constricted at septa, terminal elements obtuse, 6–11 μm wide. Hyphae of upper and lower annuli similar, cylindrical, hyaline, 5–10 μm wide, frequently branched, slightly constricted at septa, terminal elements obtuse, 6–9 μm wide. Clamp connections absent.

Habitat: Solitary or scattered on humus-rich soil in clearings of *P. schrenkiana* forests, fruiting from July to September.

Distribution in China: Xinjiang.

Global distribution: China.

Specimens examined. CHINA. Xinjiang Uygur Autonomous Region: Urumqi, Xibaiyanggou Scenic Area, 2523 m elev., 9 August 2021, Qi ZX FJAU67028; Ili Kazakh Autonomous Prefecture, Tekes County, Qiongkushitai, 2633 m elev., 7 July 2024, Qi ZX FJAU66993; Ili Kazakh Autonomous Prefecture, Tekes County, Qiongkushitai, 2565 m elev., 15 July 2024, Qi ZX FJAU67003; Ili Kazakh Autonomous Prefecture, Tekes County, Qiongkushitai, 2510 m elev., 23 July 2024, Qi ZX FJAU67004, FJAU67005; Ili Kazakh Autonomous Prefecture, Tekes County, Qiongkushitai, 2674 m elev., 3 August 2024, Qi ZX FJAU67009, FJAU67032, FJAU67033; and Ili Kazakh Autonomous Prefecture, Tekes County, Qiongkushitai, 2516 m elev., 20 August 2024, Qi ZX FJAU67014.

Notes. *A. acanthosquamosus* is characterized by its spinose squarrose pileus scales, context turning orange-yellow and then reddish brown when cut or bruised, hollow stipe. Intraspecific mean p-distances were 0.0000 for ITS, 0.0013 for LSU, and 0.0010 for *tef1*.

This species shares morphological similarities with *A. amicosus* and *A. bohusii*, but detailed comparative analyses reveal several distinguishing features. *A. acanthosquamosus* is differentiated from *A. amicosus* by its squarrose spinose pileus scales versus appressed scales in the latter [8]. The distinction from *A. bohusii* is more comprehensive: *A. acanthosquamosus* exhibits squarrose spinose scales, subglobose to broadly ellipsoid basidiospores (Q = 1.11–1.41), and solitary to scattered basidiomata. In contrast, *A. bohusii* possesses appressed scales, ellipsoid basidiospores (Q = 1.32–1.53), and fasciculate (clustered) basidiomata [13,16]. These distinctions are supported by genetic divergence. The interspecific p-distances between *A. acanthosquamosus* and *A. bohusii* are 0.0209 for ITS, 0.0046 for LSU, and 0.0282 for *tef1*. It is also separated from *A. amicosus* by an ITS p-distance of 0.0202.

Phylogenetically, our analyses demonstrate that the Xinjiang specimens form a well-supported monophyletic branch (MLBS/BPP = 100/1, Figure 1C) sister to *A. bohusii*.

***Agaricus*** **subgen.** ***Pseudochitonia*** **sect.** ***Xanthodermatei.***

***Agaricus xanthodermus*** **Genev., Bull. Soc. bot. Fr. 23: 31 (1876).**

**Figure 5:**
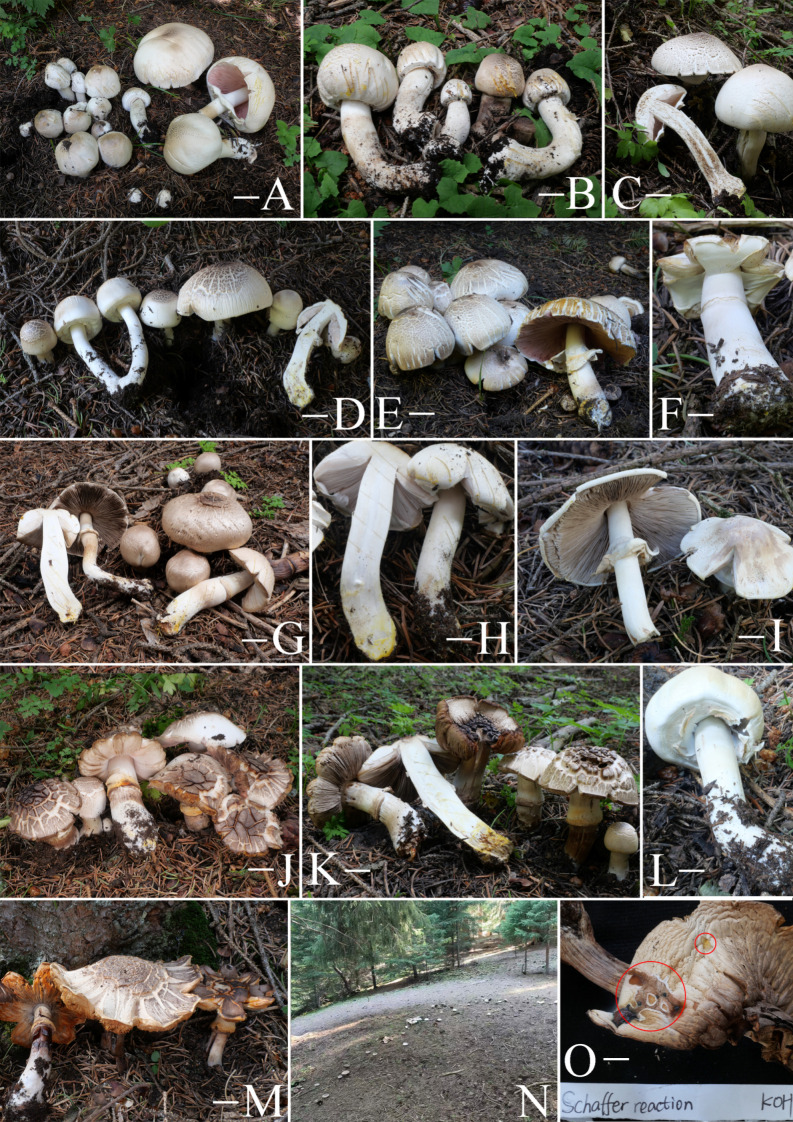
*Agaricus xanthodermus* in situ. (**A**–**I**): Morphological features under normal environmental conditions. (**J**–**M**): Morphological features under extreme environmental conditions. (**N**): Fairy ring formation. (**O**): Chemical reactions (left: Schäffer’s reaction; right: KOH reaction). (**A**–**O**): Photographs by Qi ZX. Scale bars: (**A**–**M**,**O**) = 10 mm.

**Figure 6:**
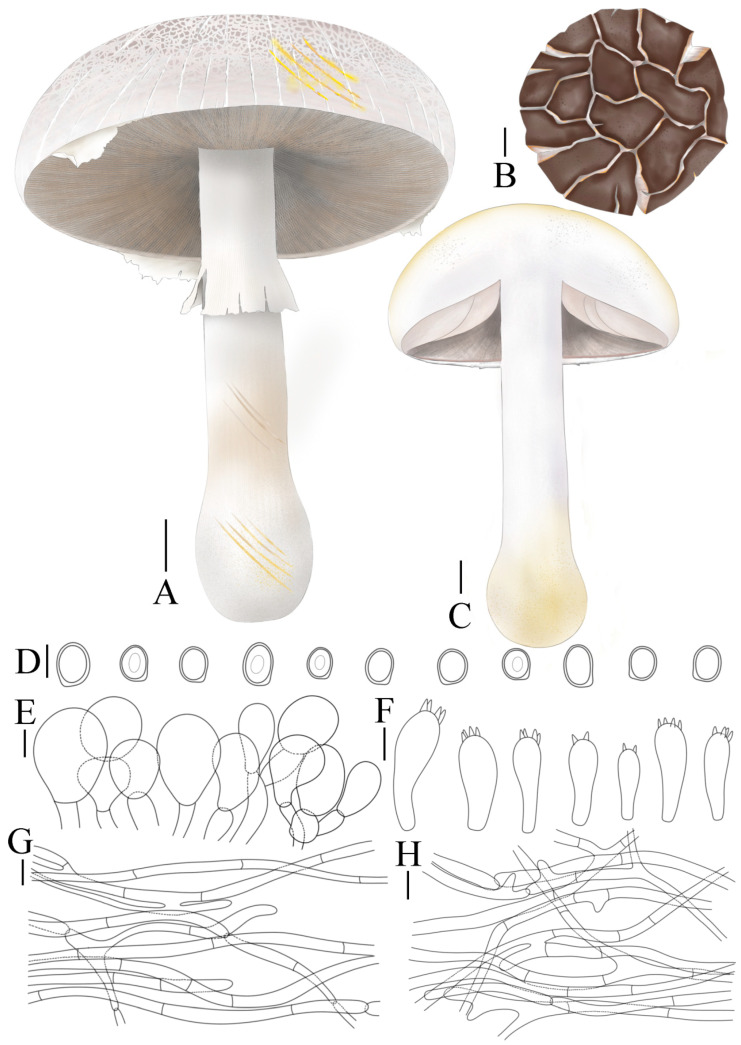
Macro- and micromorphology of *Agaricus xanthodermus*. (**A**): Basidiomata. (**B**): Close-up of pileus. (**C**): Longitudinal section. (**D**): Basidiospores. (**E**): Cheilocystidia. (**F**): Basidia. (**G**): Pileipellis hyphae. (**H**): Annulus hyphae. Scale bars: (**A**–**C**) = 10 mm, (**D**) = 5 μm, (**E**–**H**) = 10 μm.

Figure 5 and Figure 6, Figure 23C,D.

Description. Basidiomata small to large. Pileus 30–170 mm diam., initially subglobose, hemispherical to campanulate, white (1A1), smooth, with mouse-gray (3C1), pale brown to dark brown (6E6–6F8) shallow scales at center; margin entire, sometimes with white veil remnants; at maturity, flattened hemispherical, convex to plano-convex, with grayish brown (5D1) scales splitting into flaky dark brown (6F8) scales at center and smaller, block-like, pale brown (6E6) scales toward margin, margin sometimes yellowish brown (3B4). In overmature or sun-exposed basidiomata, pileus dry and cracked, with silver-gray (1D1), reddish brown to dark brown (8A6–8F8), radial or irregular patches, margin often reflexed and split, exposing white to orange-red (1A1–6A7) context. Pileus surface rapidly staining bright to vivid yellow (1A5–8) when scratched or bruised, especially at the margin. Lamellae free, crowded, unequal, 3–6 mm wide, initially white to dirty white, then pink, becoming reddish brown to dark brown at maturity; in sun-exposed specimens, sometimes orange-red, with lamellae partially appressed. Stipe 20–150 × 3–30 mm, clavate, sometimes slightly curved, initially solid, hollow at maturity; base slightly bulbous to 33 mm wide; above annulus white, smooth; below annulus white to pale brown, slightly yellowing when scratched or bruised. Annulus median, simple, membranous, soft, 1–3 mm thick, 2–9 mm wide, descending; upper surface smooth; lower surface squamulose, white to grayish white. In dried specimens, annulus thickened, pendent, appressed to stipe, bright yellowish brown (6A6). Context white, 3–7 mm thick, pileus context pale yellow in section, stipe context vivid to bright yellow, especially at base. Odor faint, but crushed stipe base with distinct phenolic smell. Dried basidiomata dark yellowish brown, pileus thin and fragile. Spore print dark brown. KOH reaction positive, yellow (5B7), Schäffer’s reaction negative.

Basidiospores [500/78/34] (5–)5.5–6(–7) × (4–)4.5–4.7(–5) μm, avL × avW = 6.23 × 4.63 μm, Q = 1.11–1.55, avQ = 1.35, mostly broadly ellipsoid to ellipsoid, rarely subglobose, brownish, wall 0.4–0.9 μm thick, apiculus minute. SEM: basidiospores smooth, unornamented, broadly ellipsoid to ellipsoid in outline, with a minute apiculus. Basidia 17–25(–33) × 7–10(–11) μm, clavate to broadly clavate, hyaline to pale brown; 4(2)-sterigmata, 2–4 μm long. Cheilocystidia abundant, clustered, (13–)20–30 × (5–)7–18 μm, clavate, pyriform, bulbous, subglobose to ellipsoid, thin-walled, hyaline, base with 1–2 septa. Pleurocystidia absent. Pileipellis a cutis composed of branched, thin-walled hyphae 4–7 μm wide, with obtuse terminal elements 5–8 μm wide, hyaline to pale brown with intracellular pigment. Annular hyphae cylindrical, hyaline to pale yellowish pigmented, 6–8 μm wide, frequently branched and interwoven, slightly constricted at septa, terminal elements obtuse, 6–10 μm wide. Clamp connections absent.

Habitat: Scattered to gregarious on relatively dry litter under or near *P. schrenkiana* forests, and at edges of grazing areas, sometimes forming arcs or fairy rings, fruiting from early June to early September.

Distribution in China: Hebei, Qinghai, Gansu, Tibet, Shanxi, and Xinjiang [13,46].

Global distribution: France [47], Ethiopia [48], USA [10,49], Sweden [50], and China [13,46].

Specimens examined. CHINA. Xinjiang Uygur Autonomous Region: Ili Kazakh Autonomous Prefecture, Tekes County, Qiongkushitai, 2154 m elev., 10 July 2023, Qi ZX FJAU66983, FJAU66984; Ili Kazakh Autonomous Prefecture, Tekes County, Qiongkushitai, 1764 m elev., 11 July 2023, Qi ZX FJAU66956, FJAU66957; Ili Kazakh Autonomous Prefecture, Tekes County, Qiongkushitai, 2294 m elev., 13 July 2023, Qi ZX FJAU66961; Ili Kazakh Autonomous Prefecture, Tekes County, Qiongkushitai, 2299 m elev., 19 July 2023, Qi ZX FJAU66966, FJAU66967, FJAU66968, FJAU66969; Ili Kazakh Autonomous Prefecture, Tekes County, Qiongkushitai, 2337 m elev., 28 July 2023, Qi ZX FJAU66970, FJAU66971; Ili Kazakh Autonomous Prefecture, Tekes County, Qiongkushitai, 2372 m elev., 25 August 2023, Qi ZX FJAU66977; Ili Kazakh Autonomous Prefecture, Tekes County, Qiongkushitai, 2362 m elev., 27 August 2023, Qi ZX FJAU66978; Ili Kazakh Autonomous Prefecture, Tekes County, Qiongkushitai, 2248 m elev., 31 August 2023, Qi ZX FJAU66981; Ili Kazakh Autonomous Prefecture, Tekes County, Qiongkushitai, 2375 m elev., 16 June 2024, Qi ZX FJAU66986; Ili Kazakh Autonomous Prefecture, Tekes County, Qiongkushitai, 2312 m elev., 20 June 2024, Qi ZX FJAU66987; Ili Kazakh Autonomous Prefecture, Tekes County, Qiongkushitai, 2360 m elev., 23 June 2024, Qi ZX FJAU66988; Ili Kazakh Autonomous Prefecture, Tekes County, Qiongkushitai, 2176 m elev., 30 June 2024, Qi ZX FJAU66990; Ili Kazakh Autonomous Prefecture, Tekes County, Qiongkushitai, 2378 m elev., 4 July 2024, Qi ZX FJAU66991; Ili Kazakh Autonomous Prefecture, Tekes County, Qiongkushitai, 2215 m elev., 14 July 2024, Qi ZX FJAU67002; Ili Kazakh Autonomous Prefecture, Tekes County, Qiongkushitai, 2199 m elev., 24 July 2024, Qi ZX FJAU67006, FJAU67007; Ili Kazakh Autonomous Prefecture, Tekes County, Qiongkushitai, 2362 m elev., 4 August 2024, Qi ZX FJAU67012; Ili Kazakh Autonomous Prefecture, Tekes County, Qiongkushitai, 2304 m elev., 22 August 2024, Qi ZX FJAU67016; Ili Kazakh Autonomous Prefecture, Tekes County, Qiongkushitai, 2100 m elev., 24 August 2024, Qi ZX FJAU67017, FJAU67018; Ili Kazakh Autonomous Prefecture, Tekes County, Qiongkushitai, 2256 m elev., 27 August 2024, Qi ZX FJAU67020; Ili Kazakh Autonomous Prefecture, Tekes County, Qiongkushitai, 2377 m elev., 6 September 2024, Qi ZX FJAU67026; and Ili Kazakh Autonomous Prefecture, Tekes County, Qiongkushitai, 2254 m elev., 9 September 2024, Qi ZX FJAU67027.

Notes: The Xinjiang collections are assigned to *A. xanthodermus* based on the rapid yellowing of the pileus margin and stipe base when bruised, bulbous stipe base, phenolic odor, and positive KOH reaction. Intraspecific mean p-distances were 0.0001 for ITS, 0.0041 for LSU, and 0.0091 for *tef1.*

Under exposed, dry, high-altitude conditions, the Xinjiang collections showed pronounced phenotypic variation, including cracked pileus, thickened and appressed annuli, and orange-red tones in the lamellae [16,51]. These features are interpreted as environmentally induced variation. *A. xanthodermus* resembles *A. moelleri*, but differs in its smoother pileus surface or easily removable gray-brown fibrils, whereas *A. moelleri* usually has more persistent fibrillose squamules [52]. The predominance of broadly ellipsoid basidiospores also helps distinguish *A. xanthodermus* from *A. moelleri*, which more often has ellipsoid to elongate ellipsoid spores. The ITS p-distance between the two species was 0.0048. *A. xanthodermus* further differs from *A. californicus* in its more rapid and intense yellowing reaction and stronger phenolic odor [14].

***Agaricus*** **subgen.** ***Pseudochitonia*** **sect.** ***Bivelares.***

***Agaricus bisporus*** **(J.E. Lange) Imbach, Mitt. naturf. Ges. Luzern 15: 15 (1946).**

**Figure 7:**
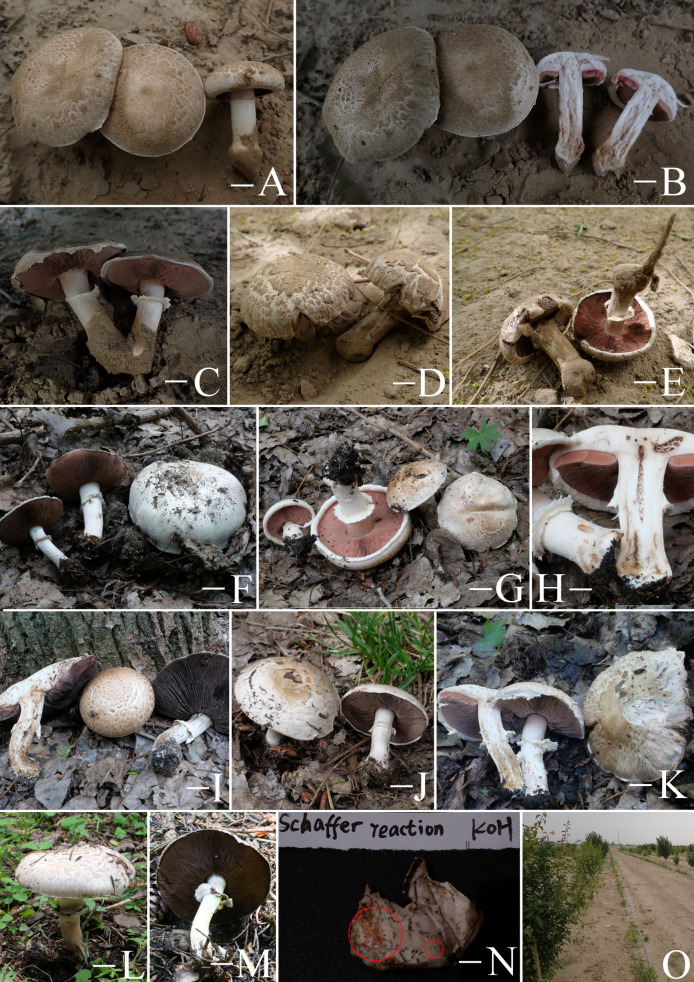
*Agaricus bisporus* in situ. (**A**–**E**): Sandy soil environment. (**F**–**M**): Environment with thick humus layer. (**N**): Chemical reactions (left: Schäffer’s reaction; right: KOH reaction). (**O**): Habitat. (**A**–**E**): Photographs by Zhang B. (**F**–**O**): Photographs by Qi ZX. Scale bars: (**A**–**N**) = 10 mm.

**Figure 8:**
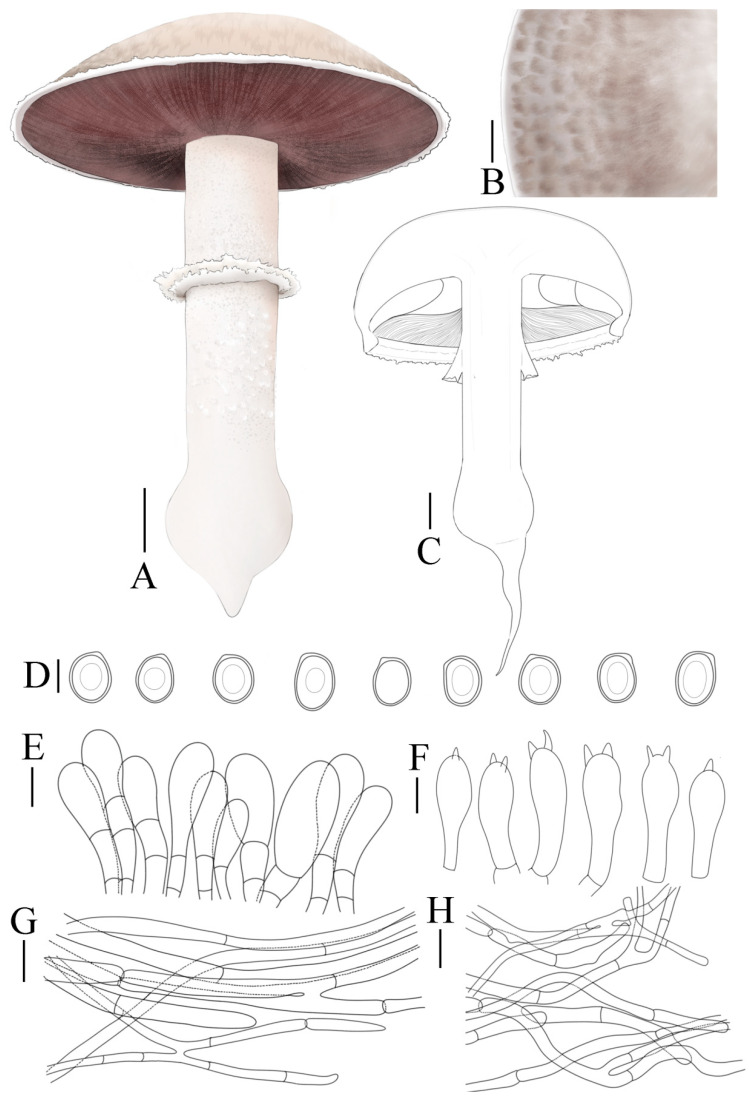
Macro- and micromorphology of *Agaricus bisporus*. (**A**): Basidiomata. (**B**): Close-up of pileus. (**C**): Longitudinal section. (**D**): Basidiospores. (**E**): Cheilocystidia. (**F**): Basidia. (**G**): Pileipellis hyphae. (**H**): Annulus hyphae. Scale bars: (**A**–**C**) = 10 mm, (**D**) = 5 μm, (**E**–**H**) = 10 μm.

Figure 7 and Figure 8, Figure 23E,F.

Description. Basidiomata small to large. Pileus 37–107 mm diam., initially hemispherical to convex, white to dirty white (1A1), smooth or with inconspicuous brown scales; margin incurved with remnants of universal veil; at maturity, plano-convex, dirty white, with brown (6E7), angular, tile-like scales, denser at center and sparser toward margin, without veil remnants. Pileus surface unchanged when bruised. Lamellae free, moderately crowded, unequal, with 2–4 series of lamellulae, 4–5 mm wide; edge smooth; initially white to pale pink, becoming pale brown to dark brown. Stipe 35–87 × 6–19 mm, subcylindrical, solid; base subglobose to 22 mm wide; surface above annulus white, sometimes with pale reddish-brown snakeskin-like striations; below annulus with white to dirty white fibrillose scales. In sandy soil, stipe base with clavate rhizomorphs, 47–71 mm long, 6–11 mm wide. Annulus median, single- to pseudo-double-layered, membranous, persistent, 5–8 mm wide, 2–3 mm thick, descending, broadly attached to stipe; upper surface smooth to slightly striate; lower surface with silky scales, margin undulate to floccose, white. Context white, 7–10 mm thick, fleshy, lamellar context turning pale red when cut, pileus and stipe context otherwise unchanged. Odor faint, mushroom-like. Spore print brown. KOH reaction negative, Schäffer’s reaction negative.

Basidiospores [300/16/10] (6–)6.2–7(–7.5) × 5–5.8(–6) μm, avL × avW = 6.62 × 5.26 μm, Q = (1.09–)1.17–1.36(–1.40), avQ = 1.26, mostly subglobose to broadly ellipsoid, rarely ellipsoid, dark brown, wall thick, apiculus small. SEM: basidiospores smooth, unornamented, subglobose to broadly ellipsoid in outline, with a small apiculus. Basidia (18–)20–26(–27) × (5.5–)6–7.5(–8) μm, clavate, with 2-sterigmata, 2–4 μm long, hyaline or with pale brown pigment. Cheilocystidia abundant, gregarious, (14–)15–26.5(–27) × (7–)7.5–11(–11.5) μm, clavate, hyaline or with pale brown intracellular pigment, base with 1–2 septa. Pleurocystidia absent. Pileipellis a cutis composed of cylindrical, hyaline, frequently branched hyphae 4–15 μm wide; terminal elements obtuse, 22–89 × 4–15 μm. Annular hyphae cylindrical, hyaline, 3–8 μm wide, frequently branched, terminal elements obtuse, 25–66 × 5–8 μm. Clamp connections absent.

Habitat: Solitary, scattered, or gregarious on humus-rich soil in grasslands, woodland margins, plantations, and disturbed habitats, fruiting from early June to early September.

Distribution in China: Beijing, Inner Mongolia, Jilin, Heilongjiang, Jiangsu, Yunnan, Tibet, Gansu, and Xinjiang [14].

Global distribution: China [14], Denmark, Sweden [45], Netherlands, UK, Spain [16], Australia, France, Greece, Canada, and USA [53].

Specimens examined. CHINA. Xinjiang Uygur Autonomous Region: Ili Kazakh Autonomous Prefecture, Tekes County, Qiongkushitai, 2285 m elev., 10 July 2023, Qi ZX FJAU66955; Ili Kazakh Autonomous Prefecture, Tekes County, Qiongkushitai, 2285 m elev., 20 August 2023, Qi ZX FJAU66975; Ili Kazakh Autonomous Prefecture, Tekes County, Aketamu Wetland, 1285 m elev., 8 August 2024, Qi ZX FJAU66866; Ili Kazakh Autonomous Prefecture, Tekes County, Aketamu Wetland, 1285 m elev., 18 August 2024, Qi ZX FJAU66868, FJAU66870; Ili Kazakh Autonomous Prefecture, Tekes County, Aketamu Wetland, 1285 m elev., 26 August 2024, Qi ZX FJAU66872, FJAU66873; Hotan Prefecture, Pishan County, jujube orchard, 3 June 2025, Zhang B FJAU66887; and Hotan Prefecture, Pishan County, jujube orchard, 4 September 2025, Zhang B FJAU66929.

Notes. The examined material fits the current concept of *A. bisporus*, especially in its predominantly two-spored basidia and relatively broad basidiospores [16,28,54,55]. Intraspecific mean p-distances were 0.0019 for ITS, 0.0022 for LSU, and 0.0128 for *tef1*.

Macromorphological variation in pileus coloration and squamulation is observed among the Xinjiang collections, but all specimens fall within the accepted morphological range of the species [56,57]. *A. bisporus* may be confused with *A. bitorquis* and *A. subfloccosus*. It differs from *A. bitorquis* in having a median, single to pseudo-double annulus rather than an inferior, distinctly multilayered annulus [14]. It differs from *A. subfloccosus* by its slightly larger basidiospores [28]. These distinctions are supported by ITS p-distances of 0.040669 from *A. bitorquis* and 0.018351 from *A. subfloccosus*.

***Agaricus sinodeliciosus*** **Z.R. Wang & R.L. Zhao, Phytotaxa 202(3): 192 (2015).**

**Figure 9:**
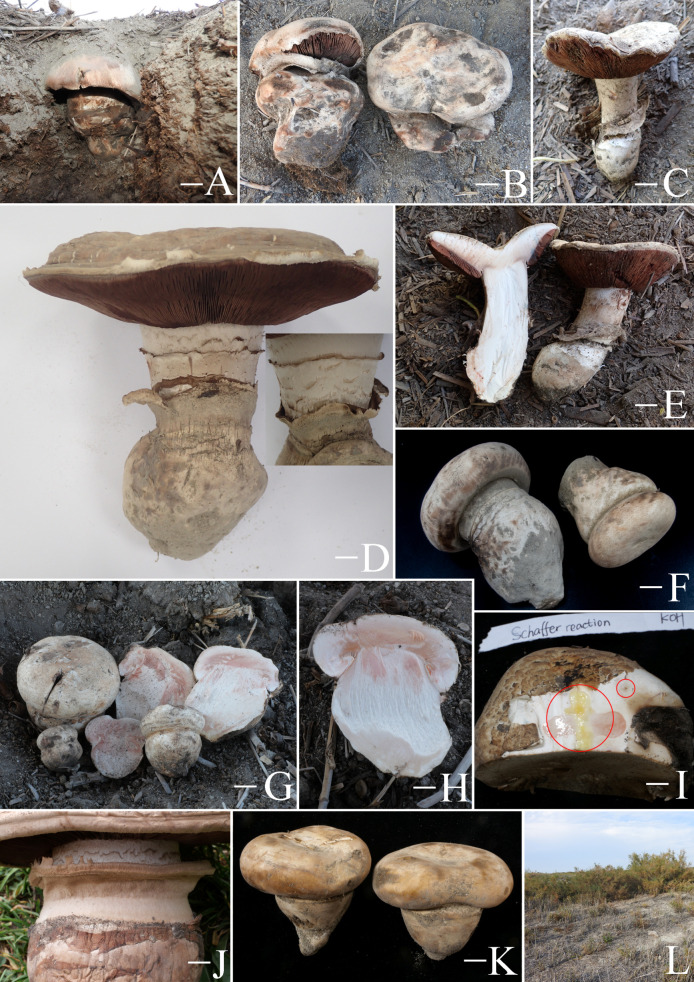
*Agaricus sinodeliciosus* in situ. (**A**–**H**,**J**–**K**): Morphological features under normal environmental conditions. (**I**): Chemical reactions (left: Schäffer’s reaction; right: KOH reaction). (**L**): Habitat. (**A**,**B**): Photographs by Jia PS. (**C**–**L**): Photographs by Qi ZX. Scale bars: (**A**–**K**) = 10 mm.

**Figure 10:**
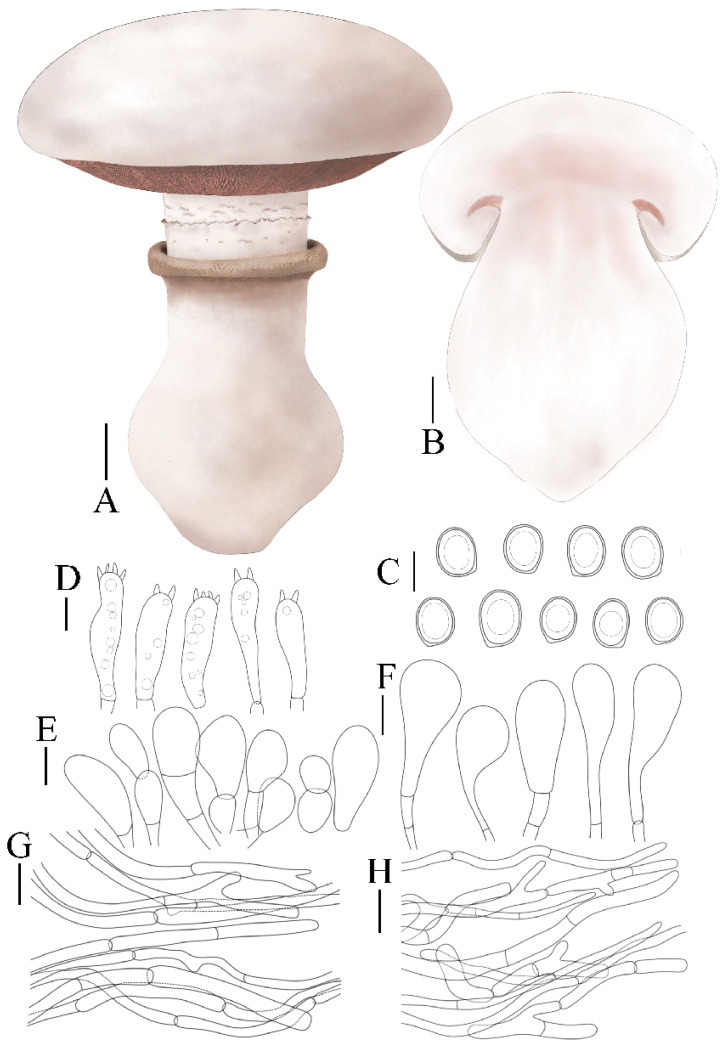
Macro- and micromorphology of *Agaricus sinodeliciosus*. (**A**): Basidiomata. (**B**): Longitudinal section. (**C**): Basidiospores. (**D**): Basidia. (**E**): Cheilocystidia. (**F**): Pleurocystidia. (**G**): Pileipellis hyphae. (**H**): Annulus hyphae. Scale bars: (**A**,**B**) = 10 mm, (**C**) = 5 μm, (**D**–**H**) = 10 μm.

Figure 9 and Figure 10, Figure 23G,H.

Description. Basidiomata medium to large, hypogeous to semi-hypogeous when young, initially buried in desert sandy soil or reed marsh peat, sometimes occurring 200–500 mm below the surface, mature basidiomata usually developing closer to the soil surface or partially emerging. Pileus 70–280 mm diam., initially spherical to hemispherical, smooth, white to pale yellow (1A1–3B3); margin incurved; at maturity, convex to plano-convex or slightly depressed at center, clay-yellow to ochre-yellow (1B2–4C4), covered with appressed, dark brown (6F6), block-like to angular scales; margin sometimes with veil remnants; surface bruising pale reddish brown (8E7). Lamellae free, crowded, unequal, with 2–5 series of lamellulae, 4–8 mm wide, edge smooth, initially white to pale pink, becoming reddish brown to dark brown at maturity. Stipe 67–192 × 54–90 mm, cylindrical, solid; base swollen and bulbous to 120 mm wide, surface smooth when young, white to pale yellow; at maturity, smooth or with pale brown annular scales above annulus and smooth to pale brown scaly below annulus, bruising pale reddish brown. Annulus inferior, boot-shaped, membranous, double-layered, ascending and opening upward, upper annulus 3–7 mm wide; upper surface white and striate, lower surface pale brown with fibrillose scales; lower annulus similar, formed during stipe elongation. Context white, 7–20 mm thick, firm, turning reddish brown when cut, less distinctly so at stipe base. Odor strongly fragrant, mushroom-like. Spore print dark brown. KOH reaction negative, Schäffer’s reaction negative.

Basidiospores [500/60/33] (5.5–)6–7.5(–8) × 4.5–6(–6.5) μm, avL × avW = 6.61 × 5.40 μm, Q = (1.08–)1.09–1.35(–1.40), avQ = 1.23, mostly subglobose to broadly ellipsoid or ellipsoid, brown to dark brown, wall 0.5–1 μm thick, occasionally with oil drops, apiculus distinct. SEM: basidiospores smooth, unornamented, subglobose to broadly ellipsoid in outline, with a distinct apiculus. Basidia (27–)29–35(–39) × (7–)8–11 μm, clavate, hyaline or brown pigment; 4(2)-sterigmata, 2–5 μm long. Cheilocystidia abundant, (9–)12–26(–27) × (7–)9–14(–15) μm, clavate to irregularly clavate, often forked at apex or middle part, usually with an elongated stalk, thin-walled, hyaline or with pale brown pigment. Pleurocystidia (33–)35–55(–57.5) × (11–)12–20(–22) μm, clavate, thin-walled, hyaline when young, with brown pigment at maturity. Pileipellis a cutis composed of cylindrical, frequently branched hyphae 4–10 μm wide, slightly constricted at septa, hyaline to pale brownish, terminal elements slightly acute to obtuse, 38–63 × 5–8 μm. Hyphae of upper and lower annuli similar, cylindrical, hyaline to brownish pigmented, 6–8 μm wide, frequently branched and interwoven, slightly constricted at septa, terminal elements obtuse, 33–105 × 6–9 μm. Clamp connections absent.

Habitat: Gregarious, hypogeous to semi-hypogeous in sandy soils at margins of Gobi Desert or in peat soils of dried-up reed beds dominated by *Phragmites australis* var. *isiaca*, fruiting mainly in spring and autumn.

Distribution in China: Xinjiang, Inner Mongolia, and Qinghai [12].

Global distribution: China [12].

Specimens examined. CHINA. Xinjiang Uygur Autonomous Region: Bortala Mongol Autonomous Prefecture, Jinghe County, Ebinur Lake, 11 May 2023, Jia PS FJAU66856, FJAU66857; Bortala Mongol Autonomous Prefecture, Jinghe County, Ebinur Lake, 23 September 2023, Jia PS FJAU66772, FJAU66773; Bortala Mongol Autonomous Prefecture, Jinghe County, Ebinur Lake, 27 September 2023, Jia PS FJAU66739, FJAU66744, FJAU66747, FJAU66748, FJAU66750, FJAU66762, FJAU66860, FJAU66862; Bortala Mongol Autonomous Prefecture, Jinghe County, Ebinur Lake, 11 May 2024, Jia PS FJAU66840; Bortala Mongol Autonomous Prefecture, Jinghe County, Ebinur Lake, 13 May 2024, Jia PS FJAU66806, FJAU66807, FJAU66809, FJAU66810, FJAU66811, FJAU66833; Ili Kazakh Autonomous Prefecture, Nilka County, 14 May 2024, Jia PS FJAU66817; Bortala Mongol Autonomous Prefecture, Jinghe County, Ebinur Lake, 23 September 2024, Jia PS FJAU66774, FJAU66782, FJAU66805; Bortala Mongol Autonomous Prefecture, Jinghe County, Ebinur Lake, 3 September 2025, Qi ZX & Zhang B FJAU66920, FJAU66923, FJAU66924, FJAU66926; and Bortala Mongol Autonomous Prefecture, Jinghe County, Ebinur Lake, 14 September 2025, Qi ZX & Zhang B FJAU66942, FJAU66943, FJAU66944, FJAU66945.

Notes. The Xinjiang material is consistent with previous descriptions of *A. sinodeliciosus* in its large hypogeous to semi-hypogeous basidiomata, thick context, relatively large basidiospores, and presence of pleurocystidia [12]. Intraspecific mean p-distances were 0.0003 for ITS, 0.0067 for LSU, and 0.0023 for *tef1*.

The pleurocystidia in the present collections are generally larger than the cheilocystidia and lack apical projections, whereas the protologue reported some pleurocystidia with apical outgrowths. This difference is treated here as intraspecific variation. In the multilocus phylogeny, the Xinjiang collections clustered with the holotype and reference specimens of *A. sinodeliciosus* (Figure 1A). This species differs from *A. subsubensis* by its hypogeous to semi-hypogeous habit, larger basidiomata, thicker context, and clavate pleurocystidia [58]. The ITS p-distance between the two species was 0.0180.

***Agaricus subperonatus*** **(J.E. Lange) Singer, Lilloa 22: 432 (1951)**.

**Figure 11:**
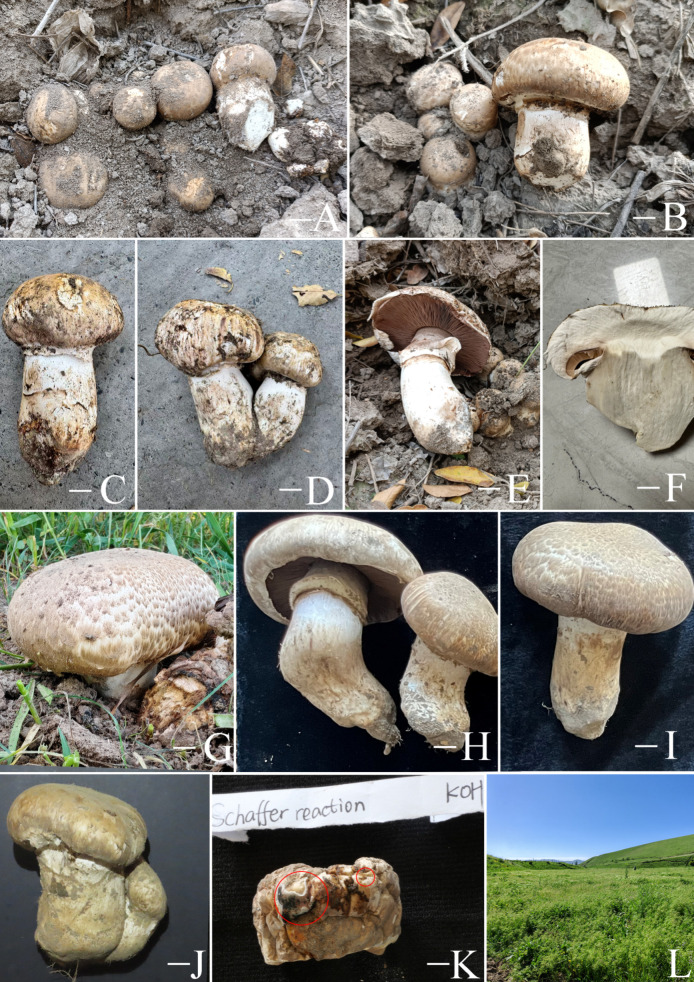
*Agaricus subperonatus* in situ. (**A**–**F**): Specimens collected from Gleditsia forest. (**G**–**J**): Specimens collected from grassland. (**K**): Chemical reactions (left: Schäffer’s reaction; right: KOH reaction). (**L**): Habitat. (**A**,**B**): Photographs by Wu DM. (**C**–**L**): Photographs by Qi ZX. Scale bars: (**A**–**K**) = 10 mm.

**Figure 12:**
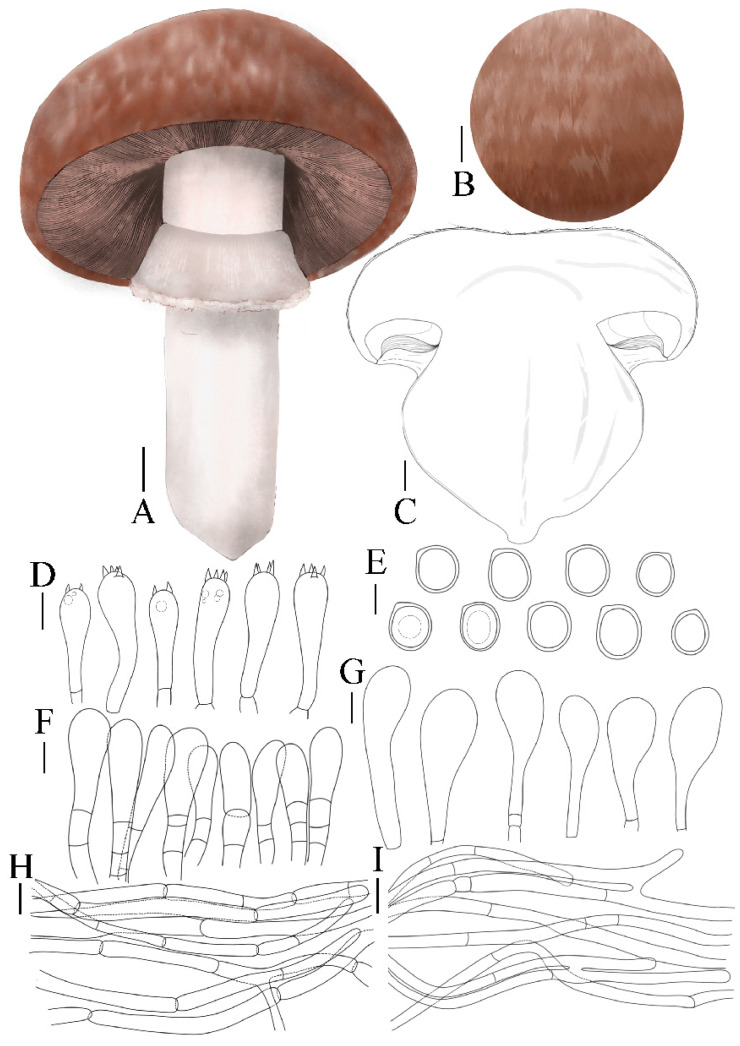
Macro- and micromorphology of *Agaricus subperonatus*. (**A**): Basidiomata. (**B**): Close-up of pileus. (**C**): Longitudinal section. (**D**): Basidia. (**E**): Basidiospores. (**F**): Cheilocystidia. (**G**): Pleurocystidia. (**H**): Pileipellis hyphae. (**I**): Annulus hyphae. Scale bars: (**A**–**C**) = 10 mm, (**E**) = 5 μm, (**D**,**F**–**I**) = 10 μm.

Figure 11 and Figure 12, Figure 23I,J.

Description. Basidiomata medium to large, buried to semi-buried when young, with pileus emerging above ground at maturity. Pileus 50–150 mm diam., initially spherical to hemispherical, dark brown to reddish brown (7F7–9E7), smooth, margin with white veil remnants; at maturity, plano-convex to discoid, white to dirty white, covered with concentrically arranged, imbricate, angular, fibrillose scales, becoming more densely arranged toward the pileus center, dark brown to reddish brown, margin usually exceeding lamellae by 3–4 mm, without veil remnants. Pileus surface unchanged when bruised. Lamellae free, crowded, with 2–3 series of lamellulae, 4–7 mm wide; edge slightly serrate; initially grayish white to pink, becoming brown to dark brown. Stipe 35–150 × 15–30 mm, clavate to cylindrical, solid, base attenuate, often with universal veil remnants; above annulus smooth, white; below annulus with pale brown fibrillose covering. Rhizomorphs absent. Annulus superior, single- to pseudo-double-layered, fugacious, skirt-like, 3–6 mm wide, 2–4 mm thick, descending; upper surface white and striate, lower surface fibrillose; in dried specimens, appressed to stipe. Context white, 5–9 mm thick, firm, fleshy, becoming grayish white immediately after cutting. Odor fragrant, mushroom-like. KOH reaction negative, Schäffer’s reaction negative. Spore print brown.

Basidiospores [300/46/30] (5.2–)5.5–7.5(–8.2) × (4.5–)4.8–6.0(–7.0) μm, avL × avW = 6.30 × 5.20 μm, Q = 1.10–1.35, avQ = 1.20, mostly subglobose to broadly ellipsoid, rarely ellipsoid, dark brown, with apiculus. SEM: basidiospores smooth, unornamented, subglobose to ellipsoid in outline, with a distinct apiculus. Basidia 25–36 × 8–14 μm, clavate to broadly clavate, with 4(2)-sterigmata, 4 μm long, hyaline. Cheilocystidia abundant, gregarious, 21–53 × 7–13 μm, mostly clavate to subclavate, rarely fusiform, thin-walled, smooth, hyaline to pale brown; base with 1–2 septa. Pleurocystidia few, scattered, 31–49 × (10–)12–18(–23) μm, clavate, thin-walled, hyaline when young, with brown pigment at maturity. Pileipellis a cutis composed of cylindrical, hyaline to pale brown hyphae 4–10 μm wide, frequently branched, constricted at septa, terminal elements obtuse to slightly swollen, 41–79 × 5–12 μm. Annular hyphae cylindrical, hyaline, 3–7 μm wide, frequently branched, constricted at septa, terminal elements obtuse, 57–88 × 5–9 μm. Clamp connections absent.

Habitat: Scattered or clustered on open ground in grassland and *Gleditsia* woodland with thick humus layers, basidiomata initially buried and emerging at maturity, fruiting in spring and autumn.

Distribution in China: Xinjiang [14].

Global distribution: China [14], Denmark [54], Italy [16], USA and Canada [8].

Specimens examined. CHINA. Xinjiang Uygur Autonomous Region: Ili Kazakh Autonomous Prefecture, Nilka County, 14 May 2024, Jia PS FJAU66812, FJAU66813, FJAU66815, FJAU66816, FJAU66818, FJAU66819, FJAU66820, FJAU66821, FJAU66823, FJAU66824, FJAU66826, FJAU66831, FJAU66834, FJAU66835, FJAU66836, FJAU66837, FJAU66838, FJAU66839, FJAU66849, FJAU66853, FJAU66854, FJAU66855; Kuitun City, 716 m elev., 30 September 2025, Qi ZX FJAU66946, FJAU66947, FJAU66948, FJAU66949, FJAU66950; and Changji Hui Autonomous Prefecture, Manas County, 678 m elev., 30 September 2025, Wu DM FJAU66951, FJAU66952, FJAU66953, FJAU66954.

Notes. *A. subperonatus* is characterized by its concentrically arranged, imbricate, dark brown to reddish-brown fibrillose pileus scales and by a relatively narrow, single to pseudo-double annulus, sometimes appearing horizontally divided by an annular fissure [8,16,59]. Intraspecific mean p-distances were 0.0000 for ITS, 0.0070 for LSU, and 0.0051 for *tef1*.

The present collections are consistent with the protologue and subsequent descriptions, particularly in pileus squamulation and annulus structure. *A. subperonatus* resembles *A. taeniatus*, but the latter has a single annulus, a floccose scaly stipe below the annulus, rusty discoloration on bruising, and wine-brown to pale flesh-colored discoloration in the stipe base context when cut [26]. *A. subperonatus* also differs from its phylogenetic sister species *A. bitorquis* by its scaled pileus and broader basidiospores [14]. ITS p-distances from *A. sinodeliciosus*, *A. taeniatus*, and *A. subsubensis* were 0.0150, 0.0182, and 0.0193, respectively.

***Agaricus*** **subgen.** ***Pseudochitonia*** **sect.** ***Chitonioides.***

***Agaricus gennadii*** **(Chatin & Boud.) P.D. Orton, Trans. Br. mycol. Soc. 43(2): 174, 1960.**

**Figure 13:**
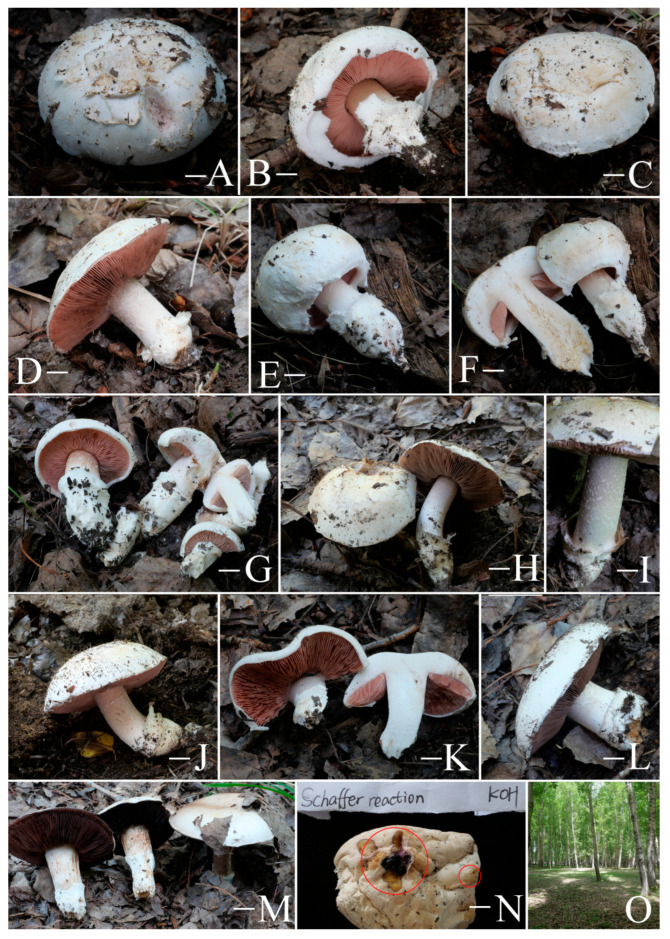
*Agaricus gennadii* in situ. (**A**–**M**): Morphological features under normal environmental conditions. (**N**): Chemical reactions (left: Schäffer’s reaction; right: KOH reaction). (**O**): Growing environment. (**A**–**O**): Photographs by Qi ZX. Scale bars: (**A**–**N**) = 10 mm.

**Figure 14:**
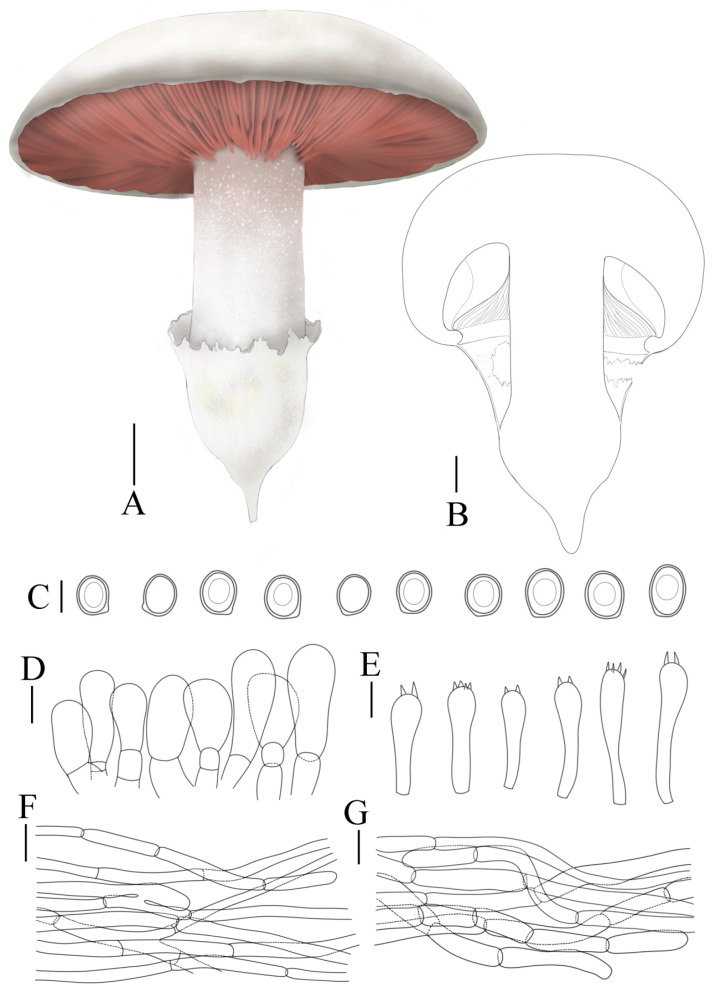
Macro- and micromorphology of *Agaricus gennadii*. (**A**): Basidiomata. (**B**): Longitudinal section. (**C**): Basidiospores. (**D**): Cheilocystidia. (**E**): Basidia. (**F**): Annulus hyphae. (**G**): Pileipellis hyphae. Scale bars: (**A**,**B**) = 10 mm, (**C**) = 5 μm, (**D**–**G**) = 10 μm.

**Figure 24:**
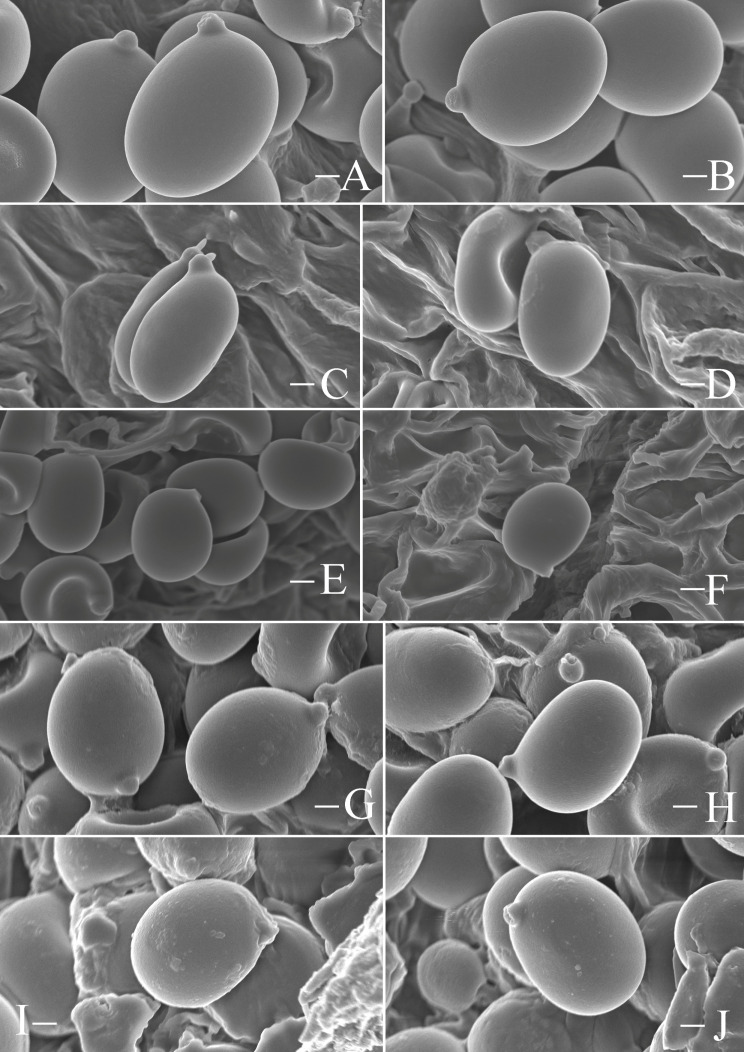
Scanning electron micrographs of basidiospores. (**A**,**B**): *Agaricus gennadii*; (**C**,**D**): *A. cordillerensis*; (**E**,**F**): *A. sylvaticus*; (**G**,**H**): *A. padanus*; (**I**,**J**): *A. desjardinii*. Scale bars = 1 μm.

Figure 13 and Figure 14, Figure 24A,B.

Description. Basidiomata small to large. Pileus 35–147 mm diam., initially subglobose to hemispherical, white to dirty white (1A1); margin incurved, often with white (1A1) remnants of universal veil; at maturity, convex to plano-convex, or discoid, white to pale yellowish brown (1A1–1A3), covered with irregular ochre-yellow (5C5–7) patch-like scales; pileus margin with longitudinal striations, without veil remnants; surface unchanged when cut or bruised. Lamellae free, crowded, 10–15 mm wide, with 2–4 lamellulae, edges slightly serrate, initially white to flesh-pink (1A1–11A2); at maturity, reddish brown to dark brown (8B5–8F8). Stipe 30–79 × 8–21 mm, equal or attenuate toward base becoming fusiform, firm; above annulus covered with longitudinal white (1A1) snakeskin-like striations or scales, sometimes smooth; below annulus with white to pale yellow (1A1–2) floccose scales. Annulus inferior, double- to pseudo-triple-layered, persistent, developing from bottom to top, forming complete sheath-like structure; upper annulus sheath-like with serrate margin, 5–10 mm wide, inner side striate, outer side with white or cream (1A1–2) cottony small scales; lower annulus forming rolled rim around stipe base, fibrillose cottony texture, thick and narrow, 2–4 mm thick, 2–3 mm wide, margin appressed to stipe in undulate manner, split portions resembling double ring, inner surface smooth white (1A1); outer surface fibrillose cottony, grayish white or ochre-yellow (1A1–3A2). Context 7–20 mm thick, firm, white (1A1), turning pale reddish brown (8D8) near lamellae when cut, otherwise unchanging. Chemical reactions: Schäffer’s reaction positive, violaceous (15C6), KOH reaction negative. Odor faint, mushroom-like, unpleasant in old specimens. Spore print brown.

Basidiospores [500/19/12] (6.8–)7.4–8.4(–9.2) × (5.1–)5.5–6.5(–7.1) μm, avL × avW = 7.40 × 6.00 μm, Q = 1.11–1.32(–1.50), avQ = 1.18, mostly subglobose to broadly ellipsoid, dark brown, thick-walled, apiculus prominent. SEM: basidiospores smooth, unornamented, subglobose to broadly ellipsoid in outline, with a conspicuous apiculus. Basidia 25–40 × 6–12(–14) μm, clavate to broadly clavate, with 4(2)-sterigmata, 3–6 μm long; hyaline, containing granular contents. Cheilocystidia abundant, clustered, 17–25(–40) × 9–11(–13) μm, broadly clavate to clavate, thin-walled, hyaline, base with 1–2 septa. Pleurocystidia absent. Pileipellis a cutis, composed of cylindrical hyphae 4–8 μm wide, slightly constricted at septa, hyaline or pale brownish, frequently branched, terminal elements slightly acute at apex, 37–81 × 5–9 μm. Hyphae of upper and lower annuli similar, cylindrical, hyaline, 5–7 μm diam, frequently branched with interwoven branches, slightly constricted at septa; terminal elements obtuse at apex, 33–105 × 6–9 μm. Clamp connections absent.

Habitat: Solitary or scattered on humus-rich soil in *Populus* spp. woodland, fruiting from July to September.

Distribution in China: Qinghai and Xinjiang [14].

Global distribution: Belgium, Cyprus, Spain, France, Netherlands, Hungary, Italy, Portugal, United Kingdom, Czech Republic, Russia, Ukraine [16,56,60], and China [14].

Specimens examined. CHINA. Xinjiang Uygur Autonomous Region: Ili Kazakh Autonomous Prefecture, Tekes County, Aketamu Wetland, 1280 m elev., 9 August 2024, Qi ZX FJAU66867; Ili Kazakh Autonomous Prefecture, Tekes County, Aketamu Wetland, 1280 m elev., 18 August 2024, Qi ZX FJAU66869; Ili Kazakh Autonomous Prefecture, Tekes County, Aketamu Wetland, 1280 m elev., 26 August 2024, Qi ZX FJAU66871; Ili Kazakh Autonomous Prefecture, Tekes County, Aketamu Wetland, 1281 m elev., 20 June 2025, Qi ZX FJAU66906; Ili Kazakh Autonomous Prefecture, Tekes County, Aketamu Wetland, 1280 m elev., 5 September 2025, Qi ZX FJAU66907; and Ili Kazakh Autonomous Prefecture, Tekes County, Aketamu Wetland, 1280 m elev., 12 September 2025, Qi ZX FJAU66910, FJAU66911, FJAU66912, FJAU66913, FJAU66914, FJAU66915, FJAU66916, FJAU66917.

Notes. *A. gennadii* is characterized by firm basidiomata, an inferior sheathing annulus, an attenuate stipe base, relatively large basidiospores, and broadly clavate cheilocystidia [16,61]. Intraspecific mean p-distances were 0.0093 for ITS, 0.0040 for LSU, and 0.0177 for *tef1*.

Among similar species, *A. gennadii* is close to *A. nevoi*, but differs in having broader, more subglobose basidiospores and broadly clavate cheilocystidia. In the present material, basidiospores are subglobose to broadly ellipsoid, avL × avW = 7.4 × 6.0 μm, whereas *A. nevoi* has more ellipsoid and narrower spores, approximately 7.3 × 5.0 μm, and shorter clavate cheilocystidia [61]. The pairwise p-distances between *A. gennadii* and *A. nevoi* were 0.0184 for ITS, 0.0048 for LSU, and 0.0257 for *tef1*. In the phylogenetic analyses, the Xinjiang collections formed a strongly supported clade within sect. *Chitonioides* together with reference material CA339 and HMJAU67811 (Figure 1B).

***Agaricus*** **subgen.** ***Pseudochitonia*** **sect.** ***Sanguinolenti.***

***Agaricus cordillerensis*** **Kerrigan, Mem. N. Y. bot. Gdn 114: 208 (2016).**

**Figure 15:**
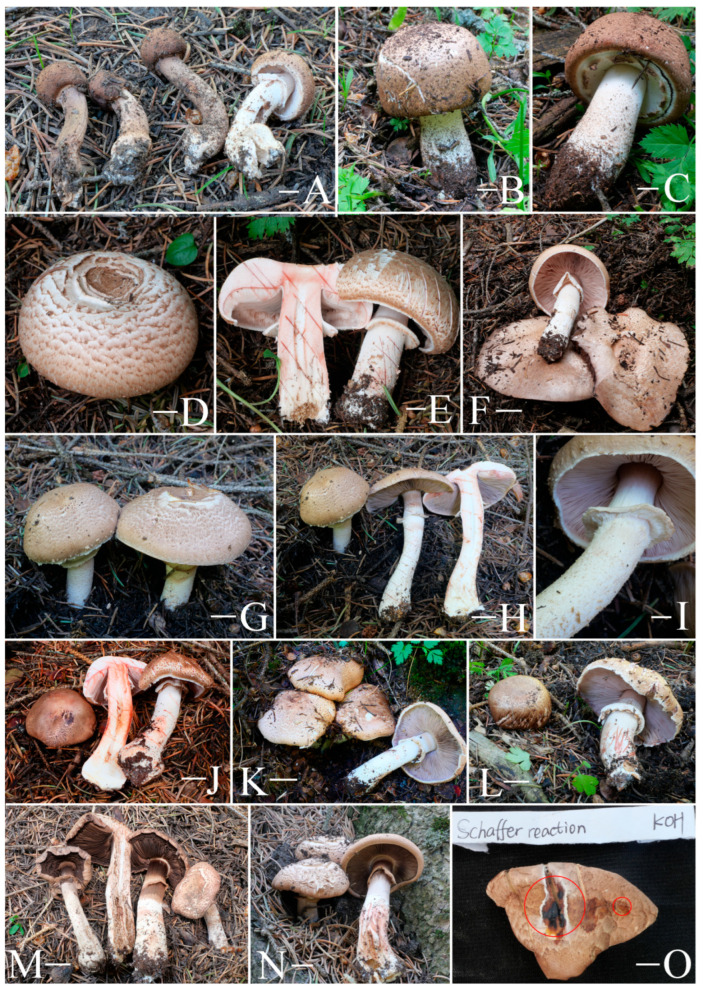
*Agaricus cordillerensis* in situ. (**A**–**E**): Young basidiomata. (**F**–**L**): Mature basidiomata. (**M**,**N**): Old or dehydrated basidiomata. (**O**): Chemical reactions (left: Schäffer’s reaction; right: KOH reaction). (**A**–**O**): Photographs by Qi ZX. Scale bars: (**A**–**O**) = 10 mm.

**Figure 16:**
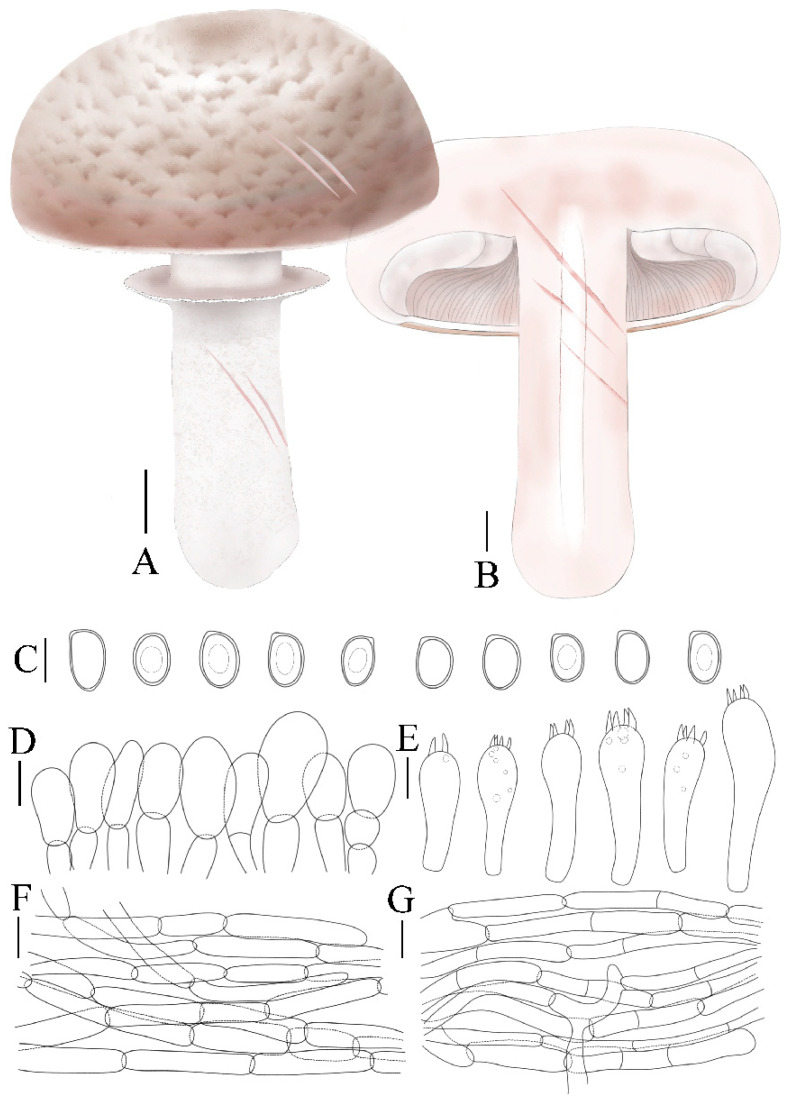
Macro- and micromorphology of *Agaricus cordillerensis*. (**A**): Basidiomata. (**B**): Longitudinal section. (**C**): Basidiospores. (**D**): Cheilocystidia. (**E**): Basidia. (**F**): Pileipellis hyphae. (**G**): Annulus hyphae. Scale bars: (**A**,**B**) = 10 mm, (**C**) = 5 μm, (**D**–**G**) = 10 μm.

Figure 15 and Figure 16, Figure 24C,D.

Description. Basidiomata medium to large. Pileus 37–125 mm diam., initially flattened hemispherical to plano-convex; apex truncate, white (1A1), covered with brown (7E7) flaky scales; margin incurved with veil remnants; at maturity, pileus plano-convex; surface covered with smaller and more abundant scales, distributed concentrically toward margin; center dark brown (7F8); margin paler, ground color white (1A1); pileus surface bruising red (7A8), then fading. Lamellae free, crowded, 4–7 mm wide, with 2–4 lamellulae; edges smooth; initially white to pale pink (1A1–11A2); at maturity, brown to dark brown (8D8–8F8). Stipe clavate, hollow, 70–120 × 12–25 mm, base slightly swollen up to 30 mm wide. Young stipe surface covered with pale brown (6E6) fine scales. At maturity, stipe above annulus smooth, white (1A1). Stipe below annulus covered with white to pale brown (1A1–1B1) fibrillose scales and veil remnants, surface bruising reddish brown (8E7). Old specimens with stipe surface dark brown (8F8). Annulus superior, 4–8 mm wide, 3–5 mm thick, membranous, double-layered, white (1A1), persistent, developing from top to bottom; upper surface smooth; lower surface covered with numerous small white to pale yellowish-brown (1A1–1B1) punctate protrusions, margin irregular and fimbriate; at maturity, annulus pendent, brown (7F8), thin, appressed to stipe surface. Context 6–10 mm thick, firm, white (1A1). Fresh specimens turning bright red (7A8) at junction of stipe and pileus when cut, old or dried specimens turning dark reddish brown (7E8) when cut. Chemical reactions: KOH reaction negative, Schäffer’s reaction negative. Odor mild or slightly pungent. Spore print brown.

Basidiospores [500/45/20] (6–)6.5–7(–8) × (4–)4.5–5 μm, avL × avW = 6.88 × 4.35 μm, Q = 1.33–1.88, avQ = 1.58, mostly ellipsoid to oblong, rarely broadly ellipsoid, brown to dark brown, thick-walled, without germ pore; apiculus minute. SEM: basidiospores smooth, unornamented, ellipsoid to oblong, with a minute but distinct apiculus. Basidia 22–30 × 6–9 μm, clavate to broadly clavate, hyaline; 4(2)-sterigmata, 1–2 μm long. Cheilocystidia abundant, gregarious, 21–40 × 8–17 μm, variable in shape: subcylindrical or clavate, or vesiculose to fusiform, base with 1–3 septa; hyaline to pale brown, thin-walled. Pleurocystidia absent. Pileipellis a cutis, composed of cylindrical hyphae, 5–11 μm wide, frequently branched, slightly constricted at septa; terminal elements obtuse, containing pale brownish intracellular pigment. Annular hyphae cylindrical, 6–9 μm wide, branched, septate, occasionally constricted at septa, containing brown pigment internally; terminal elements 35–79 × 6–8 μm. Clamp connections absent.

Habitat: Solitary or scattered on humus-rich soil in clearings of *P. schrenkiana* forests at 2000–2800 m elevation, fruiting from early July to early September.

Distribution in China: Xinjiang, Inner Mongolia, Sichuan, and Gansu [14].

Global distribution: Canada [8] and China [14].

Specimens examined. CHINA. Xinjiang Uygur Autonomous Region: Ili Kazakh Autonomous Prefecture, Tekes County, Qiongkushitai, 2236 m elev., 11 July 2023, Qi ZX FJAU66960; Ili Kazakh Autonomous Prefecture, Tekes County, Qiongkushitai, 2127 m elev., 14 July 2023, Qi ZX FJAU66963, FJAU66965; Ili Kazakh Autonomous Prefecture, Tekes County, Qiongkushitai, 2587 m elev., 18 August 2023, Qi ZX FJAU66973, FJAU66974; Ili Kazakh Autonomous Prefecture, Tekes County, Qiongkushitai, 2429 m elev., 21 August 2023, Qi ZX FJAU66976; Ili Kazakh Autonomous Prefecture, Tekes County, Qiongkushitai, 2550 m elev., 31 August 2023, Qi ZX FJAU66980; Ili Kazakh Autonomous Prefecture, Tekes County, Qiongkushitai, 2673 m elev., 1 September 2023, Qi ZX FJAU66982; Ili Kazakh Autonomous Prefecture, Tekes County, Qiongkushitai, 2325 m elev., 16 June 2024, Qi ZX FJAU66985; Ili Kazakh Autonomous Prefecture, Tekes County, Qiongkushitai, 2323 m elev., 29 June 2024, Qi ZX FJAU66989; Ili Kazakh Autonomous Prefecture, Tekes County, Qiongkushitai, 2673 m elev., 7 July 2024, Qi ZX FJAU66992; Ili Kazakh Autonomous Prefecture, Tekes County, Qiongkushitai, 2633 m elev., 7 July 2024, Qi ZX FJAU66996, FJAU66998; Ili Kazakh Autonomous Prefecture, Tekes County, Qiongkushitai, 2161 m elev., 14 July 2024, Qi ZX FJAU67001; Ili Kazakh Autonomous Prefecture, Tekes County, Qiongkushitai, 2770 m elev., 3 August 2024, Qi ZX FJAU67010; Ili Kazakh Autonomous Prefecture, Tekes County, Qiongkushitai, 2132 m elev., 4 August 2024, Qi ZX FJAU67013; Ili Kazakh Autonomous Prefecture, Tekes County, Qiongkushitai, 2760 m elev., 28 August 2024, Qi ZX FJAU67021, FJAU67022; Ili Kazakh Autonomous Prefecture, Tekes County, Qiongkushitai, 2171 m elev., 30 August 2024, Qi ZX FJAU67023; and Ili Kazakh Autonomous Prefecture, Tekes County, Qiongkushitai, 2371 m elev., 6 September 2024, Qi ZX FJAU67025.

Notes. The Xinjiang collections fit the current concept of *A. cordillerensis*, particularly in the brown to dark brown fibrillose pileus scales, relatively slender stipe, and context reddening when fresh and becoming reddish brown in older basidiomata [8]. Intraspecific mean p-distances were 0.0002 for ITS, 0.0000 for LSU, and 0.0029 for *tef1*.

The morphology of the Xinjiang specimens is consistent with the type material from Canada, including brown to dark brown fibrillose pileus scales, reddening context, and occurrence in *Picea* forests at high elevations [8]. Some variation in stipe length and pileus squamulation appears to be associated with litter depth and exposure. *A. cordillerensis* differs from *A. sylvaticus* by its mostly ellipsoid to oblong basidiospores and phylogenetic placement [8]. The p-distances between *A. cordillerensis* and *A. sylvaticus* were 0.0144 for ITS, 0.0035 for LSU, and 0.0270 for *tef1*. The Xinjiang collections formed a well-supported clade with the type specimen RWK 1616 (MLBS/BPP = 95/0.97; Figure 1B).

***Agaricus sylvaticus*** **Schaeff., Fung. bavar. palat. nasc. (Ratisbonae) 4: 62 (1774).**

**Figure 17:**
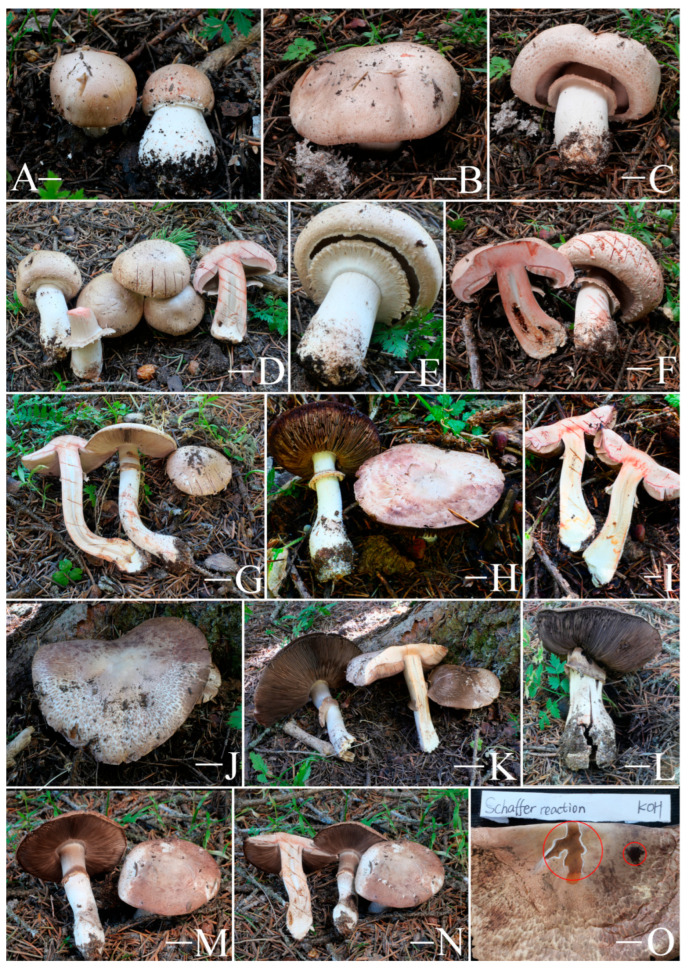
*Agaricus sylvaticus* in situ. (**A**–**F**): Young basidiomata. (**G**–**L**): Mature basidiomata. (**M**,**N**): Old or dehydrated basidiomata. (**O**): Chemical reactions (left: Schäffer’s reaction; right: KOH reaction). (**A**–**O**): Photographs by Qi ZX. Scale bars: (**A**–**O**) = 10 mm.

**Figure 18:**
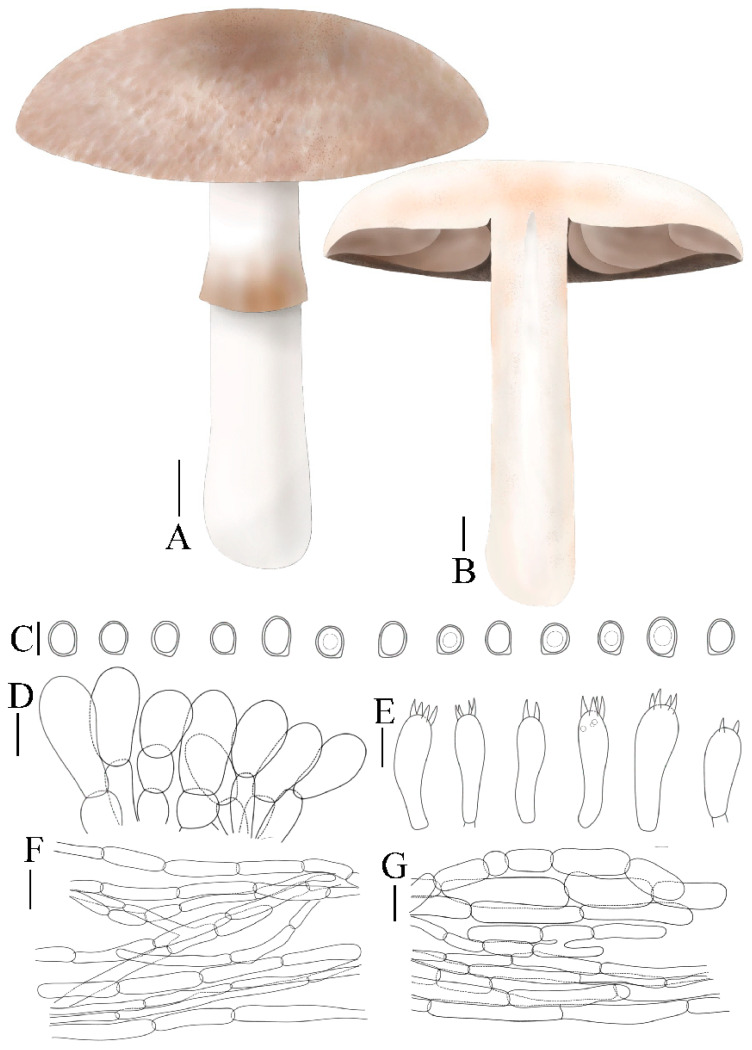
Macro- and micromorphology of *Agaricus sylvaticus*. (**A**): Basidiomata. (**B**): Longitudinal section. (**C**): Basidiospores. (**D**): Cheilocystidia. (**E**): Basidia. (**F**): Pileipellis hyphae. (**G**): Annulus hyphae. Scale bars: (**A**,**B**) = 10 mm, (**C**) = 5 μm, (**D**–**G**) = 10 μm.

Figure 17 and Figure 18, Figure 24E,F.

Description. Basidiomata medium to large. Pileus 25–162 mm diam., initially spherical to hemispherical, flesh-brown (4B3); margin incurved and exceeding lamellae by approximately 2 mm; at maturity, convex or plano-convex, white (1A1); surface covered with ochre-brown or dark brown (8E5–8) fibrillose scales; as pileus expands, scales gradually fragmenting into small triangular fibrillose scales arranged in semi-radiate pattern; pileus surface rapidly discoloring bright red or blood red (9A6–8) when bruised, particularly evident in fresh specimens, then gradually fading. Lamellae free, crowded, approximately 8–10 mm wide, with 2–4 lamellulae; edges smooth, initially white (1A1), then turning gray-pink (1C1); at maturity, brown to dark brown (8F6–8). Stipe 47–122 × 8–18 mm, clavate, hollow; base slightly swollen up to 24 mm; above annulus smooth, white (1A1); below annulus with fine scales; surface bruising bright red to reddish brown (8D6–7) when scraped, then gradually fading. Annulus superior, 12–14 mm wide, 2–4 mm thick, membranous, persistent, simple to pseudo-double-layered, developing from top to bottom; upper surface smooth, brown (7E7); lower surface gray (1B1), covered with fibrils; margin forming regular cogwheel-like pattern; in old specimens, annulus appressed to stipe surface, brown (8E7). Context 3–8 mm thick, white (1A1); fresh specimens immediately turning bright red or blood red (8A7–8) when cut, particularly evident at junction of pileus and stipe context; old specimens turning reddish brown (8D7–8) when cut, then gradually fading. Odor not distinctive. Chemical reactions: KOH reaction negative, Schäffer’s reaction negative. Spore print dark brown.

Basidiospores [500/21/12] (4.5–)5–5.5(–6) × (3.5–)4–4.5 μm, avL × avW = 5.30 × 4.06 μm, Q = 1.11–1.50, avQ = 1.31, mostly subglobose to broadly ellipsoid, rarely ellipsoid, brown, thick-walled, containing 1–2 oil drops; apiculus small. SEM: basidiospores smooth, unornamented, subglobose to broadly ellipsoid in outline, with a small apiculus. Basidia 21–32 × 6.5–9 μm, clavate, with 4(2)-sterigmata, 1–2.5 μm long; hyaline to pale brown. Cheilocystidia abundant, gregarious, 13–20 × 6–12 μm, pyriform to broadly clavate, thin-walled, smooth, with brown intracellular pigment; base with 1–2 septa. Pleurocystidia absent. Pileipellis a cutis, composed of cylindrical hyphae with brown contents, 5–13 μm wide, frequently branched, constricted at septa; terminal elements constricted at apex, 25–98 × 5–14 μm. Lower annular surface hyphae cylindrical, hyaline, 4–9 μm wide, frequently branched, constricted at septa; terminal elements obtuse at apex, 26–53 × 6–7 μm. Clamp connections absent.

Habitat: Solitary or scattered on humus-rich soil in clearings of *P. schrenkiana* forests, fruiting abundantly from July to August.

Distribution in China: Widely distributed in China [14].

Global distribution: China [13,14,62], Denmark, Norway, Sweden, Finland, Netherlands, Germany, Spain [16], USA, Mexico, Brazil, and Australia [8].

Specimens examined. CHINA. Xinjiang Uygur Autonomous Region: Ili Kazakh Autonomous Prefecture, Tekes County, Qiongkushitai, 2643 m elev., 11 July 2023, Qi ZX FJAU66959; Ili Kazakh Autonomous Prefecture, Tekes County, Qiongkushitai, 2098 m elev., 14 July 2023, Qi ZX FJAU66964; Ili Kazakh Autonomous Prefecture, Tekes County, Qiongkushitai, 2476 m elev., 2 August 2023, Qi ZX FJAU66972; Ili Kazakh Autonomous Prefecture, Tekes County, Qiongkushitai, 2473 m elev., 30 August 2023, Qi ZX FJAU66979; Ili Kazakh Autonomous Prefecture, Tekes County, Qiongkushitai, 2679 m elev., 7 July 2024, Qi ZX FJAU66994, FJAU66995, FJAU66997; Ili Kazakh Autonomous Prefecture, Tekes County, Qiongkushitai, 2591 m elev., 13 July 2024, Qi ZX FJAU66999, FJAU67000; Ili Kazakh Autonomous Prefecture, Tekes County, Qiongkushitai, 2166 m elev., 2 August 2024, Qi ZX FJAU67008; Ili Kazakh Autonomous Prefecture, Tekes County, Qiongkushitai, 2525 m elev., 20 August 2024, Qi ZX FJAU67015; and Ili Kazakh Autonomous Prefecture, Tekes County, Qiongkushitai, 2116 m elev., 24 August 2024, Qi ZX FJAU67019.

Notes. *A. sylvaticus* is characterized by brown angular to fibrillose pileus scales, context rapidly reddening when cut or bruised, superior annulus, hollow stipe, subglobose to broadly ellipsoid basidiospores, and pyriform to broadly clavate cheilocystidia [16]. Intraspecific mean p-distances were 0.0038 for ITS, 0.0013 for LSU, and 0.0187 for *tef1*.

The Xinjiang material agrees with the traditional concept of *A. sylvaticus*, although pileus margin veil remnants, reported as common in some European treatments, were rare in the present collections. *A. sylvaticus* is morphologically similar to *A. cordillerensis*, but differs mainly in basidiospore morphology: *A. sylvaticus* has mostly subglobose to broadly ellipsoid basidiospores, whereas *A. cordillerensis* has mostly ellipsoid to oblong basidiospores [14]. The two species also occur in separate phylogenetic clades. The p-distances between them were 0.0144 for ITS, 0.0035 for LSU, and 0.0270 for *tef1*. The Xinjiang specimens formed a well-supported clade with Spanish specimens LAPAG382 and LAPAG341 (MLBS/BPP = 100/1; Figure 1B).

***Agaricus*** **subgen.** ***Pseudochitonia*** **sect.** ***Nigrobrunnescentes.***

***Agaricus padanus*** **Lancon., Riv. Micol. 45(1): 30 (2002).**

**Figure 19:**
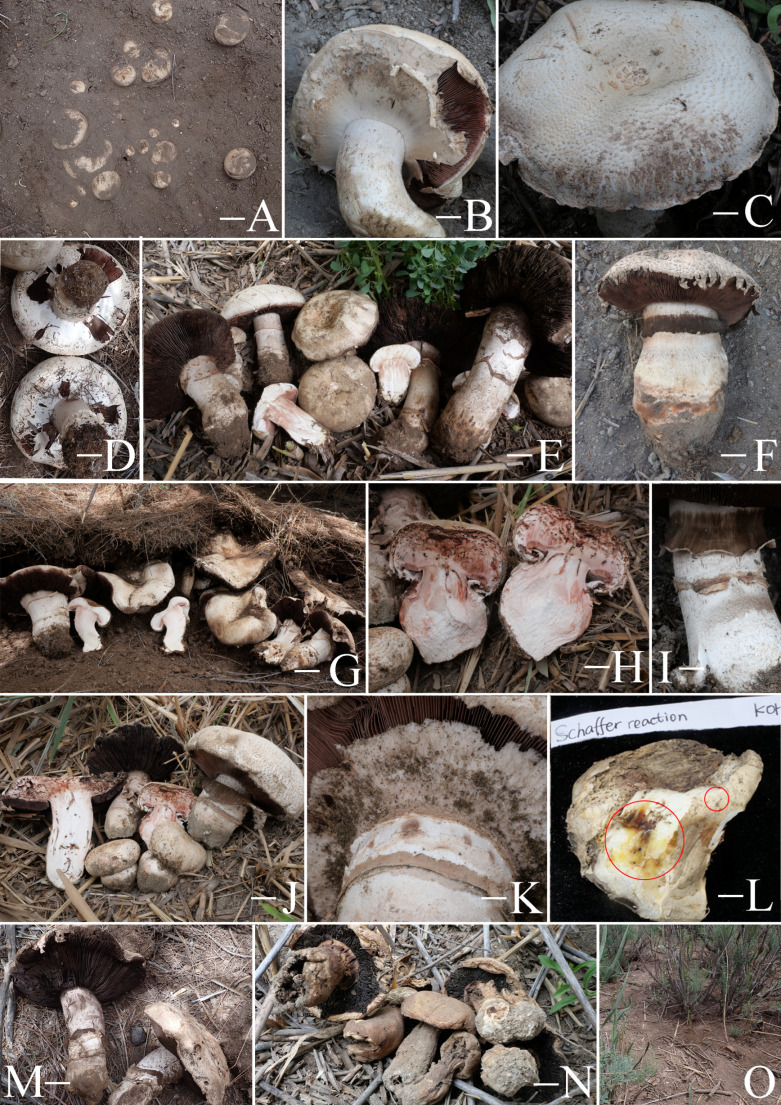
*Agaricus padanus* in situ. (**A**,**D**,**H**): Young basidiomata. (**B**,**C**,**E**–**J**,**M**,**N**): Mature basidiomata. (**K**): Close-up of annulus. (**L**): Chemical reactions (left: Schäffer’s reaction; right: KOH reaction). (**O**): Habitat. (**A**–**O**): Photographs by Qi ZX. Scale bars: (**A**–**N**) = 10 mm.

**Figure 20:**
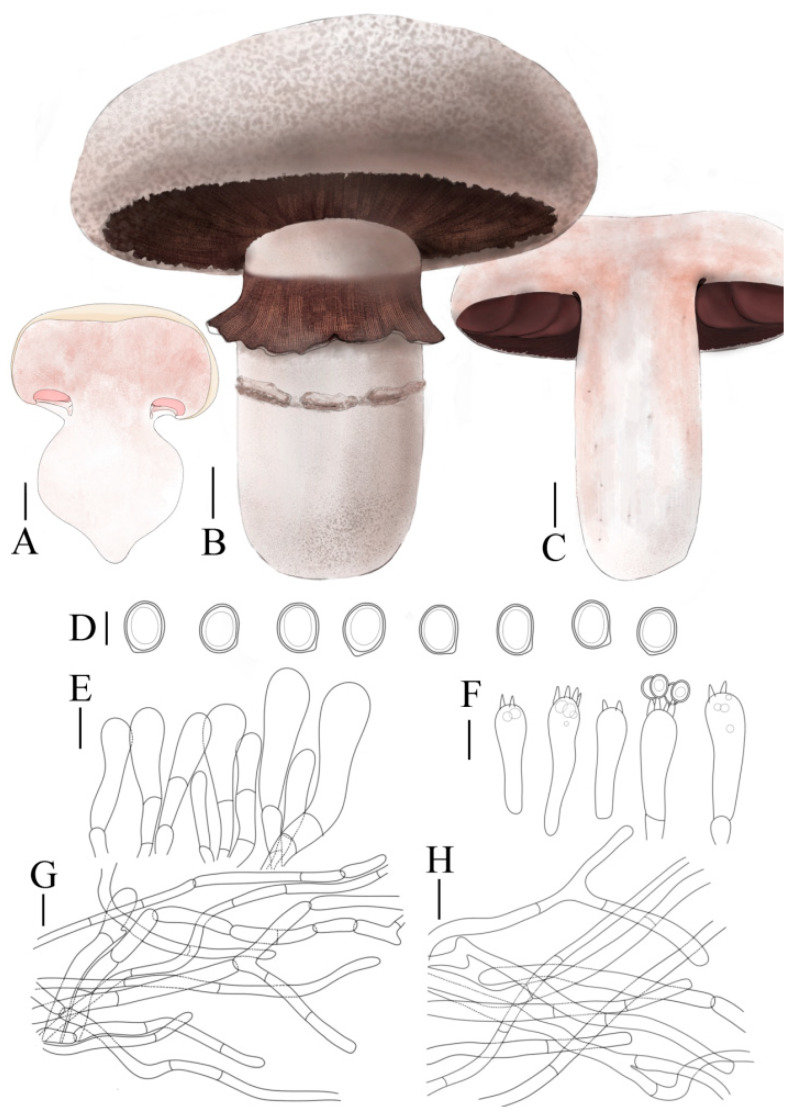
Macro- and micromorphology of *Agaricus padanus*. (**A**): Young basidiomata in longitudinal section. (**B**): Basidiomata. (**C**): Longitudinal section. (**D**): Basidiospores. (**E**): Cheilocystidia. (**F**): Basidia. (**G**): Pileipellis hyphae. (**H**): Annulus hyphae. Scale bars: (**A**–**C**) = 10 mm, (**D**) = 5 μm, (**E**–**H**) = 10 μm.

Figure 19 and Figure 20, Figure 24G,H.

Description. Basidiomata medium to large. Young basidiomata developing 10–30 mm below ground surface, completely covered by universal veil; at maturity, pileus center emerging from soil, or entire basidiomata remaining in soil. Pileus 40–140 mm diam., initially spherical to hemispherical, white to pale yellow (1A1–2), smooth; margin with veil remnants; at maturity, flattened hemispherical to plano-convex; center distinctly depressed; margin undulate, white (1A1); surface covered with appressed pale brown to yellowish-brown (6D5–8) angular scales in concentric arrangement at center; margin with veil remnants; surface bruising reddish brown (9C7). Lamellae free, crowded, unequal with 2–4 lamellulae, narrow, 2–5 mm wide; edges smooth; initially white to pale pink (1A1–11A2); at maturity, brown to dark brown (8D8–8F8). Stipe 40–90 × 30–45 mm, solid, cylindrical and abruptly attenuate; base rounded; above annulus smooth or with fibrillose tomentum, white to dirty white (1A1); below annulus smooth or covered with scaly sheath; at maturity, fragmenting into coarse scales, arranged in rings or sheath-like around stipe surface, pale yellowish brown to dark brown (5E6–5F6). Annulus superior, simple, membranous, 3–6 mm wide, thin, fugacious; margin pendent; upper annular surface dark brown (8F8), striate; lower annular surface with floccose scales, earth gray (1B1). Context 5–12 mm thick, firm, white (1A1), pileus and stipe context margin bruising blood red to red (9A7–8), otherwise unchanged; senescent basidiomata unchanged when bruised. Dried specimens entirely ochre-yellow (3B3). Odor fragrant, mushroom-like. Chemical reactions: KOH reaction negative, Schäffer’s reaction negative. Spore print dark brown.

Basidiospores [500/231/101] (6–)6.5–8 × (5–)5.5–6.5(–7) μm, avL × avW = 7.34 × 5.99 μm, Q = (1.08–)1.18–1.33(–1.38), avQ = 1.23, mostly subglobose to broadly ellipsoid, brown, thick-walled, without germ pore; apiculus indistinct to minute. SEM: basidiospores smooth, unornamented, subglobose to broadly ellipsoid in outline, with an indistinct to minute apiculus. Basidia (28–)35–44(–48) × (8–)8.5–10.5(–12) μm, clavate, hyaline or containing brown pigment; 4-sterigmate, 1–3 μm long. Cheilocystidia abundant, gregarious, (20–)21–32(–39) × (7.5–)8–11(–13) μm, clavate, broadly clavate or irregularly clavate; base often with 1–2 septa; hyaline or with yellowish-brown vacuolar pigment. Pleurocystidia absent. Pileipellis a cutis, made up of 4–8 µm wide hyphae, cylindrical, hyaline or with yellowish-brown wall pigment, occasionally branched, not or slightly constricted at septa. Terminal hyphae obtuse at apex, 17–53 × 3–7 μm, hyaline or with yellowish-brown wall pigment. Annular hyphae cylindrical, hyaline to brownish pigmented, 6–9 μm diam, frequently branched with interwoven branches, slightly constricted at septa; terminal elements obtuse at apex, 6–9 μm diam. Clamp connections absent.

Habitat: Scattered or clustered in peat soils of dried-up *Phragmites australis* var. *isiaca* at Ebinur Lake or in sandy soils of *Haloxylon ammodendron* forests, fruiting in early spring and late autumn.

Distribution in China: Xinjiang and Inner Mongolia [14].

Global distribution: Italy [16] and China [14].

Specimens examined. CHINA. Xinjiang Uygur Autonomous Region: Bortala Mongol Autonomous Prefecture, Jinghe County, Ebinur Lake, 21 September 2023, Jia PS FJAU66760, FJAU66761; Bortala Mongol Autonomous Prefecture, Jinghe County, Ebinur Lake, 23 September 2023, Jia PS FJAU66766, FJAU66768, FJAU66769, FJAU66770; Bortala Mongol Autonomous Prefecture, Jinghe County, Ebinur Lake, 27 September 2023, Jia PS FJAU66738, FJAU66740, FJAU66741, FJAU66742, FJAU66745, FJAU66752, FJAU66754, FJAU66755, FJAU66756, FJAU66858, FJAU66859, FJAU66861; Bortala Mongol Autonomous Prefecture, Jinghe County, Ebinur Lake, 13 May 2024, Jia PS FJAU66808, FJAU66864; Ili Kazakh Autonomous Prefecture, Nilka County, 14 May 2024, Jia PS FJAU66814; Bortala Mongol Autonomous Prefecture, Jinghe County, Ebinur Lake, 23 September 2024, Jia PS FJAU66640, FJAU66641, FJAU66642, FJAU66643, FJAU66644, FJAU66646, FJAU66647, FJAU66648, FJAU66650, FJAU66651, FJAU66652, FJAU66655, FJAU66656, FJAU66657, FJAU66659, FJAU66660, FJAU66661, FJAU66662, FJAU66663, FJAU66664, FJAU66665, FJAU66666, FJAU66668, FJAU66671, FJAU66674, FJAU66675, FJAU66678, FJAU66681, FJAU66682, FJAU66684, FJAU66686, FJAU66687, FJAU66689, FJAU66690, FJAU66691, FJAU66775, FJAU66777, FJAU66778, FJAU66784, FJAU66790; Bortala Mongol Autonomous Prefecture, Jinghe County, Ebinur Lake, 24 September 2024, Jia PS FJAU66694, FJAU66695, FJAU66696, FJAU66697, FJAU66698, FJAU66699, FJAU66700, FJAU66701, FJAU66702, FJAU66703, FJAU66705, FJAU66707, FJAU66708, FJAU66713, FJAU66714, FJAU66715, FJAU66717, FJAU66720, FJAU66721, FJAU66722, FJAU66723, FJAU66726, FJAU66728, FJAU66731, FJAU66734, FJAU66735; Bortala Mongol Autonomous Prefecture, Jinghe County, Ebinur Lake, 31 May 2025, Qi ZX & Zhang B FJAU66875, FJAU66876, FJAU66879, FJAU66881, FJAU66882, FJAU66883, FJAU66886; Bortala Mongol Autonomous Prefecture, Jinghe County, Ebinur Lake, 1 September 2025, Qi ZX & Zhang B FJAU66918, FJAU66919; Bortala Mongol Autonomous Prefecture, Jinghe County, Ebinur Lake, 3 September 2025, Qi ZX FJAU66921, FJAU66922, FJAU66925; and Bortala Mongol Autonomous Prefecture, Jinghe County, Ebinur Lake, 14 September 2025, Qi ZX FJAU66940.

Notes. *A. padanus* is characterized by a pileus with pale yellowish-brown, appressed, angular scales arranged concentrically, a distinctly depressed pileus center, a stipe initially enclosed by a sheath-like universal veil that later breaks into coarse scales, and relatively large basidiospores [16,46]. Intraspecific mean p-distances were 0.0003 for ITS, 0.0033 for LSU, and 0.0038 for *tef1*.

The Xinjiang material corresponds well to the protologue of the species, although several additional features were observed in the present collections. *A. padanus* is morphologically close to *A. sinodeliciosus*, and both occur in arid or semi-arid habitats in Xinjiang. They differ mainly in cystidial morphology and the presence or absence of pleurocystidia: *A. padanus* has clavate, broadly clavate to irregularly clavate cheilocystidia and lacks pleurocystidia, whereas *A. sinodeliciosus* has short clavate to vesiculose or pyriform cheilocystidia and clavate pleurocystidia [12]. In the multilocus phylogeny, the Xinjiang specimens clustered with reference sequences 58-02Lanconelli and WZR2012903 with strong support, confirming the identification.

***Agaricus desjardinii*** Z.R. Wang & R.L. Zhao, Phytotaxa 202(3): 192 (2015).

**Figure 21:**
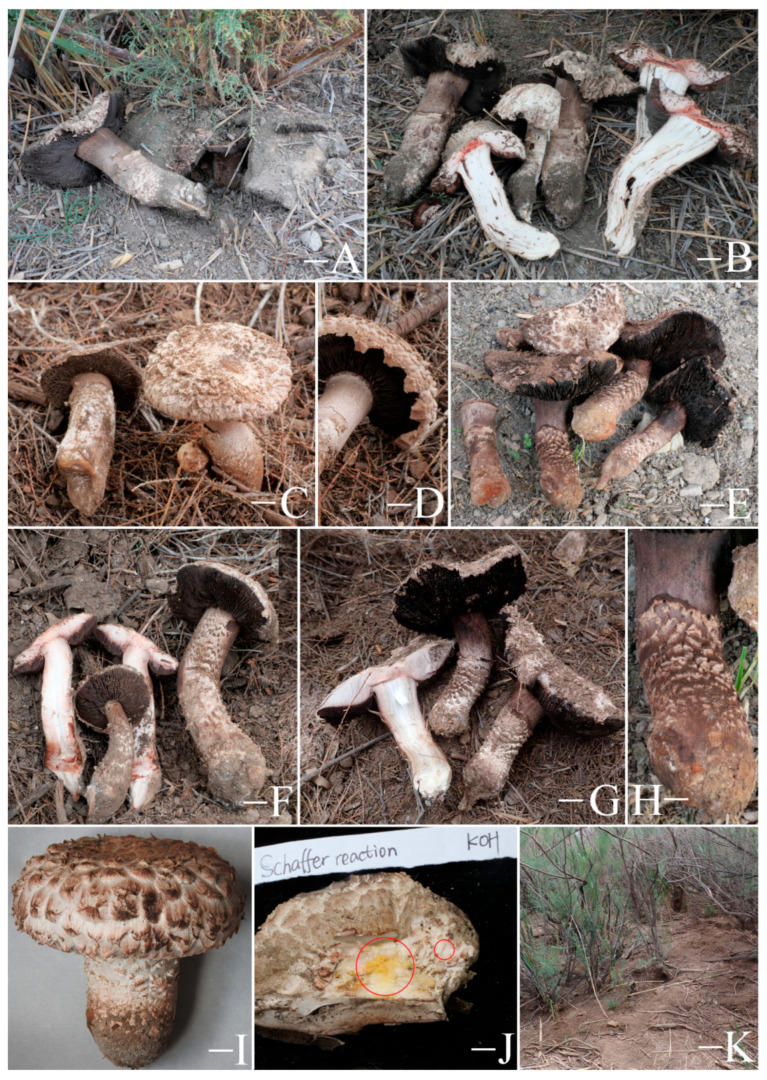
*Agaricus desjardinii* in situ. (**A**–**I**): Basidiomata at different developmental stages and in different field conditions. (**J**): Chemical reactions (left: Schäffer’s reaction; right: KOH reaction). (**K**): Habitat. (**A**–**K**): Photographs by Qi ZX. Scale bars: (**A**–**K**) = 10 mm.

**Figure 22:**
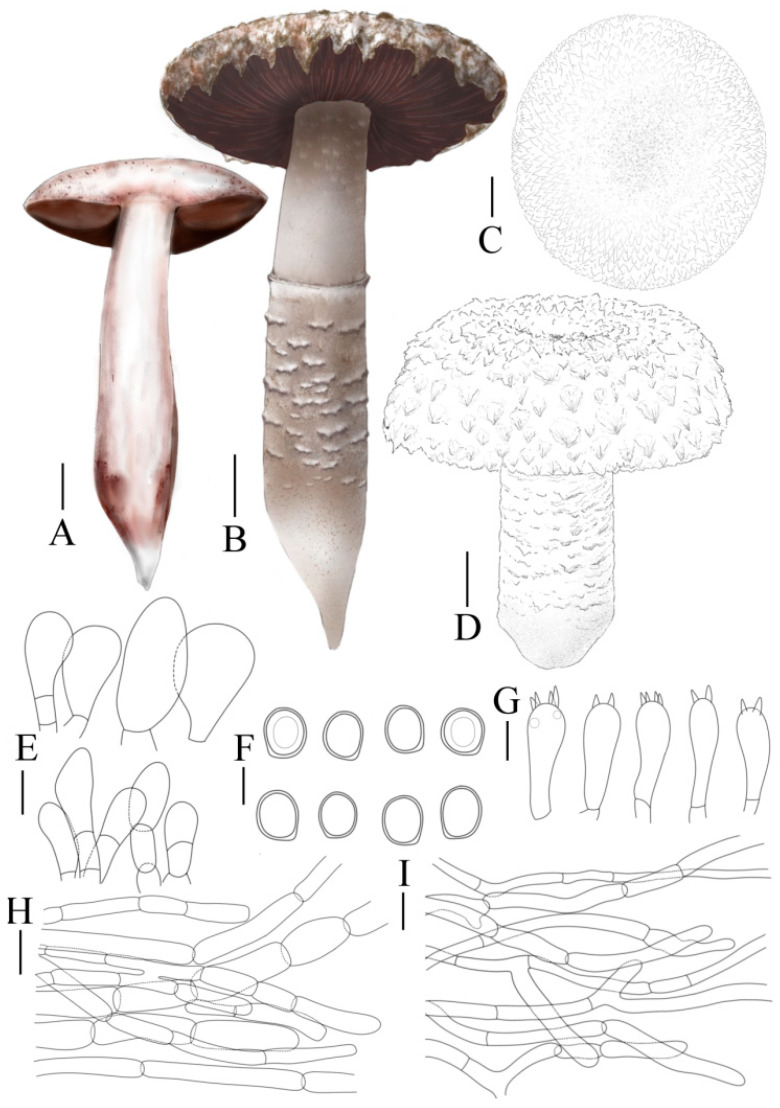
Macro- and micromorphology of *Agaricus desjardinii*. (**A**): Longitudinal section. (**B**): Basidiomata. (**C**): Close-up of pileus. (**D**): Basidiomata. (**E**): Cheilocystidia. (**F**): Basidiospores. (**G**): Basidia. (**H**): Pileipellis hyphae. (**I**): Annulus hyphae. Scale bars: (**A**–**D**) = 10 mm, (**F**) = 5 μm, (**E**,**G**–**I**) = 10 μm.

Figure 21 and Figure 22, Figure 24I,J.

Description. Basidiomata medium to large. Young basidiomata buried in soil; at maturity, pileus emerging from ground. Pileus 43–95 mm diam., initially flattened hemispherical or pulvinate, white (1A1), rough, surface with brown (8F4–6) coarse hairy scales, margin incurved; at maturity, subplano-convex or discoid, white or dirty white (1A1), rough, surface with brown (8F5–8) thick and large fasciculate scales, erect or with recurved apex, margin sometimes with veil remnants; surface bruising rapidly reddish brown to dark brown (8C4–7). Lamellae free, crowded, unequal with 2–3 lamellulae, 2–6 mm wide, edges serrate; at maturity, dark brown (8F8). Stipe 45–90 × 10–28 mm, solid, cylindrical, base attenuate; above annulus white to wine-red (1A1–10E8), smooth, sometimes with brown (7F7) snakeskin-like fine scales; below annulus white to wine-red (1A1–10E8), surface with large brown (7F7) irregular coarse angular squamules; surface bruising or upon desiccation rapidly turning reddish brown (9A6–8), with large rhizomorphs. Annulus median, simple, persistent, thick, membranous, 2–3 mm wide; upper annular surface smooth, lower surface with white floccose scales. Context white (1A1), 2–5 mm thick, firm, pileus context and stipe margin context turning red (9A6–8) when cut. Odor dried specimens with strong mushroom fragrance. Chemical reactions: KOH reaction negative, Schäffer’s reaction negative. Spore print dark brown.

Basidiospores [500/63/45] (5.5–)6–8(–9) × 5–6.5 μm, avL × avW = 6.99 × 5.67 μm, Q = (1.08–)1.09–1.38(–1.42), avQ = 1.23, mostly subglobose to broadly ellipsoid, rarely ellipsoid, brown to dark brown, thick-walled, occasionally with oil drops, apiculus slightly protruding. SEM: basidiospores smooth, unornamented, subglobose to broadly ellipsoid or ellipsoid in outline, with a slightly protruding apiculus. Basidia (25–)26–38.5(–41) × (7–)8–11(–13) μm, clavate, hyaline or containing brown pigment; 4(2)-sterigmata, 2–5 μm long. Cheilocystidia abundant, gregarious, (10–)13.5–32(–34) × 7–19(–20) μm, clavate to broadly clavate, or irregularly clavate, base with 1–2 septa; containing brownish pigment, thin-walled, smooth. Pleurocystidia absent. Pileipellis a cutis, made up of 5–13 µm wide hyphae, cylindrical, branched, septate, occasionally constricted at septa, hyaline, terminal elements 25–60 × 5–13 μm. Annulus composed of hyphae 5–10 μm wide, cylindrical, constricted at septa, hyaline, frequently branched, curved, terminal elements slightly swollen, 5–10 μm wide. Clamp connections absent.

Habitat: Solitary, scattered or gregarious on soil near roots of *Haloxylon ammodendron* at Ebinur Lake, often buried or semi-buried, fruiting in early spring or late autumn.

Distribution in China: Xinjiang [12].

Global distribution: China [12].

Specimens examined. CHINA. Xinjiang Uygur Autonomous Region: Bortala Mongol Autonomous Prefecture, Jinghe County, Ebinur Lake, 11 May 2023, Jia PS FJAU66841, FJAU66845, FJAU66846, FJAU66847; Bortala Mongol Autonomous Prefecture, Jinghe County, Ebinur Lake, 21 September 2023, Jia PS FJAU66759; Bortala Mongol Autonomous Prefecture, Jinghe County, Ebinur Lake, 23 September 2023, Jia PS FJAU66764, FJAU66765; Bortala Mongol Autonomous Prefecture, Jinghe County, Ebinur Lake, 27 September 2023, Jia PS FJAU66743, FJAU66746, FJAU66751, FJAU66753, FJAU66757, FJAU66758, FJAU66863; Bortala Mongol Autonomous Prefecture, Jinghe County, Ebinur Lake, 23 September 2024, Jia PS FJAU66776, FJAU66779, FJAU66780, FJAU66781, FJAU66785, FJAU66786, FJAU66787, FJAU66788, FJAU66789, FJAU66791, FJAU66792, FJAU66793, FJAU66795, FJAU66796, FJAU66797, FJAU66798, FJAU66799, FJAU66800, FJAU66801, FJAU66802, FJAU66804; Bortala Mongol Autonomous Prefecture, Jinghe County, Ebinur Lake, 30 September 2024, Jia PS FJAU66692, FJAU66693; Bortala Mongol Autonomous Prefecture, Jinghe County, Ebinur Lake, 31 May 2025, Qi ZX FJAU66877, FJAU66878, FJAU66880, FJAU66884, FJAU66885; Bortala Mongol Autonomous Prefecture, Jinghe County, Ebinur Lake, 3 September 2025, Qi ZX & Zhang B FJAU66927; and Bortala Mongol Autonomous Prefecture, Jinghe County, Ebinur Lake, 14 September 2025, Qi ZX FJAU66939, FJAU66941.

Notes. *A. desjardinii* is characterized by a pileus with large, recurved squamules, context rapidly bruising reddish brown to brown, a stipe bearing block-like squamules below the annulus, and relatively large basidiospores [12]. Intraspecific mean p-distances were 0.0025 for ITS, 0.0049 for LSU, and 0.0066 for *tef1*.

The species was originally described from Xinjiang, China [12]. The present collections agree with the protologue in most diagnostic characters, although they show several differences: squamules are present on the stipe surface above the annulus, cheilocystidia are abundant, and rhizomorphs occur at the stipe base. In contrast, the protologue described the stipe surface above the annulus as smooth and did not report rhizomorphs or cystidia [12]. These differences are interpreted as intraspecific variation because the Xinjiang collections formed a well-supported clade with the type specimen (MLBS/BPP = 100/1; Figure 1D).

The scanning electron micrographs of basidiospores are shown below (Figure 23 and Figure 24).

## 4. Discussion

The present study reinforces the value of integrative taxonomy for species delimitation in morphologically variable lineages of *Agaricus*. In fungal systematics, ITS remains the primary barcode locus, but its resolving power is uneven among closely related species, and secondary loci are often required for accurate delimitation. Accordingly, our interpretation of species boundaries in Xinjiang *Pseudochitonia* does not rely on sequence divergence alone, but on the congruence among multilocus phylogeny, morphology, chemical reactions, and ecology. This approach is consistent with current recommendations for fungal taxonomy, in which phenotype, genealogy, ecology, and biogeography are treated as complementary rather than competing sources of evidence.

### 4.1. Habitat-Associated Morphological Patterning

PCA, PERMANOVA, and Kruskal–Wallis comparisons (Figure 2; Table 6 and Table 5) revealed significant assemblage-level morphological structuring between the Ebinur Lake desert–wetland assemblage and the Qiongkushitai montane forest assemblage. PERMANOVA showed that assemblage membership explained 34.6% of the total multivariate morphological variation (pseudo-F = 51.83, *p* = 0.0001, *R*^2^ = 0.346), providing multivariate support for the separation observed in PCA. This differentiation was concentrated in traits reflecting basidioma size, robustness, and basidiospore size. Six of the nine measured traits differed significantly between the two assemblages: pileus diameter, pileus context thickness, stipe width, stipe length/width ratio, basidiospore length, and basidiospore width. By contrast, lamella width, stipe length, and annulus width overlapped broadly, indicating that the observed differentiation is not a simple scaling effect across all structures, but is instead concentrated in traits associated with basidioma volume and stipe robustness.

Several ecological and developmental factors may be associated with this pattern, although direct environmental causation cannot be inferred from the present sampling design. Firstly, the semi-hypogeous to hypogeous fruiting habit of Ebinur Lake species (*A. sinodeliciosus*, *A. padanus*, *A. desjardinii*) is likely to impose mechanical demands on basidioma form. Analogous patterns have been discussed for sequestrate and semi-sequestrate fungi more broadly, in which subterranean or partially buried fruiting has evolved repeatedly and is often accompanied by modifications of basidioma form [63]. Agerer [64] noted that subterranean basidioma in multiple Basidiomycota lineages show convergently thickened peridium and context relative to their epigeous relatives. These observations are consistent with the occurrence of thicker pileus context and more robust stipes in the Ebinur Lake assemblage, although whether these traits represent adaptation, phylogenetic legacy, or species-specific morphology remains unresolved. Hypogeous and semi-hypogeous fruiting habits have evolved independently multiple times within sect. *Bivelares* and sect. *Nigrobrunnescentes* [58], and the recurrence of this trait across disparate lineages in the same habitat type is consistent with convergent morphological adaptation rather than shared ancestry.

Secondly, the significantly larger basidiospores in the Ebinur Lake assemblage (mean length 7.07 μm vs. 6.10 μm; mean width 5.86 μm vs. 4.65 μm) may reflect ecological association, developmental constraints, or lineage-specific differences in reproductive morphology. In ephemeral or seasonally extreme environments such as saline wetland margins and desert fringe soils, larger spores with greater lipid reserves may have higher germination success under desiccation stress. Analogous patterns of larger spore size in arid-adapted fungi have been reported in hypogeous genera including *Terfezia* and *Tirmania*, where spore size correlates with substrate aridity and seasonal moisture availability [65]. Whether this difference reflects phenotypic plasticity or fixed lineage-specific traits cannot be resolved without population-level sampling across habitat types.

Thirdly, it should be noted that species identity and habitat category are confounded in the present dataset, because each species was predominantly collected from a single habitat type. The observed morphological differentiation between assemblages therefore cannot be unambiguously attributed to direct habitat effects on individual phenotypes rather than to differences among species with distinct evolutionary histories. Disentangling these contributions will require replicated sampling of the same or congeneric species across contrasting habitats, combined with controlled environmental manipulations or common garden experiments. The present dataset provides a quantitative morphological baseline for such investigations.

### 4.2. Morphological Plasticity and the Interpretation of Diagnostic Characters

Several species in the Xinjiang material exhibited field-level morphological variation that extended beyond previously published descriptions, underscoring the importance of interpreting individual characters within an integrative framework. In *A. xanthodermus*, collections from exposed, dry, or high-altitude sites exhibited a cracked pileus, thickened and appressed annulus, and orange-red lamellae tones. These observations provide additional morphological details that expand the current description of the species [14,16]. That these collections nonetheless retain the diagnostic combination of rapid vivid yellowing, phenolic odor, positive KOH reaction, and broadly ellipsoid basidiospores confirms that the core diagnostic profile of the species is stable, while peripheral macromorphological characters are sensitive to microenvironmental conditions. Similar environmentally driven variation in pileus surface texture and annulus consistency has been documented in other agaricoid species from high-altitude or arid habitats. Under unfavorable environmental conditions, such as low humidity, strong winds, and intense solar radiation, many species may develop a radially cracked pileus. These cracks may later become concentrically arranged and form polygonal hard scales that expose the underlying context, with the extent of exposure depending on pileus flesh thickness. Such features are particularly common in species that fruit in open habitats, including *A. bernardii*, *A. litoralis*, and *A. urinascens* [16,47].

In *A. desjardinii*, cheilocystidia of irregularly clavate form (rather than the simple clavate shape reported in the protologue) and rhizomorphs at the stipe base were observed consistently across multiple collections from Ebinur Lake. Similarly, in *A. sinodeliciosus*, pleurocystidia in the present material consistently lack apical projections documented in original protologue illustrations [12]. In *A. subperonatus*, scattered clavate pleurocystidia were recorded for the first time in Chinese material of this species. These findings extend the known micromorphological range of each taxon. Species boundaries remain defined by the combination of macro- and micromorphological characters and phylogenetic placement. Congruence between molecular and morphological data supports treating these features as intraspecific variation, not taxonomic divergence.

### 4.3. Chemical Reactions as Supporting Evidence in Species Delimitation

Chemical reactions provided useful supporting evidence for sectional placement in the present study. For *A. xanthodermus*, the rapid yellowing of the pileus margin and stipe base, accompanied by a phenolic odor and a positive KOH reaction, is fully consistent with the xanthodermatoid syndrome as defined by Kerrigan [10] for sect. *Xanthodermatei*: yellowing context and pileus surface, phenolic odor at the stipe base, and positive KOH reaction occurring in combination. The Schäffer’s reaction is negative in *A. xanthodermus*, consistent with published descriptions, but the Schäffer’s reaction alone is insufficient for sectional diagnosis and must be evaluated together with the above characters.

For *A. acanthosquamosus* (sect. *Bohusia*), a positive Schäffer’s reaction was consistently observed and is concordant with records from the type species *A. bohusii* [4]. However, a positive Schäffer’s reaction is not uniquely diagnostic for sect. *Bohusia* and has been reported in other sections; sectional placement therefore rests on phylogenetic evidence and the full combination of morphological characters, with the chemical reaction serving as corroborating evidence. For *A. gennadii* (sect. *Chitonioides*), a positive Schäffer’s reaction is consistent with published records [16,61], but the diagnostic profile of this section is defined primarily by the distinctive sheath-like inferior annulus developing from the stipe base upward, subglobose to broadly ellipsoid basidiospores, and broadly clavate cheilocystidia. For the remaining species in this study, negative results for both reactions are consistent with published descriptions and with their respective sectional placements. In all cases, chemical reactions are treated as accessory criteria, subordinate to phylogenetic position and the combination of morphological characters, in accordance with current practice in *Agaricus* taxonomy [5,10,16,66].

### 4.4. Species Delimitation in Agaricus subgen. Pseudochitonia: Integrative Evidence and the Case of A. acanthosquamosus

The recognition of *A. acanthosquamosus* as a new species illustrates the value of combining phylogenetic, morphological, and genetic distance evidence in a lineage where individual characters show overlap among related taxa. Phylogenetically, *A. acanthosquamosus* is recovered as a strongly supported lineage sister to *A. bohusii*, with mean ITS, LSU, and *tef1* interspecific distances of 0.0209, 0.0046, and 0.0282, respectively—values that exceed the intraspecific ranges of both taxa across all three loci. Morphologically, *A. acanthosquamosus* is distinguished from *A. bohusii* by squarrose spinose rather than appressed pileus scales, subglobose to broadly ellipsoid basidiospores (avQ = 1.18) versus ellipsoid basidiospores in *A. bohusii* (Q = 1.32–1.53) [13,16], solitary to scattered rather than fasciculate fruiting habit, and context staining blood red on injury. No single character is diagnostic in isolation, but the character combination is consistently distinct and matches the phylogenetic separation. This illustrates a recurring challenge in *Agaricus* taxonomy: individual characters frequently overlap among related species, requiring diagnosis by character combination [4,5].

The known species treated in this study similarly demonstrate the necessity of an integrative approach. In sect. *Sanguinolenti*, *A. cordillerensis* and *A. sylvaticus* are recovered in separate well-supported clades (MLBS/BPP = 95/0.97 and 100/1, respectively) and are distinguished primarily by basidiospore shape: mostly ellipsoid to oblong in *A. cordillerensis* (avQ = 1.58) versus mostly broadly ellipsoid in *A. sylvaticus* (avQ = 1.31). These quantitative differences in spore shape, while subtle in individual measurements, are consistent across large sample sizes ([500/45/20] and [500/21/12], respectively) and are corroborated by ITS, LSU, and *tef1* divergences of 0.0144, 0.0035, and 0.0270. This case illustrates that morphological characters that appear quantitatively overlapping at the individual level can be reliably diagnostic when evaluated across adequate sample sizes and in conjunction with molecular data.

### 4.5. Biogeographic Context of Xinjiang Pseudochitonia Diversity

The phylogenetic placement of Xinjiang collections within a broader reference framework reveals patterns consistent with the biogeographic position of Xinjiang as an intersection zone between Central Asian, European, and East Asian fungal floras. *A. sylvaticus* collections from Qiongkushitai formed a strongly supported clade with Spanish specimens (LAPAG382, LAPAG341; MLBS/BPP = 100/1) [9], and *A. cordillerensis* clustered with North American type material (RWK 1616; MLBS/BPP = 95/0.97) [8]. The co-occurrence of these taxa in the western Tianshan *P. schrenkiana* forests is consistent with a historical connection between Eurasian temperate conifer forests, and the presence of *A. cordillerensis* —originally described from Canada—in this region suggests either a broader circumboreal distribution than previously recognized or a more complex dispersal history. In contrast, the Ebinur Lake species—*A. sinodeliciosus*, *A. padanus*, and *A. desjardinii*—are currently known from inner Asia only (China, and *A. padanus,* which has also been recorded from Italy), suggesting a more restricted or recently diversified distribution associated with Central Asian arid ecosystems. The co-occurrence of two ecologically distinct assemblages within a single region highlights Xinjiang as a priority area for future surveys of Palaearctic *Agaricus* diversity.

### 4.6. Limitations and Future Directions

Several limitations should be acknowledged. Firstly, the habitat comparison was based on assemblages rather than on replicated populations of the same species across multiple habitat types. Therefore, habitat effects cannot be fully separated from species identity. Secondly, the ecological categories used here are broad. They summarize field conditions but do not replace direct environmental measurements. Thirdly, the genetic distance analyses were used as complementary evidence only. No universal p-distance threshold was applied because divergence varies among loci and among species pairs.

Future studies should include replicated sampling of the same or closely related species across different habitats. They should also incorporate soil properties, salinity, moisture, vegetation cover, and microclimatic data. Population-level sampling and additional loci or genomic data would further help distinguish recent divergence, intraspecific variation, and phenotypic plasticity. Such data will be necessary to test whether the robust, semi-hypogeous basidiomata observed in the Ebinur Lake assemblage represent repeated ecological adaptation or lineage-specific habitat association.

## 5. Conclusions

Ten species of *Agaricus* subgen. *Pseudochitonia* are recognized from Xinjiang, distributed across six sections. Among them, *A. acanthosquamosus* is described as new to science from sect. *Bohusia*, supported by multilocus phylogeny, pairwise genetic distances, and a stable combination of morphological characters distinguishing it from its sister taxon *A. bohusii*. Newly documented morphological variation—including irregularly clavate cheilocystidia in *A. desjardinii*, clavate pleurocystidia in *A. subperonatus*, non-digitate pleurocystidial apices in *A. sinodeliciosus*, and pronounced macromorphological variation in *A. xanthodermus*—extends the known phenotypic range of these taxa. Established species boundaries are unaffected. Quantitative trait comparisons and PERMANOVA revealed significant assemblage-level morphological structuring between the Ebinur Lake desert–wetland assemblage and the Qiongkushitai montane forest assemblage, with assemblage membership explaining 34.6% of the total multivariate morphological variation. These patterns indicate morphological differentiation between the two assemblages. The relative contributions of phenotypic plasticity, local adaptation, and lineage-specific habitat association, however, remain unresolved and require population-level and genomic approaches. This study provides a quantitative morphological baseline and an integrative taxonomic framework for continued investigation of *Pseudochitonia* diversity in the ecologically heterogeneous landscapes of arid northwestern China.

## Figures and Tables

**Table 1:** Primers used for PCR amplification and sequencing in this study.

Locus	Primer	Sequence (5′–3′)	Reference
ITS	ITS1	TCCGTAGGTGAACCTGCGG	[22]
ITS	ITS4	TCCTCCGCTTATTGATATGC	[22]
LSU	LR0R	ACCCGCTGAACTTAAGC	[23]
LSU	LR5	TCCTGAGGGAAACTTCG	[23]
*tef1*	EF1-983F	GCYCCYGGHCAYCGTGAYTTYAT	[9]
*tef1*	EF1-1567R	ACHGTRCCRATACCACCSATCTT	[9]

**Table 2:** Partition information and substitution models used for phylogenetic analyses.

Dataset	Partition	Length (bp)	ML Model
*Agaricus* subgen. *Pseudochitonia*	LSU	909	GTR+I+G
	ITS	728	HKY+I+G
	*tef1*	559	K80+G

**Table 3:** Summary of intraspecific and nearest interspecific p-distances for the Xinjiang taxa of *Agaricus* subgen. *Pseudochitonia*.

Species	Closest Compared Species	ITS Within Mean	ITS Between Mean	LSU Within Mean	LSU Between Mean	*tef1* Within Mean	*tef1* Between Mean
*A. xanthodermus*	*A. moelleri*	0.0001	0.0048	0.0041	–	0.0091	–
*A. bisporus*	*A. qilianensis*	0.0019	0.0077	0.0022	0.0007	0.0128	0.0242
*A. sinodeliciosus*	*A. subsubensis*	0.0003	0.0180	0.0067	–	0.0023	–
*A. subperonatus*	*A. bitorquis*	0.0000	0.0193	0.0070	–	0.0051	–
*A. gennadii*	*A. nevoi*	0.0093	0.0184	0.0040	0.0048	0.0177	0.0257
*A. cordillerensis*	*A. sylvaticus*	0.0002	0.0144	0.0000	0.0035	0.0029	0.0270
*A. sylvaticus*	*A. cordillerensis*	0.0038	0.0144	0.0013	0.0035	0.0187	0.0270
*A. acanthosquamosus*	*A. bohusii*	0.0000	0.0209	0.0013	0.0046	0.0010	0.0282
*A. padanus*	*A. pattersoniae*	0.0003	0.0163	0.0033	–	0.0038	–
*A. desjardinii*	*A. boisseletii*	0.0025	0.0198	0.0049	–	0.0066	–

Notes: “–” indicates that comparable sequence data for the closest compared species were unavailable for that locus.

**Table 4:** Ecological, developmental, and diagnostic matrix of the ten *Agaricus* subgen. *Pseudochitonia* species recorded from Xinjiang.

Species	Section	Main Habitat	Fruiting Habit	Main Vegetation/Substrate	Bruising/Discoloration	KOH	Schäffer’s
*A. acanthosquamosus*	*Bohusia*	Montane forest	Epigeous	Under *P. schrenkiana*, humus-rich soil	Reddening	–	+
*A. xanthodermus*	*Xanthodermatei*	Montane forest/forest edge	Epigeous	*Picea* forest, disturbed soil	Strong yellowing	+	–
*A. cordillerensis*	*Sanguinolenti*	Montane forest	Epigeous	Under conifers, litter-rich soil	Reddening	–	–
*A. sylvaticus*	*Sanguinolenti*	Montane forest	Epigeous	Forest floor, litter-rich soil	Reddening	–	–
*A. bisporus*	*Bivelares*	Riparian/wetland margin/enriched soil	Epigeous to shallowly buried	Organic-rich soil, anthropogenic or ruderal habitats	Weak or absent	–	–
*A. subperonatus*	*Bivelares*	Riparian/wetland margin/disturbed soil	Epigeous to shallowly buried	Rich soil, wetland margin, ruderal ground	Weak yellow-brown or absent	–	–
*A. sinodeliciosus*	*Bivelares*	Desert–wetland/sandy habitat	Semi-hypogeous to hypogeous	Reed wetland margins, sandy–saline soil	Reddish-brown on exposure	–	–
*A. gennadii*	*Chitonioides*	Riparian woodland/open arid transition zone	Epigeous	*Populus* woodland or open soil	Brownish to darkening	–	+
*A. padanus*	*Nigrobrunnescentes*	Desert–wetland/sandy habitat	Semi-hypogeous	Sandy soil, desert fringe	Browning or weak discoloration	–	–
*A. desjardinii*	*Nigrobrunnescentes*	Desert–wetland/sandy habitat	Semi-hypogeous to hypogeous	Sandy or saline substrate	Weak brownish discoloration	–	–

Notes: “+” indicates a positive reaction, and “–” indicates a negative reaction.

**Table 5:** PERMANOVA results for multivariate morphological trait variation between the Ebinur Lake and Qiongkushitai assemblages.

Comparison	Distance Metric	Permutations	Pseudo-F	*R* ^2^	*p* Value
Ebinur vs. Qiongkushitai	Euclidean	9999	51.83	0.346	0.0001

*R*^2^ indicates the proportion of total multivariate morphological variation explained by assemblage membership.

## Data Availability

The newly generated ITS, LSU, and *tef1* sequences have been deposited in GenBank under the accession numbers listed in Supplementary Table S1.

## Supplementary Materials

The following supporting information can be downloaded at: https://www.mdpi.com/article/10.3390/jof12070512/s1, Table S1: Names, collection numbers, reported countries, and corresponding GenBank accession numbers for the taxa used in this study. Sequences generated in this study are shown in bold [1,4,8,9,10,12,27,28,32,58,62,64,67].
