# Supplementary material for: Integrative Taxonomy of Agaricus subgen. Pseudochitonia in Arid Northwestern China: Species Diversity and Habitat-Associated Morphological Differentiation
Source: J Fungi (Basel). 2026 Jul 13;12(7):512. doi: 10.3390/jof12070512 (PMC13412518; doi:10.3390/jof12070512)
Supplement: Supplementary file 1 [file jof-12-00512-s001.zip › jof-4382257-supplementary.pdf]

Supplementary Table S1. Names, collection numbers, reported countries, and corresponding GenBank accession numbers for the taxa used in this study. Sequences generated in this study are shown in bold.

Species	Voucher number	Country	GenBank number			Reference
			ITS	nrLSU	*tef1*	
Agaricus acanthosquamosus	**FJAU66993**	**China**	**PZ101650**	**PZ106824**	**PZ137686**	**This study**
Agaricus acanthosquamosus	**FJAU67024**	**China**	**PZ101656**	**PZ106830**	**PZ137692**	**This study**
Agaricus acanthosquamosus	**FJAU67009**	**China**	**PZ101654**	**PZ106828**	**PZ137690**	**This study**
Agaricus acanthosquamosus	**FJAU67004**	**China**	**PZ101652**	**PZ106826**	**PZ137688**	**This study**
Agaricus acanthosquamosus	**FJAU67028**	**China**	**PZ101657**	**PZ106831**	**PZ137693**	**This study**
Agaricus acanthosquamosus	**FJAU67003**	**China**	**PZ101651**	**PZ106825**	**PZ137687**	**This study**
Agaricus acanthosquamosus	**FJAU67014**	**China**	**PZ101655**	**PZ106829**	**PZ137691**	**This study**
Agaricus acanthosquamosus	**FJAU67005**	**China**	**PZ101653**	**PZ106827**	**PZ137689**	**This study**
Agaricus acanthosquamosus	**FJAU67033**	**China**	**PZ101659**	**PZ106833**	**PZ137695**	**This study**
Agaricus acanthosquamosus	**FJAU67032**	**China**	**PZ101658**	**PZ106832**	**PZ137694**	**This study**
Agaricus agrinferus	RWK 1397 T	USA	EU257801	-	-	[58]
Agaricus amicosus	RWK 2206	USA	KJ577958	-	-	[8]
Agaricus angusticystidiatus	ZRL2043	China	JF691553	-	-	[9]
Agaricus benesii	LAPAG283	France	JF797179	-	-	[9]
Agaricus bernardi	RWK 2086	China	KJ577948	-	-	[8]
Agaricus bernardi	CBS 123 21	Netherlands	MH854692	-	-	[9]
Agaricus bernardiiformis	CA433	China	KT951321	KT951467	KT951577	[4]
Agaricus biberi	LAPAG687	China	KM657919	KR006614	KR006642	[12]

Agaricus bisporus	**FJAU66866**	**China**	**PZ101507**	**PZ106681**	**PZ137543**	**This study**
Agaricus bisporus	**FJAU66872**	**China**	**PZ101510**	**PZ106684**	**PZ137546**	**This study**
Agaricus bisporus	**FJAU66887**	**China**	**PZ101513**	**PZ106687**	**PZ137549**	**This study**
Agaricus bisporus	**FJAU67029**	**China**	**PZ101511**	**PZ106685**	**PZ137547**	**This study**
Agaricus bisporus	**FJAU66868**	**China**	**PZ101508**	**PZ106682**	**PZ137544**	**This study**
Agaricus bisporus	**FJAU66929**	**China**	**PZ101514**	**PZ106688**	**PZ137550**	**This study**
Agaricus bisporus	**FJAU66955**	**China**	**PZ101515**	**PZ106689**	**PZ137551**	**This study**
Agaricus bisporus	**FJAU66975**	**China**	**PZ101516**	**PZ106690**	**PZ137552**	**This study**
Agaricus bisporus	**FJAU66870**	**China**	**PZ101509**	**PZ106683**	**PZ137545**	**This study**
Agaricus bisporus	**FJAU66873**	**China**	**PZ101512**	**PZ106686**	**PZ137548**	**This study**
Agaricus bisporus var. *eurotetrasporus*	Bs423T	France	KF848699	-	-	[9]
Agaricus bitorquis	LAPAG486	China	KM657921	-	-	[12]
Agaricus bitorquis	LAPAG446	China	KM657920	KR006611	KR006640	[12]
Agaricus bitorquis	WZR2012826	China	KM657915	-	-	[12]
Agaricus bitorquis	WZR2012827	China	KM657916	KT951492	KY905100	[12]
Agaricus bohusii	LAPAG562	China	KM657928	KR006613	KR006641	[9]
Agaricus boisseletii	CA123	USA	DQ182531	-	-	[12]
Agaricus brunneofibrillosus	RWK 2037	USA	KJ577963	-	-	[8]
Agaricus caballeroi	AH44503 T	France	KJ575605	-	-	[12]
Agaricus cf. bernardi	CA383	China	KT951319	KT951469	KT951576	[4]
Agaricus collegarum	L0608780 T	USA	NR 198008	-	-	[27]
Agaricus	CA736 T	Iran	KY474564	KY474566	KY474571	[4]

coniferarum						
Agaricus cordillerensis	RWK 2224	USA	KJ577967	-	-	[8]
Agaricus cordillerensis	RWK 2228	USA	KJ577970	-	-	[8]
Agaricus cordillerensis	RWK 2227	USA	KJ577969	-	-	[8]
Agaricus cordillerensis	RWK 2250	USA	KJ577971	-	-	[8]
Agaricus cordillerensis	RWK 2225	USA	KJ577968	-	-	[8]
Agaricus cordillerensis	RWK 2274	USA	KJ577972	-	-	[8]
Agaricus cordillerensis	RWK 2054	USA	KJ577966	-	-	[8]
Agaricus cordillerensis	RWK 1616 T	USA	KJ577965	-	-	[8]
Agaricus cordillerensis	MushroomObserver org 306758	USA	MH091577	-	-	[8]
Agaricus cordillerensis	**FJAU66960**	**China**	**PZ280423**	**PZ273242**	**PZ315553**	**This study**
Agaricus cordillerensis	**FJAU67001**	**China**	**PZ101632**	**PZ106806**	**PZ137668**	**This study**
Agaricus cordillerensis	**FJAU67021**	**China**	**PZ101635**	**PZ106809**	**PZ137671**	**This study**
Agaricus cordillerensis	**FJAU66980**	**China**	**PZ101626**	**PZ106800**	**PZ137662**	**This study**
Agaricus cordillerensis	**FJAU66976**	**China**	**PZ101625**	**PZ106799**	**PZ137661**	**This study**
Agaricus cordillerensis	**FJAU66996**	**China**	**PZ101630**	**PZ106804**	**PZ137666**	**This study**
Agaricus cordillerensis	**FJAU67013**	**China**	**PZ101634**	**PZ106808**	**PZ137670**	**This study**
Agaricus cordillerensis	**FJAU66985**	**China**	**PZ101627**	**PZ106801**	**PZ137663**	**This study**
Agaricus cordillerensis	**FJAU66989**	**China**	**PZ101628**	**PZ106802**	**PZ137664**	**This study**
Agaricus cordillerensis	**FJAU67022**	**China**	**PZ101636**	**PZ106810**	**PZ137672**	**This study**
Agaricus cordillerensis	**FJAU66965**	**China**	**PZ280424**	**PZ273243**	**PZ315554**	**This study**
Agaricus cordillerensis	**FJAU67025**	**China**	**PZ101638**	**PZ106812**	**PZ137674**	**This study**
Agaricus	**FJAU67010**	**China**	**PZ101633**	**PZ106807**	**PZ137669**	**This study**

cordillerensis						
Agaricus cordillerensis	**FJAU66974**	**China**	**PZ280425**	**PZ273244**	**PZ315555**	**This study**
Agaricus cordillerensis	**FJAU67023**	**China**	**PZ101637**	**PZ106811**	**PZ137673**	**This study**
Agaricus cordillerensis	**FJAU66973**	**China**	**PZ101624**	**PZ106798**	**PZ137660**	**This study**
Agaricus cordillerensis	**FJAU66992**	**China**	**PZ101629**	**PZ106803**	**PZ137665**	**This study**
Agaricus cordillerensis	**FJAU66998**	**China**	**PZ101631**	**PZ106805**	**PZ137667**	**This study**
Agaricus cordillerensis	**FJAU66982**	**China**	**PZ280426**	**PZ273245**	**PZ315556**	**This study**
Agaricus cordillerensis	**FJAU66963**	**China**	**PZ280427**	**PZ273246**	**PZ315557**	**This study**
Agaricus cordillerensis	isolate 227	China	ON543710	-	-	[9]
Agaricus crassisquamosus	ZRL2012607 T	China	KT951376	KT951510	KT951645	[9]
Agaricus cupressophilus	MYA-4431 T	USA	NR 111346	-	-	[64]
Agaricus desjardinii	**FJAU66692**	**China**	**PZ101564**	**PZ106738**	**PZ137600**	**This study**
Agaricus desjardinii	**FJAU66776**	**China**	**PZ280519**	**PZ273338**	**PZ315649**	**This study**
Agaricus desjardinii	**FJAU66792**	**China**	**PZ280520**	**PZ273339**	**PZ315650**	**This study**
Agaricus desjardinii	**FJAU66693**	**China**	**PZ101565**	**PZ106739**	**PZ137601**	**This study**
Agaricus desjardinii	**FJAU66743**	**China**	**PZ280521**	**PZ273340**	**PZ315651**	**This study**
Agaricus desjardinii	**FJAU66753**	**China**	**PZ280522**	**PZ273341**	**PZ315652**	**This study**
Agaricus desjardinii	**FJAU66863**	**China**	**PZ101569**	**PZ106743**	**PZ137605**	**This study**
Agaricus desjardinii	**FJAU66751**	**China**	**PZ280523**	**PZ273342**	**PZ315653**	**This study**
Agaricus desjardinii	**FJAU66746**	**China**	**PZ280524**	**PZ273343**	**PZ315654**	**This study**
Agaricus desjardinii	**FJAU66786**	**China**	**PZ280525**	**PZ273344**	**PZ315655**	**This study**
Agaricus desjardinii	**FJAU66764**	**China**	**PZ101567**	**PZ106741**	**PZ137603**	**This study**
Agaricus	**FJAU66759**	**China**	**PZ101566**	**PZ106740**	**PZ137602**	**This study**

desjardinii						
Agaricus desjardinii	**FJAU66791**	**China**	**PZ280526**	**PZ273345**	**PZ315656**	**This study**
Agaricus desjardinii	**FJAU66796**	**China**	**PZ280527**	**PZ273346**	**PZ315657**	**This study**
Agaricus desjardinii	**FJAU66941**	**China**	**PZ101577**	**PZ106751**	**PZ137613**	**This study**
Agaricus desjardinii	**FJAU66788**	**China**	**PZ280528**	**PZ273347**	**PZ315658**	**This study**
Agaricus desjardinii	**FJAU66787**	**China**	**PZ280529**	**PZ273348**	**PZ315659**	**This study**
Agaricus desjardinii	**FJAU66939**	**China**	**PZ101576**	**PZ106750**	**PZ137612**	**This study**
Agaricus desjardinii	**FJAU66802**	**China**	**PZ280530**	**PZ273349**	**PZ315660**	**This study**
Agaricus desjardinii	**FJAU66779**	**China**	**PZ280531**	**PZ273350**	**PZ315661**	**This study**
Agaricus desjardinii	**FJAU66757**	**China**	**PZ280532**	**PZ273351**	**PZ315662**	**This study**
Agaricus desjardinii	**FJAU66847**	**China**	**PZ280533**	**PZ273352**	**PZ315663**	**This study**
Agaricus desjardinii	**FJAU66780**	**China**	**PZ280534**	**PZ273353**	**PZ315664**	**This study**
Agaricus desjardinii	**FJAU66781**	**China**	**PZ280535**	**PZ273354**	**PZ315665**	**This study**
Agaricus desjardinii	**FJAU66878**	**China**	**PZ101571**	**PZ106745**	**PZ137607**	**This study**
Agaricus desjardinii	**FJAU66804**	**China**	**PZ280536**	**PZ273355**	**PZ315666**	**This study**
Agaricus desjardinii	**FJAU66845**	**China**	**PZ280537**	**PZ273356**	**PZ315667**	**This study**
Agaricus desjardinii	**FJAU66793**	**China**	**PZ280538**	**PZ273357**	**PZ315668**	**This study**
Agaricus desjardinii	**FJAU66758**	**China**	**PZ280539**	**PZ273358**	**PZ315669**	**This study**
Agaricus desjardinii	**FJAU66765**	**China**	**PZ280540**	**PZ273359**	**PZ315670**	**This study**
Agaricus desjardinii	**FJAU66846**	**China**	**PZ101568**	**PZ106742**	**PZ137604**	**This study**
Agaricus desjardinii	**FJAU66789**	**China**	**PZ280541**	**PZ273360**	**PZ315671**	**This study**
Agaricus desjardinii	**FJAU66798**	**China**	**PZ280542**	**PZ273361**	**PZ315672**	**This study**
Agaricus	**FJAU66785**	**China**	**PZ280543**	**PZ273362**	**PZ315673**	**This study**

desjardinii						
Agaricus desjardinii	**FJAU66799**	**China**	**PZ280544**	**PZ273363**	**PZ315674**	**This study**
Agaricus desjardinii	**FJAU66800**	**China**	**PZ280545**	**PZ273364**	**PZ315675**	**This study**
Agaricus desjardinii	**FJAU66795**	**China**	**PZ280546**	**PZ273365**	**PZ315676**	**This study**
Agaricus desjardinii	**FJAU66797**	**China**	**PZ280547**	**PZ273366**	**PZ315677**	**This study**
Agaricus desjardinii	**FJAU66801**	**China**	**PZ280548**	**PZ273367**	**PZ315678**	**This study**
Agaricus desjardinii	**FJAU66841**	**China**	**PZ280549**	**PZ273368**	**PZ315679**	**This study**
Agaricus desjardinii	**FJAU66884**	**China**	**PZ101573**	**PZ106747**	**PZ137609**	**This study**
Agaricus desjardinii	**FJAU66885**	**China**	**PZ101574**	**PZ106748**	**PZ137610**	**This study**
Agaricus desjardinii	**FJAU66927**	**China**	**PZ101575**	**PZ106749**	**PZ137611**	**This study**
Agaricus desjardinii	**FJAU66880**	**China**	**PZ101572**	**PZ106746**	**PZ137608**	**This study**
Agaricus desjardinii	WZR2012907	China	KM657901	KT951474	KT951644	[12]
Agaricus devoniensis	CA445	France	EU363036	-	-	[58]
Agaricus dilutibrunneus	ZRL2012010 T	China	KT951358	KT951512	KT951569	[9]
Agaricus erectosquamosus	LD2012165	China	KT951338	KT951509	KT951565	[9]
Agaricus erythrosarx	MURU6080 T	Australia	JF495068	-	-	[32]
Agaricus freirei	CA186 INRA	France	DQ185553	-	-	[9]
Agaricus fuscofibrillosus	WC913	USA	AY484684	-	-	[32]
Agaricus fuscovelatus	RWK 2100	USA	KJ577973	-	-	[12]
Agaricus gennadii	CA339	China	KT951318	-	KT951575	[4]
Agaricus gennadii	**FJAU66867**	**China**	**PZ101592**	**PZ106766**	**PZ137628**	**This study**
Agaricus gennadii	**FJAU66906**	**China**	**PZ280421**	**PZ273240**	**PZ315551**	**This study**
Agaricus gennadii	**FJAU66907**	**China**	**PZ101595**	**PZ106769**	**PZ137631**	**This study**

Agaricus gennadii	**FJAU66914**	**China**	**PZ101600**	**PZ106774**	**PZ137636**	**This study**
Agaricus gennadii	**FJAU66911**	**China**	**PZ101597**	**PZ106771**	**PZ137633**	**This study**
Agaricus gennadii	**FJAU66916**	**China**	**PZ101602**	**PZ106776**	**PZ137638**	**This study**
Agaricus gennadii	**FJAU66871**	**China**	**PZ101594**	**PZ106768**	**PZ137630**	**This study**
Agaricus gennadii	**FJAU66910**	**China**	**PZ101596**	**PZ106770**	**PZ137632**	**This study**
Agaricus gennadii	**FJAU66912**	**China**	**PZ101598**	**PZ106772**	**PZ137634**	**This study**
Agaricus gennadii	**FJAU66917**	**China**	**PZ280422**	**PZ273241**	**PZ315552**	**This study**
Agaricus gennadii	**FJAU66913**	**China**	**PZ101599**	**PZ106773**	**PZ137635**	**This study**
Agaricus gennadii	**FJAU66915**	**China**	**PZ101601**	**PZ106775**	**PZ137637**	**This study**
Agaricus gennadii	HMJAU 67811	China	OR690310	-	-	[62]
Agaricus grandiomyces	ZRL2012611	China	KM657879	-	-	[9]
Agaricus guachari	MA Fungi34253	Netherlands	KR908793	-	-	[9]
Agaricus hondensis	RWK 1938	USA	DQ182513	-	-	[9]
Agaricus hupohanae	SFSU F 020920	USA	NR 144982	-	-	[8]
Agaricus iranicus	VM035 T	Iran	KY474551	KY474557	KY474568	[4]
Agaricus karstomyces	ZRL2011048 T	China	KM657899	KR006632	KR006662	[9]
Agaricus lamellidistans	ZRL3099	China	JF691556	-	-	[9]
Agaricus laparrae	RWK 2039 T	USA	KJ577975	-	-	[8]
Agaricus lusitanicus	LIP 0001283 T	Iran	NR158338	NG058599	-	[4]
Agaricus malangelus	ZRL2012628	China	KM657892	KR006626	KR006655	[9]
Agaricus masoalensis	ZTMyc57137 T	France	KP282686	-	-	[27]
Agaricus menieri	CA162	France	DQ185567	-	-	[10]

Agaricus moelleri	CA209	France	DQ185561	-	-	[10]
Agaricus moellerianus	**FJAU66909**	**China**	**PZ280550**	**PZ273369**	-	**This study**
Agaricus moellerianus	**FJAU66908**	**China**	**PZ280551**	**PZ273370**	-	**This study**
Agaricus moelleroides	LAPAG1167	Spain	MN493149	-	-	[27]
Agaricus nevoi	LAPAG257	China	KM657922	KR006606	KR006635	[4]
Agaricus nigrobrunnescens	DEH632	Netherlands	JX308267	-	-	[12]
Agaricus padanus	**FJAU66640**	**China**	**PZ280431**	**PZ273250**	**PZ315561**	**This study**
Agaricus padanus	**FJAU66650**	**China**	**PZ280432**	**PZ273251**	**PZ315562**	**This study**
Agaricus padanus	**FJAU66651**	**China**	**PZ280433**	**PZ273252**	**PZ315563**	**This study**
Agaricus padanus	**FJAU66665**	**China**	**PZ280434**	**PZ273253**	**PZ315564**	**This study**
Agaricus padanus	**FJAU66775**	**China**	**PZ101582**	**PZ106756**	**PZ137618**	**This study**
Agaricus padanus	**FJAU66752**	**China**	**PZ280435**	**PZ273254**	**PZ315565**	**This study**
Agaricus padanus	**FJAU66664**	**China**	**PZ280436**	**PZ273255**	**PZ315566**	**This study**
Agaricus padanus	**FJAU66642**	**China**	**PZ280437**	**PZ273256**	**PZ315567**	**This study**
Agaricus padanus	**FJAU66832**	**China**	**PZ280438**	**PZ273257**	**PZ315568**	**This study**
Agaricus padanus	**FJAU66784**	**China**	**PZ280439**	**PZ273258**	**PZ315569**	**This study**
Agaricus padanus	**FJAU66726**	**China**	**PZ280440**	**PZ273259**	**PZ315570**	**This study**
Agaricus padanus	**FJAU66760**	**China**	**PZ280441**	**PZ273260**	**PZ315571**	**This study**
Agaricus padanus	**FJAU66708**	**China**	**PZ280442**	**PZ273261**	**PZ315572**	**This study**
Agaricus padanus	**FJAU66754**	**China**	**PZ280443**	**PZ273262**	**PZ315573**	**This study**
Agaricus padanus	**FJAU66699**	**China**	**PZ280444**	**PZ273263**	**PZ315574**	**This study**
Agaricus padanus	**FJAU66808**	**China**	**PZ280445**	**PZ273264**	**PZ315575**	**This study**

Agaricus padanus	**FJAU66684**	**China**	**PZ280446**	**PZ273265**	**PZ315576**	**This study**
Agaricus padanus	**FJAU66681**	**China**	**PZ280447**	**PZ273266**	**PZ315577**	**This study**
Agaricus padanus	**FJAU66701**	**China**	**PZ280448**	**PZ273267**	**PZ315578**	**This study**
Agaricus padanus	**FJAU66738**	**China**	**PZ280449**	**PZ273268**	**PZ315579**	**This study**
Agaricus padanus	**FJAU66740**	**China**	**PZ280450**	**PZ273269**	**PZ315580**	**This study**
Agaricus padanus	**FJAU66702**	**China**	**PZ280451**	**PZ273270**	**PZ315581**	**This study**
Agaricus padanus	**FJAU66641**	**China**	**PZ280452**	**PZ273271**	**PZ315582**	**This study**
Agaricus padanus	**FJAU66697**	**China**	**PZ280453**	**PZ273272**	**PZ315583**	**This study**
Agaricus padanus	**FJAU66671**	**China**	**PZ280454**	**PZ273273**	**PZ315584**	**This study**
Agaricus padanus	**FJAU66643**	**China**	**PZ280455**	**PZ273274**	**PZ315585**	**This study**
Agaricus padanus	**FJAU66721**	**China**	**PZ280456**	**PZ273275**	**PZ315586**	**This study**
Agaricus padanus	**FJAU66722**	**China**	**PZ280457**	**PZ273276**	**PZ315587**	**This study**
Agaricus padanus	**FJAU66647**	**China**	**PZ280458**	**PZ273277**	**PZ315588**	**This study**
Agaricus padanus	**FJAU66814**	**China**	**PZ280459**	**PZ273278**	**PZ315589**	**This study**
Agaricus padanus	**FJAU66663**	**China**	**PZ280460**	**PZ273279**	**PZ315590**	**This study**
Agaricus padanus	**FJAU66756**	**China**	**PZ280461**	**PZ273280**	**PZ315591**	**This study**
Agaricus padanus	**FJAU66682**	**China**	**PZ280462**	**PZ273281**	**PZ315592**	**This study**
Agaricus padanus	**FJAU66940**	**China**	**PZ101580**	**PZ106754**	**PZ137616**	**This study**
Agaricus padanus	**FJAU66881**	**China**	**PZ101589**	**PZ106763**	**PZ137625**	**This study**
Agaricus padanus	**FJAU66886**	**China**	**PZ280463**	**PZ273282**	**PZ315593**	**This study**
Agaricus padanus	**FJAU66728**	**China**	**PZ280464**	**PZ273283**	**PZ315594**	**This study**
Agaricus padanus	**FJAU66734**	**China**	**PZ280465**	**PZ273284**	**PZ315595**	**This study**
Agaricus padanus	**FJAU66731**	**China**	**PZ280466**	**PZ273285**	**PZ315596**	**This study**

Agaricus padanus	FJAU66657	China	PZ280467	PZ273286	PZ315597	This study
Agaricus padanus	FJAU66777	China	PZ280468	PZ273287	PZ315598	This study
Agaricus padanus	FJAU66690	China	PZ280469	PZ273288	PZ315599	This study
Agaricus padanus	FJAU66660	China	PZ280470	PZ273289	PZ315600	This study
Agaricus padanus	FJAU66661	China	PZ280471	PZ273290	PZ315601	This study
Agaricus padanus	FJAU66687	China	PZ280472	PZ273291	PZ315602	This study
Agaricus padanus	FJAU66686	China	PZ280473	PZ273292	PZ315603	This study
Agaricus padanus	FJAU66659	China	PZ280474	PZ273293	PZ315604	This study
Agaricus padanus	FJAU66778	China	PZ101583	PZ106757	PZ137619	This study
Agaricus padanus	FJAU66766	China	PZ101581	PZ106755	PZ137617	This study
Agaricus padanus	FJAU66770	China	PZ280475	PZ273294	PZ315605	This study
Agaricus padanus	FJAU66768	China	PZ280476	PZ273295	PZ315606	This study
Agaricus padanus	FJAU66769	China	PZ280477	PZ273296	PZ315607	This study
Agaricus padanus	FJAU66654	China	PZ280478	PZ273297	PZ315608	This study
Agaricus padanus	FJAU66648	China	PZ280479	PZ273298	PZ315609	This study
Agaricus padanus	FJAU66662	China	PZ280480	PZ273299	PZ315610	This study
Agaricus padanus	FJAU66745	China	PZ280481	PZ273300	PZ315611	This study
Agaricus padanus	FJAU66720	China	PZ280482	PZ273301	PZ315612	This study
Agaricus padanus	FJAU66858	China	PZ101584	PZ106758	PZ137620	This study
Agaricus padanus	FJAU66741	China	PZ280483	PZ273302	PZ315613	This study
Agaricus padanus	FJAU66921	China	PZ280484	PZ273303	PZ315614	This study
Agaricus padanus	FJAU66715	China	PZ280485	PZ273304	PZ315615	This study
Agaricus padanus	FJAU66707	China	PZ280486	PZ273305	PZ315616	This study

Agaricus padanus	**FJAU66714**	**China**	**PZ280487**	**PZ273306**	**PZ315617**	**This study**
Agaricus padanus	**FJAU66876**	**China**	**PZ101587**	**PZ106761**	**PZ137623**	**This study**
Agaricus padanus	**FJAU66646**	**China**	**PZ280488**	**PZ273307**	**PZ315618**	**This study**
Agaricus padanus	**FJAU66652**	**China**	**PZ280489**	**PZ273308**	**PZ315619**	**This study**
Agaricus padanus	**FJAU66735**	**China**	**PZ280490**	**PZ273309**	**PZ315620**	**This study**
Agaricus padanus	**FJAU66689**	**China**	**PZ280491**	**PZ273310**	**PZ315621**	**This study**
Agaricus padanus	**FJAU66666**	**China**	**PZ280492**	**PZ273311**	**PZ315622**	**This study**
Agaricus padanus	**FJAU66717**	**China**	**PZ280493**	**PZ273312**	**PZ315623**	**This study**
Agaricus padanus	**FJAU66879**	**China**	**PZ101588**	**PZ106762**	**PZ137624**	**This study**
Agaricus padanus	**FJAU66761**	**China**	**PZ280494**	**PZ273313**	**PZ315624**	**This study**
Agaricus padanus	**FJAU66698**	**China**	**PZ280495**	**PZ273314**	**PZ315625**	**This study**
Agaricus padanus	**FJAU66790**	**China**	**PZ280496**	**PZ273315**	**PZ315626**	**This study**
Agaricus padanus	**FJAU66655**	**China**	**PZ280497**	**PZ273316**	**PZ315627**	**This study**
Agaricus padanus	**FJAU66705**	**China**	**PZ280498**	**PZ273317**	**PZ315628**	**This study**
Agaricus padanus	**FJAU66703**	**China**	**PZ280499**	**PZ273318**	**PZ315629**	**This study**
Agaricus padanus	**FJAU66691**	**China**	**PZ280500**	**PZ273319**	**PZ315630**	**This study**
Agaricus padanus	**FJAU66656**	**China**	**PZ280501**	**PZ273320**	**PZ315631**	**This study**
Agaricus padanus	**FJAU66713**	**China**	**PZ280502**	**PZ273321**	**PZ315632**	**This study**
Agaricus padanus	**FJAU66674**	**China**	**PZ280503**	**PZ273322**	**PZ315633**	**This study**
Agaricus padanus	**FJAU66678**	**China**	**PZ280504**	**PZ273323**	**PZ315634**	**This study**
Agaricus padanus	**FJAU66700**	**China**	**PZ280505**	**PZ273324**	**PZ315635**	**This study**
Agaricus padanus	**FJAU66668**	**China**	**PZ280506**	**PZ273325**	**PZ315636**	**This study**
Agaricus padanus	**FJAU66675**	**China**	**PZ280507**	**PZ273326**	**PZ315637**	**This study**

Agaricus padanus	**FJAU66723**	**China**	**PZ280508**	**PZ273327**	**PZ315638**	**This study**
Agaricus padanus	**FJAU66695**	**China**	**PZ280509**	**PZ273328**	**PZ315639**	**This study**
Agaricus padanus	**FJAU66755**	**China**	**PZ280510**	**PZ273329**	**PZ315640**	**This study**
Agaricus padanus	**FJAU66696**	**China**	**PZ280511**	**PZ273330**	**PZ315641**	**This study**
Agaricus padanus	**FJAU66694**	**China**	**PZ280512**	**PZ273331**	**PZ315642**	**This study**
Agaricus padanus	**FJAU66859**	**China**	**PZ280513**	**PZ273332**	**PZ315643**	**This study**
Agaricus padanus	**FJAU66861**	**China**	**PZ280514**	**PZ273333**	**PZ315644**	**This study**
Agaricus padanus	**FJAU66644**	**China**	**PZ280515**	**PZ273334**	**PZ315645**	**This study**
Agaricus padanus	**FJAU66864**	**China**	**PZ101585**	**PZ106759**	**PZ137621**	**This study**
Agaricus padanus	**FJAU66918**	**China**	**PZ280516**	**PZ273335**	**PZ315646**	**This study**
Agaricus padanus	**FJAU66919**	**China**	**PZ280517**	**PZ273336**	**PZ315647**	**This study**
Agaricus padanus	**FJAU66742**	**China**	**PZ280518**	**PZ273337**	**PZ315648**	**This study**
Agaricus padanus	**FJAU66925**	**China**	**PZ101579**	**PZ106753**	**PZ137615**	**This study**
Agaricus padanus	**FJAU66882**	**China**	**PZ101591**	**PZ106765**	**PZ137627**	**This study**
Agaricus padanus	**FJAU66922**	**China**	**PZ101578**	**PZ106752**	**PZ137614**	**This study**
Agaricus padanus	58-02Lanconelli	France	KJ575604	-	-	[12]
Agaricus padanus	WZR2012903	China	KM657903	KR006616	KR006644	[12]
Agaricus padanus	19-02Lanconelli	France	KJ575603	-	-	[12]
Agaricus pallidobrunneus	ZRL2012358	China	KT951370	KT951471	KT951566	[9]
Agaricus pattersoniae	RWK1415	USA	AY943974	-	-	[12]
Agaricus phaeolepidotus	CA217 (INRA)	France	DQ185552	-	-	[9]
Agaricus pilosporus	LAPAG227	China	KT951425	-	-	[9]

Agaricus qilianensis	ZRL20161021 T	China	KY885114	KY885135	KY905105	[9]
Agaricus sinodeliciosus	**FJAU66739**	**China**	**PZ280385**	**PZ273204**	**PZ315515**	**This study**
Agaricus sinodeliciosus	**FJAU66810**	**China**	**PZ101536**	**PZ106710**	**PZ137572**	**This study**
Agaricus sinodeliciosus	**FJAU66943**	**China**	**PZ280386**	**PZ273205**	**PZ315516**	**This study**
Agaricus sinodeliciosus	**FJAU66926**	**China**	**PZ280387**	**PZ273206**	**PZ315517**	**This study**
Agaricus sinodeliciosus	**FJAU66748**	**China**	**PZ280388**	**PZ273207**	**PZ315518**	**This study**
Agaricus sinodeliciosus	**FJAU66860**	**China**	**PZ101541**	**PZ106715**	**PZ137577**	**This study**
Agaricus sinodeliciosus	**FJAU66762**	**China**	**PZ280389**	**PZ273208**	**PZ315519**	**This study**
Agaricus sinodeliciosus	**FJAU66773**	**China**	**PZ280390**	**PZ273209**	**PZ315520**	**This study**
Agaricus sinodeliciosus	**FJAU66857**	**China**	**PZ101540**	**PZ106714**	**PZ137576**	**This study**
Agaricus sinodeliciosus	**FJAU66805**	**China**	**PZ280391**	**PZ273210**	**PZ315521**	**This study**
Agaricus sinodeliciosus	**FJAU66833**	**China**	**PZ101537**	**PZ106711**	**PZ137573**	**This study**
Agaricus sinodeliciosus	**FJAU66774**	**China**	**PZ280392**	**PZ273211**	**PZ315522**	**This study**
Agaricus sinodeliciosus	**FJAU66942**	**China**	**PZ280393**	**PZ273212**	**PZ315523**	**This study**
Agaricus sinodeliciosus	**FJAU66923**	**China**	**PZ101545**	**PZ106719**	**PZ137581**	**This study**
Agaricus sinodeliciosus	**FJAU66924**	**China**	**PZ101544**	**PZ106718**	**PZ137580**	**This study**
Agaricus sinodeliciosus	**FJAU66807**	**China**	**PZ280394**	**PZ273213**	**PZ315524**	**This study**
Agaricus sinodeliciosus	**FJAU66945**	**China**	**PZ101548**	**PZ106722**	**PZ137584**	**This study**
Agaricus sinodeliciosus	**FJAU66811**	**China**	**PZ280395**	**PZ273214**	**PZ315525**	**This study**
Agaricus sinodeliciosus	**FJAU66920**	**China**	**PZ101543**	**PZ106717**	**PZ137579**	**This study**
Agaricus sinodeliciosus	**FJAU66809**	**China**	**PZ280396**	**PZ273215**	**PZ315526**	**This study**
Agaricus sinodeliciosus	**FJAU66862**	**China**	**PZ101542**	**PZ106716**	**PZ137578**	**This study**

Agaricus sinodeliciosus	**FJAU66840**	**China**	**PZ101538**	**PZ106712**	**PZ137574**	**This study**
Agaricus sinodeliciosus	**FJAU66856**	**China**	**PZ280397**	**PZ273216**	**PZ315527**	**This study**
Agaricus sinodeliciosus	**FJAU66944**	**China**	**PZ280398**	**PZ273217**	**PZ315528**	**This study**
Agaricus sinodeliciosus	**FJAU66817**	**China**	**PZ280399**	**PZ273218**	**PZ315529**	**This study**
Agaricus sinodeliciosus	**FJAU66750**	**China**	**PZ101534**	**PZ106708**	**PZ137570**	**This study**
Agaricus sinodeliciosus	**FJAU66782**	**China**	**PZ280400**	**PZ273219**	**PZ315530**	**This study**
Agaricus sinodeliciosus	**FJAU66744**	**China**	**PZ280401**	**PZ273220**	**PZ315531**	**This study**
Agaricus sinodeliciosus	**FJAU66747**	**China**	**PZ280402**	**PZ273221**	**PZ315532**	**This study**
Agaricus sinodeliciosus	**FJAU66842**	**China**	**PZ101539**	**PZ106713**	**PZ137575**	**This study**
Agaricus sinodeliciosus	**FJAU66806**	**China**	**PZ280403**	**PZ273222**	**PZ315533**	**This study**
Agaricus sinodeliciosus	**FJAU66772**	**China**	**PZ280404**	**PZ273223**	**PZ315534**	**This study**
Agaricus sinodeliciosus	WZR2012821 T	China	KM657906	-	KY905099	[12]
Agaricus sinotetrasporus	ZRL20161020 T	China	KY885113	KY885134	KY905104	[9]
Agaricus sipapuensis	RMC 1272 T	USA	KJ026950	-	-	[8]
Agaricus sp.	ZRL2085	China	KT951434	-	-	[9]
Agaricus sp.	ZRL2010099	China	KT951349	KT951479	KT951564	[9]
Agaricus subfloccosus	RWK 1994	USA	EU131640	-	-	[67]
Agaricus subperonatus	**FJAU66812**	**China**	**PZ280405**	**PZ273224**	**PZ315535**	**This study**
Agaricus subperonatus	**FJAU66813**	**China**	**PZ280406**	**PZ273225**	**PZ315536**	**This study**
Agaricus subperonatus	**FJAU66815**	**China**	**PZ280407**	**PZ273226**	**PZ315537**	**This study**
Agaricus subperonatus	**FJAU66818**	**China**	**PZ101549**	**PZ106723**	**PZ137585**	**This study**
Agaricus subperonatus	**FJAU66853**	**China**	**PZ280408**	**PZ273227**	**PZ315538**	**This study**

Agaricus subperonatus	**FJAU66849**	**China**	**PZ280409**	**PZ273228**	**PZ315539**	**This study**
Agaricus subperonatus	**FJAU66839**	**China**	**PZ101554**	**PZ106728**	**PZ137590**	**This study**
Agaricus subperonatus	**FJAU66819**	**China**	**PZ101550**	**PZ106724**	**PZ137586**	**This study**
Agaricus subperonatus	**FJAU66951**	**China**	**PZ101560**	**PZ106734**	**PZ137596**	**This study**
Agaricus subperonatus	**FJAU66952**	**China**	**PZ101561**	**PZ106735**	**PZ137597**	**This study**
Agaricus subperonatus	**FJAU66820**	**China**	**PZ280410**	**PZ273229**	**PZ315540**	**This study**
Agaricus subperonatus	**FJAU66824**	**China**	**PZ280411**	**PZ273230**	**PZ315541**	**This study**
Agaricus subperonatus	**FJAU66854**	**China**	**PZ280412**	**PZ273231**	**PZ315542**	**This study**
Agaricus subperonatus	**FJAU66826**	**China**	**PZ101552**	**PZ106726**	**PZ137588**	**This study**
Agaricus subperonatus	**FJAU66823**	**China**	**PZ101551**	**PZ106725**	**PZ137587**	**This study**
Agaricus subperonatus	**FJAU66831**	**China**	**PZ280413**	**PZ273232**	**PZ315543**	**This study**
Agaricus subperonatus	**FJAU66950**	**China**	**PZ101559**	**PZ106733**	**PZ137595**	**This study**
Agaricus subperonatus	**FJAU66837**	**China**	**PZ280414**	**PZ273233**	**PZ315544**	**This study**
Agaricus subperonatus	**FJAU66954**	**China**	**PZ101563**	**PZ106737**	**PZ137599**	**This study**
Agaricus subperonatus	**FJAU66855**	**China**	**PZ280415**	**PZ273234**	**PZ315545**	**This study**
Agaricus subperonatus	**FJAU66834**	**China**	**PZ280416**	**PZ273235**	**PZ315546**	**This study**
Agaricus subperonatus	**FJAU66836**	**China**	**PZ280417**	**PZ273236**	**PZ315547**	**This study**
Agaricus subperonatus	**FJAU66947**	**China**	**PZ101556**	**PZ106730**	**PZ137592**	**This study**
Agaricus subperonatus	**FJAU66821**	**China**	**PZ280418**	**PZ273237**	**PZ315548**	**This study**
Agaricus subperonatus	**FJAU66946**	**China**	**PZ101555**	**PZ106729**	**PZ137591**	**This study**
Agaricus subperonatus	**FJAU66838**	**China**	**PZ101553**	**PZ106727**	**PZ137589**	**This study**
Agaricus subperonatus	**FJAU66953**	**China**	**PZ101562**	**PZ106736**	**PZ137598**	**This study**

Agaricus subperonatus	**FJAU66949**	**China**	**PZ101558**	**PZ106732**	**PZ137594**	**This study**
Agaricus subperonatus	**FJAU66835**	**China**	**PZ280419**	**PZ273238**	**PZ315549**	**This study**
Agaricus subperonatus	**FJAU66948**	**China**	**PZ101557**	**PZ106731**	**PZ137593**	**This study**
Agaricus subperonatus	**FJAU66816**	**China**	**PZ280420**	**PZ273239**	**PZ315550**	**This study**
Agaricus subperonatus	RWK1733	USA	AF432900	-	-	[9]
Agaricus subsubensis	ATCC MYA-4432 T	USA	NR137710	-	-	[12]
Agaricus sylvaticus	LAPAG341	Spain	JF797178	-	-	[1]
Agaricus sylvaticus	**FJAU66959**	**China**	**PZ101639**	**PZ106813**	**PZ137675**	**This study**
Agaricus sylvaticus	**FJAU66999**	**China**	**PZ101646**	**PZ106820**	**PZ137682**	**This study**
Agaricus sylvaticus	**FJAU67000**	**China**	**PZ280428**	**PZ273247**	**PZ315558**	**This study**
Agaricus sylvaticus	**FJAU67019**	**China**	**PZ101649**	**PZ106823**	**PZ137685**	**This study**
Agaricus sylvaticus	**FJAU67015**	**China**	**PZ101648**	**PZ106822**	**PZ137684**	**This study**
Agaricus sylvaticus	**FJAU66997**	**China**	**PZ101645**	**PZ106819**	**PZ137681**	**This study**
Agaricus sylvaticus	**FJAU66964**	**China**	**PZ280429**	**PZ273248**	**PZ315559**	**This study**
Agaricus sylvaticus	**FJAU66979**	**China**	**PZ280430**	**PZ273249**	**PZ315560**	**This study**
Agaricus sylvaticus	**FJAU66995**	**China**	**PZ101644**	**PZ106818**	**PZ137680**	**This study**
Agaricus sylvaticus	**FJAU67008**	**China**	**PZ101647**	**PZ106821**	**PZ137683**	**This study**
Agaricus sylvaticus	**FJAU66972**	**China**	**PZ101641**	**PZ106815**	**PZ137677**	**This study**
Agaricus sylvaticus	HMJAU 67819	China	OR690307	-	-	[62]
Agaricus sylvaticus	LAPAG382	Spain	KM657929	KR006608	KR006637	[9]
Agaricus sylvaticus	ZRL2012013	China	KT951360	KT951500	KT951570	[9]
Agaricus sylvaticus	JFM AS	China	AJ133375	-	-	[9]

Agaricus sylvaticus var. occidentalis	SFSU F 021067 T	USA	NR 144984	-	-	[8]
Agaricus sylvaticus var. occidentalis	OMDL iNat 178323135	USA	PQ845147	-	-	[9]
Agaricus taeniatus	QLS60 T	China	KJ623317	KY885127	KY905097	[28]
Agaricus thujae	RHM6	USA	KJ577982	-	-	[8]
Agaricus tlaxcalensis	CA221	Mexico	EU363033	-	-	[58]
Agaricus trisulphuratus	LAPAF7	China	KM657924	KR006605	KR006634	[9]
Agaricus trisulphuratus	SWK079	China	KT951343	KT951472	KT951561	[9]
Agaricus variicystis	LD201234	China	KT951339	-	-	[9]
Agaricus xanthodermus	LAPAG387	China	KM657923	KP739802	KR006638	[32]
Agaricus xanthodermus	**FJAU66956**	**China**	**PZ101603**	**PZ106777**	**PZ137639**	**This study**
Agaricus xanthodermus	**FJAU66957**	**China**	**PZ280376**	**PZ273195**	**PZ315506**	**This study**
Agaricus xanthodermus	**FJAU66968**	**China**	**PZ101605**	**PZ106779**	**PZ137641**	**This study**
Agaricus xanthodermus	**FJAU66990**	**China**	**PZ101611**	**PZ106785**	**PZ137647**	**This study**
Agaricus xanthodermus	**FJAU67007**	**China**	**PZ101615**	**PZ106789**	**PZ137651**	**This study**
Agaricus xanthodermus	**FJAU67012**	**China**	**PZ101616**	**PZ106790**	**PZ137652**	**This study**
Agaricus xanthodermus	**FJAU67016**	**China**	**PZ101617**	**PZ106791**	**PZ137653**	**This study**
Agaricus xanthodermus	**FJAU67017**	**China**	**PZ101618**	**PZ106792**	**PZ137654**	**This study**
Agaricus xanthodermus	**FJAU67018**	**China**	**PZ101619**	**PZ106793**	**PZ137655**	**This study**
Agaricus xanthodermus	**FJAU67020**	**China**	**PZ101620**	**PZ106794**	**PZ137656**	**This study**
Agaricus xanthodermus	**FJAU67026**	**China**	**PZ101621**	**PZ106795**	**PZ137657**	**This study**
Agaricus xanthodermus	**FJAU67006**	**China**	**PZ101614**	**PZ106788**	**PZ137650**	**This study**
Agaricus xanthodermus	**FJAU67002**	**China**	**PZ101613**	**PZ106787**	**PZ137649**	**This study**

Agaricus xanthodermus	**FJAU66966**	**China**	**PZ280377**	**PZ273196**	**PZ315507**	**This study**
Agaricus xanthodermus	**FJAU66961**	**China**	**PZ280378**	**PZ273197**	**PZ315508**	**This study**
Agaricus xanthodermus	**FJAU66969**	**China**	**PZ280379**	**PZ273198**	**PZ315509**	**This study**
Agaricus xanthodermus	**FJAU66970**	**China**	**PZ280380**	**PZ273199**	**PZ315510**	**This study**
Agaricus xanthodermus	**FJAU66987**	**China**	**PZ101609**	**PZ106783**	**PZ137645**	**This study**
Agaricus xanthodermus	**FJAU66988**	**China**	**PZ101610**	**PZ106784**	**PZ137646**	**This study**
Agaricus xanthodermus	**FJAU66967**	**China**	**PZ101604**	**PZ106778**	**PZ137640**	**This study**
Agaricus xanthodermus	**FJAU66977**	**China**	**PZ280381**	**PZ273200**	**PZ315511**	**This study**
Agaricus xanthodermus	**FJAU66984**	**China**	**PZ280382**	**PZ273201**	**PZ315512**	**This study**
Agaricus xanthodermus	**FJAU66971**	**China**	**PZ101606**	**PZ106780**	**PZ137642**	**This study**
Agaricus xanthodermus	**FJAU66978**	**China**	**PZ280383**	**PZ273202**	**PZ315513**	**This study**
Agaricus xanthodermus	**FJAU66981**	**China**	**PZ101607**	**PZ106781**	**PZ137643**	**This study**
Agaricus xanthodermus	**FJAU66986**	**China**	**PZ101608**	**PZ106782**	**PZ137644**	**This study**
Agaricus xanthodermus	**FJAU66991**	**China**	**PZ101612**	**PZ106786**	**PZ137648**	**This study**
Agaricus xanthodermus	**FJAU67027**	**China**	**PZ101622**	**PZ106796**	**PZ137658**	**This study**
Agaricus xanthodermus	**FJAU66983**	**China**	**PZ280384**	**PZ273203**	**PZ315514**	**This study**
Agaricus xanthodermus	LAPAG461	China	KM657925	KR006612	-	[67]
